# Examination of novel 4-aminoquinoline derivatives designed and synthesized by a hybrid pharmacophore approach to enhance their anticancer activities

**DOI:** 10.1038/s41598-019-42816-4

**Published:** 2019-04-19

**Authors:** V. Raja Solomon, Sheetal Pundir, Hoyun Lee

**Affiliations:** 1 Health Sciences North Research Institute, 56 Walfor Road, Sudbury, Ontario P3E 2H3 Canada; 2 Department of Biology, Laurentian University, 935 Ramsey Lake Road, Sudbury, Ontario P3E 2C6 Canada; 3 Departments of Medicine, the Faculty of Medicine, the University of Ottawa, Ottawa, Ontario K1H 5M8 Canada

**Keywords:** Chemical libraries, Chemotherapy, Drug development

## Abstract

In an attempt to develop effective and potentially safe anticancer agents, thirty-six 4-aminoquinoline derived sulfonyl analogs were designed and synthesized using a hybrid pharmacophore approach. The cytotoxicity of these compounds was determined using three breast tumor cell lines (MDA-MB231, MDA-MB468 and MCF7) and two matching non-cancer breast epithelial cell lines (184B5 and MCF10A). Although most of the compounds were quite effective on the breast cancer cells, the compound 7-chloro-4-(4-(2,4-dinitrophenylsulfonyl)piperazin-1-yl)quinoline (**13**; VR23) emerged as potentially the most desirable one in this series of compounds. Data from the NCI-60 cancer panel screening show that compound **13** is effective on a wide range of different cancers. Importantly, compound **13** is needed up to 17.6-fold less doses to achieve the same IC_50_ against cancer than non-cancer cells (MDA-MB468 *vs* MCF10A), suggesting that it can potentially be less toxic to normal cells. Cancer cells formed multiple centrosomes in the presence of compound **13**, resulting in the cell cycle arrest at prometa-meta phase. This abnormality leads to eventual cell demise with sub-G1 DNA content typically shown with apoptotic cells. In addition, compound **13** also causes an increase in lysosomal volume in cancer but not in non-cancer cells, which may contribute at least in part to its preferential cancer cell-killing. The cancer cell-killing effect of compound **13** is highly potentiated when combined with either bortezomib or monastrol.

## Introduction

Taking advantage of the tremendous increase in knowledge of the molecular mechanisms and pathophysiology of cancer during the last few decades, much effort has recently been paid to increase cancer cell selectivity in chemotherapy^1–3^. However, most of the new compounds have not yet been therapeutically useful due mainly to low tumor selectivity^4^. Our current work is to potentially address this underlying problem.

**Figure 1:**
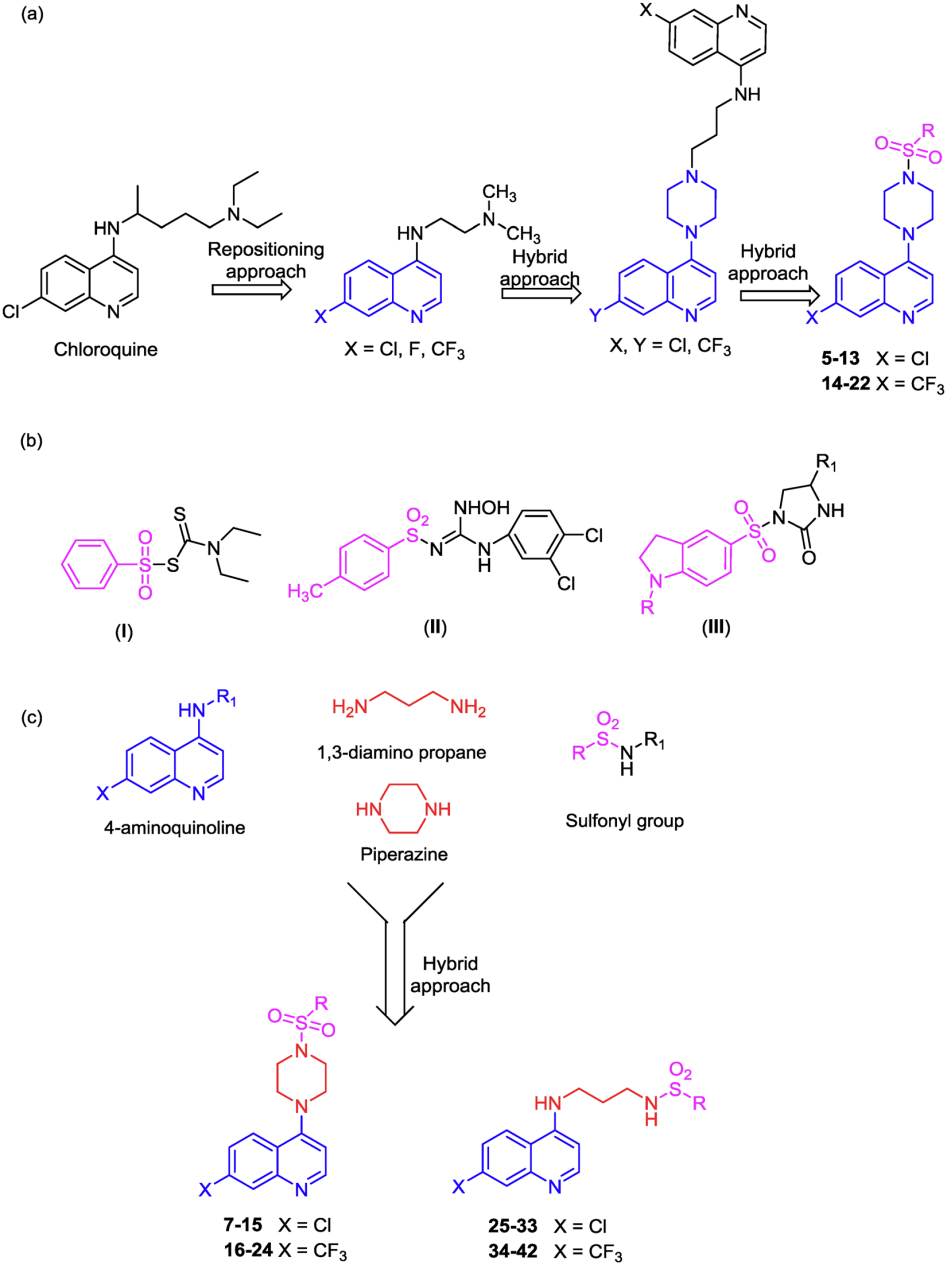
Design of hybrid compounds. (**a**) Chemical structures that we previously reported to have anticancer activity (see text). (**b**) Some sulfonyl analogs with anticancer activity. (**c**) The design of hybrid compounds.

We successfully applied previously two paralleled approaches in developing effective and selective anticancer agent: repositioning and hybrid pharmacophore approaches^5–11^. In these studies, we demonstrated that the anti-malarial drug chloroquine (CQ) could be effective on cancer cell-killing, highly synergistically if combined with radiation or Akt inhibitors^5,6^. Importantly, the cell-killing effect of CQ-Akt inhibitors is cancer-specific^5,6^, for which the lysosomotrophic property of CQ may play an important role. We then designed, synthesized and examined several CQ-analogs (Fig. 1I–III)^7–9^ by introducing linear alkyl side chains, dialkyl substitutions and/or heterocyclic ring substitutions on the lateral side chain^7–9^. We found that some of these compounds are indeed more effective than CQ^12^. Further SAR analysis indicated that more potent antigrowth/cell-killing effects on cancer cells (compared to non-cancer cells) could be achieved when the 7th position with a -Cl/CF_3_ group of the 4-(quinolin-4-yl)piperazin-1-yl ring system merged with potential pharmacophore groups^8^. Thus, the 4-piperazinylquinoline system may possess potent anticancer activity with higher tumor selectivity.

To develop further improved anticancer compounds, we have adopted a hybrid pharmacophore approach to synthesize 4-piperazinylquinoline-isatin hybrid compounds using a Mannich base reaction^9^. We found that the growth inhibition effects of 4-piperazinylquinoline-isatin hybrid compounds (Fig. 1a) were substantially more active on cancer than non-cancer cells, suggesting that tumor specificity can indeed be increased by a hybrid approach^9^. We also designed and synthesized hybrid compounds by linking the isatin ring system with the benzothiazole ring system by a Schiff base reaction. We found that some of these compounds showed much stronger antigrowth/cell-killing activity on breast cancer cells than non-cancer breast cells^13^. The present work is an extension of our ongoing effort towards developing novel hybrid pharmacophore compounds with higher efficacy and tumor specificity.

Pharmacological agents containing a sulfonyl pharmacophore have been widely used as antibacterial, anticarbonic anhydrase, antiviral, and hypoglycemic agents^14–18^. Studies carried out by *in vitro* and/or *in vivo* approaches showed that sulfonyl derivatives shown in Fig. 1b (1–III) also contain substantial antitumor activity^19–22^.

**Figure 2:**
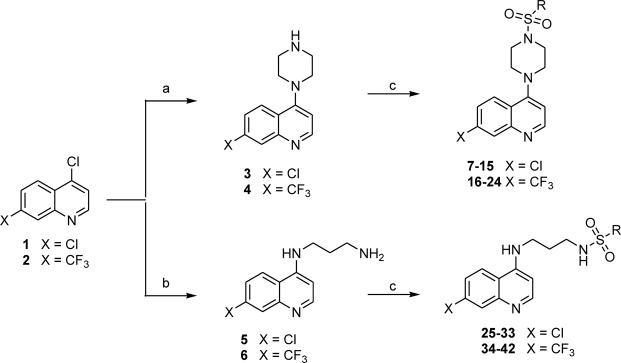
Schematic presentation of the synthesis of 4- aminoquinoline derived analogs. (**a**) Piperazine, Triethylamine, 120–130 °C for 6 h; (**b**) 1,3-Diaminopropane, 120–130 °C for 6 h; and (**c**) R_1_-sulfonyl chloride, Triethylamine, THF, RT, 4 h.

These previous findings gave impetus to our cancer drug research by further augmenting the realization that rational choice of inputs based on the known 4-aminoquinoline scaffold and the sulfonamide pharmacophore could lead to molecules with desirable anticancer property. To link these two in a single molecule, we used a linear side chain of 1,3-diamino propane as well as a rigid ring of the piperazin-1-yl moiety as a linker. We then synthesized 4-aminoquinoline derived sulfonamide conjugate molecules (Figs 1c and 2), and examined their growth inhibition/cell-killing effects on three human breast tumor lines and two matching non-cancer breast cell lines. Compound **13**, the most desirable one in this series was further examined to gain understanding of its molecular mechanisms and effects on other cancer cells using the NCI-60 cancer panel.

## Results and Discussion

### Chemistry

The amino components (**3–4** and **5–6**) used in the present study were prepared by aromatic nucleophilic substitution on 4-chloro-7-substituted-quinoline with excess of piperazine or 1,3-diamino propane in triethyl amine as reported earlier^8^. The amino component (**3–4** and **5–6**) underwent sulfonation by alkyl/aryl/heteroaryl sulfonyl chloride in THF at room temperature for 4 h to furnish desired sulfonyl analogs (**7–24** and **25–42**) in very good yield. Spectroscopic data unambiguously verified the synthesized compound structures. IR spectra generally showed a strong absorption band ranging from 1160 to 1175 cm^−1^ for SO_2_ in their respective compounds (**7–24**). The IR spectrum of compounds (**25–42**) showed broad absorption bands around 3275–3305 cm^−1^ for NH (NHSO_2_), and 1170–1190 cm^−1^ for SO_2_ (NHSO_2_). These compounds also exhibited appropriate peaks at corresponding δ ppm in their ^1^H-NMR, ^13^C-NMR spectra which were in conformity with the assigned structures. ^1^H-NMR spectrum of compounds (**7–24)** showed the characteristic singlets around δ 2.94–3.36 ppm for piperazinyl CH_2_ (i.e. N(C*H*_2_CH_2_)_2_N-Ar) and δ 3.38–3.71 ppm for piperazinyl CH_2_ protons (i.e. N(CH_2_C*H*_2_)_2_N-Ar). In particular, compounds (**25–42**) displayed the propyl alkyl protons appeared as multiplets between δ 1.49–3.52 ppm. In ^13^C-NMR for the representative **7–42**, we have observed most characteristic signals appeared aromatic carbons around δ 99–160 ppm and aliphatic carbons around δ 27–52 ppm. The mass spectra of all the synthesized compounds were in conformity with their assigned structures. The mass spectra of these compounds showed molecular ion peaks corresponding to their molecular formulas. Elemental (C, H, N) analysis satisfactorily confirmed elemental compositions and the purity of the synthesized compounds.

### Antigrowth/antiproliferative effects of the compounds on cancer and non-cancer cells

The antigrowth effects of 4-piperazinylquinoline sulfonyl analogs on human breast tumor cells was initially evaluated using MDA-MB468 (a PTEN defective, EGFR positive breast adenocarcioma), MDA-MB231 (p53 and pRB mutated, triple-negative breast carcinoma), and MCF7 (p53+/−, invasive ductal breast carcinoma) cell lines. In addition, the cytotoxicity of all the compounds was also evaluated with the 184B5 and MCF10A non-cancer immortalized breast epithelial cell lines, to determine if these newly synthesized compounds have differential cytotoxic effects on cancer and non-cancer cells. The panel of three breast cancer cell lines with different genetic back ground and the well-studied two non-cancer immortalized breast cell lines would provide insights into the efficacy and toxicity of the test compounds in the different genetic background and at different stages of tumor development. The dose response of each cell line was established by determining the number of viable cells after 48 h of continuous drug treatment against seven different concentrations (100 µM to 0.0064 µM) of each compound. The reading of sulphorodamine B (SRB) staining is known to accurately reflect the level of total cellular macromolecules/cell growth/proliferation^6,23^. The GI_50_ concentration of each compound was calculated with reference to a control sample, which represents the concentration that results in a 50% decrease in cell number/growth/proliferation after 48 h incubation in the presence of a compound (examples shown in Supplementary Fig. S1). Table 1 shows the GI_50_ values (μM), the concentration of each compound required to inhibit the growth of each cell line by 50%. The data for CQ and cisplatin were included as references. The antigrowth activity of 4-piperazinylquinoline derived sulfonyl compounds against the three breast cancer cell lines (MDA-MB231, MDA-MB468, and MCF7) revealed that most of the compounds possess growth inhibitory property at the micromolar range to reach GI_50_ values. The differences in the GI_50_ values may be attributable to such factors as the nature of the sulfonyl group and the halogen substitution on the 7^th^ position of the 4-piperazinylquinoline ring system, and the genetic and biochemical background of the cell lines.

**Table 1:** Antiproliferative activity of 4-piperazinylquinoline derived sulfonyl analogs **(7–42)** on human breast cancer cells and non-cancer cells.

Lab code	Compounds^a^	X	R	GI_50_ (μM)^b,c^				
				MB231	MB468	MCF7	184B5	MCF10A
VR 20	**7**	Cl	Methyl	42.7 ± 0.8	35.1 ± 0.8	22.9 ± 0.6	11.5 ± 0.2	15.3 ± 0.1
VR 22	**8**	Cl	Tosyl	34.1 ± 0.7	28.8 ± 0.7	16.1 ± 0.6	47.5 ± 0.9	44.4 ± 0.7
VR 34	**9**	Cl	Biphenyl	26.2 ± 0.6	18.2 ± 0.5	9.2 ± 0.2	16.4 ± 0.2	12.4 ± 0.2
VR 24	**10**	Cl	4-Chlorophenyl	25.5 ± 0.7	16.0 ± 0.6	7.6 ± 0.1	12.1 ± 0.2	8.7 ± 0.1
VR 35	**11**	Cl	2,4-Dichlorophenyl	40.6 ± 0.8	21.6 ± 0.6	13.5 ± 0.2	12.4 ± 0.1	8.9 ± 0.2
VR 25	**12**	Cl	3-Nitrophenyl	34.7 ± 0.7	25.1 ± 0.7	14.8 ± 0.2	13.3 ± 0.2	15.5 ± 0.2
VR 23	**13**	Cl	2,4-Dinitrophenyl	3.4 ± 0.1	0.7 ± 0.1	2.3 ± 0.1	9.0 ± 0.1	12.3 ± 0.1
VR 37	**14**	Cl	*N,N*-Dimethylnaphthalenyl	35.0 ± 0.8	27.5 ± 0.7	22.3 ± 0.2	28.3 ± 0.3	32.2 ± 0.3
VR 36	**15**	Cl	Thiophenyl-2-carboxylic acid methyl ester	40.4 ± 0.8	30.2 ± 0.7	22.4 ± 0.2	15.2 ± 0.2	14.6 ± 0.1
VR 38	**16**	CF_3_	Methyl	44.4 ± 0.9	28.6 ± 0.7	25.6 ± 0.3	93.9 ± 0.9	75.8 ± 0.8
VR 39	**17**	CF_3_	Tosyl	42.7 ± 0.8	36.5 ± 0.8	20.8 ± 0.2	12.9 ± 0.1	10.5 ± 0.1
VR 43	**18**	CF_3_	Biphenyl	27.2 ± 0.6	20.5 ± 0.5	14.8 ± 0.2	19.1 ± 0.2	15.5 ± 0.4
VR 40	**19**	CF_3_	4-Chlorophenyl	41.4 ± 0.8	34.9 ± 0.8	27.4 ± 0.3	21.8 ± 0.3	13.6 ± 0.2
VR 45	**20**	CF_3_	2,4-Dichlorophenyl	20.3 ± 0.5	18.6 ± 0.5	16.7 ± 0.2	20.4 ± 0.2	15.6 ± 0.2
VR 41	**21**	CF_3_	3-Nitrophenyl	32.2 ± 0.6	18.6 ± 0.6	9.4 ± 0.3	17.7 ± 0.2	15.4 ± 0.1
VR 42	**22**	CF_3_	2,4-Dinitrophenyl	24.3 ± 0.6	19.2 ± 0.6	10.8 ± 0.1	37.8 ± 0.4	35.4 ± 0.5
VR 44	**23**	CF_3_	*N,N*-dimethylnaphthalenyl	22.1 ± 0.6	19.1 ± 0.6	12.9 ± 0.2	12.0 ± 0.2	15.4 ± 0.5
VR 46	**24**	CF_3_	Thiophenyl-2-carboxylic acid methyl ester	34.7 ± 0.7	23.9 ± 0.6	16.0 ± 0.2	15.9 ± 0.1	15.8 ± 0.1
VR 21	**25**	Cl	Methyl	40.9 ± 1.5	28.6 ± 1.0	23.1 ± 0.9	23.3 ± 0.9	39.1 ± 1.2
VR 26	**26**	Cl	Tosyl	6.2 ± 0.5	5.8 ± 0.4	5.2 ± 0.5	9.6 ± 0.5	12.2 ± 0.6
VR 52	**27**	Cl	Biphenyl	4.6 ± 0.4	4.5 ± 0.4	2.5 ± 0.2	2.4 ± 0.1	2.6 ± 0.1
VR 33	**28**	Cl	4-Chlorophenyl	11.8 ± 0.9	8.3 ± 0.7	4.3 ± 0.3	6.4 ± 0.3	9.7 ± 0.1
VR 66	**29**	Cl	2,4-Dichlorophenyl	6.8 ± 0.6	3.6 ± 0.1	4.0 ± 0.2	3.6 ± 0.2	6.8 ± 0.4
VR 32	**30**	Cl	3-Nitrophenyl	20.4 ± 1.2	9.2 ± 0.7	8.6 ± 0.4	15.8 ± 0.9	31.1 ± 1.1
VR 27	**31**	Cl	2,4-Dinitrophenyl	8.9 ± 0.7	7.4 ± 0.2	6.2 ± 0.3	9.1 ± 0.6	19.8 ± 0.9
VR 65	**32**	Cl	*N,N*-Dimethylnaphthalenyl	12.7 ± 1.0	4.6 ± 0.4	2.5 ± 0.1	1.7 ± 0.1	2.6 ± 0.1
VR 67	**33**	Cl	Thiophenyl-2-carboxylic acid methyl ester	6.1 ± 0.5	4.1 ± 0.3	4.2 ± 0.2	15.7 ± 0.4	16.5 ± 0.8
VR 57	**34**	CF_3_	Methyl	55.9 ± 1.6	30.4 ± 1.0	25.3 ± 1.3	59.5 ± 1.6	85.0 ± 1.2
VR 56	**35**	CF_3_	Tosyl	6.4 ± 0.5	6.2 ± 0.4	6.9 ± 0.5	12.2 ± 0.9	10.9 ± 0.9
VR 61	**36**	CF_3_	Biphenyl	6.0 ± 0.4	4.2 ± 0.2	3.6 ± 0.3	2.8 ± 0.1	2.2 ± 0.3
VR 60	**37**	CF_3_	4-Chlorophenyl	12.8 ± 0.9	10.3 ± 0.6	5.4 ± 0.3	7.6 ± 0.4	17.4 ± 0.1
VR 62	**38**	CF_3_	2,4-Dichlorophenyl	7.0 ± 0.6	3.9 ± 0.1	5.2 ± 0.3	3.2 ± 0.1	5.3 ± 0.3
VR 58	**39**	CF_3_	3-Nitrophenyl	15.7 ± 1.0	9.1 ± 0.6	8.3 ± 0.5	8.0 ± 0.8	10.6 ± 0.9
VR 59	**40**	CF_3_	2,4-Dinitrophenyl	8.9 ± 0.8	8.5 ± 0.4	5.6 ± 0.3	15.9 ± 0.9	22.3 ± 0.8
VR 63	**41**	CF_3_	*N,N*-dimethylnaphthalenyl	14.2 ± 1.0	9.7 ± 0.3	7.7 ± 0.6	3.7 ± 0.2	6.5 ± 0.3
VR 64	**42**	CF_3_	Thiophenyl-2-carboxylic acid methyl ester	7.5 ± 0.6	4.6 ± 0.2	4.3 ± 0.2	12.1 ± 0.5	14.4 ± 0.8
	Chloroquine			22.5 ± 1.4	28.6 ± 1.3	38.4 ± 1.2	76.1 ± 1.1	81.26 ± 1.3
	Cisplatin			23.7 ± 0.2	31.0 ± 0.5	25.8 ± 0.4	25.5 ± 0.4	51.51 ± 0.9

^a^For chemical structures, see Figs 1 and 2. ^b^GI_50_ values, determined at 48 h post-treatment, were calculated from sigmoidal dose response curves (variable slope), which were generated with GraphPad Prism V. 4.02. ^c^Values are mean of triplicates of at least two independent experiments.

The structure-activity relationship (SAR) analysis suggests that the compounds derived from the 7-chloro-4-piperazinylquinoline ring system (except compounds **11**, **12**, **14** and **15**) show generally better antigrowth activity on breast cancer cells than those derived from bioisoteric replacement of 7-chloro group with 7-trifluoromethyl substitution on the 4-aminoquinoline ring system (Table 1). The antigrowth effects generally increase in the following order: MCF7 > MDA-MB468 > MDA-MB231 cancer cells.

The introduction of a methyl group on sulfonyl analogs (compounds **7** and **16**) shows less antigrowth effects, suggesting that methyl substitution is not favorable for the anticancer activity of sulfonyl analogs regardless the presence of a -Cl or -CF_3_ group at the 7^th^ position. In contrast, the introduction of the liphophilic tosyl group substitution (compounds **8** and **17**) on sulfonyl analogs resulted in a substantial increase in the antigrowth activity on MDA-MB231, MDA-MB468 and MCF7 cells. In addition, the introduction of a bulky liphophilic biphenyl group on sulfonyl analogs (compounds **9** and **18**) showed an increase in antigrowth activity on all three breast cancer cell lines. These data clearly suggest that bulky lipophilic substitutions are advantageous for the increase in the antigrowth activity on these cancer cell lines.

The 4-chloro phenyl substituted hybrid compound **10** shows an increase in antigrowth activity by 3-fold on MCF7 in comparison to the methyl substituted compound **7**. Furthermore, the introduction of 2,4-dichloro phenyl substitution (**11**) shows a decrease in antigrowth activity on all cell lines examined. However, this is completely opposite in compounds derived from the 7-trifluoro-4-piperazinylquinoline ring system, in that 2,4-dichloro phenyl substituted hybrid compound (**20**) shows an increase in antigrowth activity by 2-fold on MDA-MB231 in comparison to the methyl substituted compound (**16**). Furthermore, the introduction of 4-chloro phenyl substitution (**10**) shows a decrease in antigrowth activity on all three cell lines examined.

The introduction of 3-nitro phenyl substitution (compounds **12** and **21**) was generally not effective. The 2,4-dinitro phenyl substitution (**13** and **22**) shows an increase in potency by 1.1–12.47-fold on MCF7 cells, compared to mono nitro substituted compounds (**12** and **21**). In this case, a -Cl group at the 7^th^ position (compound **13**) appears to be more favorable than a -CF_3_ group (compound **22**), since the former shows higher antigrowth effects on cancer cells than non-cancer cells. These results suggest that the disubstituted electron withdrawing groups in the phenyl ring system are generally more favorable for anticancer activity.

The introduction of *N,N*-dimethylnaphthalenyl (compounds **14** and **23**) and thiophenyl-2-carboxylic acid methyl ester substitutions (compounds **15** and **24**) on sulfonyl analogs are less potent, suggesting that these modifications are not favorable for anticancer activity of sulfonyl analogs, regardless the presence of a -Cl or a -CF_3_ group at the 7^th^ position.

SAR analysis suggests that the compounds derived from the 1,3-diamino propane-linked 7-chloro-4-aminoquinoline ring system (except compound **28** and **30**) show generally better antigrowth activity on breast cancer cells than those derived from bioisoteric replacement of a 7-chloro group with a 7-trifluoromethyl substitution on the 4-aminoquinoline ring system (Table 1). The introduction of a methyl group on sulfonamide analogs (compounds **25** and **34**) is less potent, suggesting that a methyl substitution is not favorable for the anticancer activity of sulfonamide analogs, regardless the presence of a -Cl or a -CF_3_ group at the 7^th^ position. In contrast, the introduction of the tosyl group substitution (compounds **26** and **35**) on sulfonamide analogs resulted in a substantial increase in potency on MDA-MB231, MDA-MB468 and MCF7 cells. The introduction of a bulky biphenyl group (compounds **27** and **36**) on sulfonamide analogs also shows an increase in potency on all three cancer cell lines. These data clearly suggest that lipophilic and bulky substitutions are advantageous for the increase in potency against cancer cells.

Two 4-chloro phenyl substituted hybrid compounds (**28** and **37**) show increases in potency by 3.5–4.4-fold on MDA-MB231 in comparison to methyl substituted compounds (**25** and **34**). Furthermore, the introduction of 2,4-dichloro phenyl substitution (compounds **29** and **38**) shows increases in potency on all three cancer cell lines examined, which are generally more effective than 4-chloro phenyl substituted compounds (**28** and **37**). The introduction of a 3-nitro phenyl substitution (compounds **30** and **39**) is generally not very effective. The 2,4-dinitro phenyl substitution (**31** and **40**) shows an increase in antigrowth activity by 1.7–2.3-fold against MDA-MB231, compared to mono nitro substituted compounds (**30** and **39**). In this case, a -CF_3_ group at the 7^th^ position (compound **20**) appears to be more favorable than a -Cl group (compound **31**), since the former shows higher antigrowth effects on cancer than non-cancer cells. These results suggest that the disubstituted electron withdrawing groups in the phenyl ring system are generally more favorable for anticancer activity.

The introduction of the *N,N*-dimethylnaphthalenyl substitution on the 7-chloro-4-aminoquinoline sulfonamide compound (**32**) led to a 15.7-fold increase in potency against MCF7 (GI_50_ = 2.45 µM), compared to CQ (GI_50_ = 38.44 µM). However, this modification results in the loss of preferential antigrowth effects on cancer cells, as they also show effective antigrowth effects on non-cancer cells. The compounds derived from the thiophenyl-2-carboxylic acid methyl ester substitution on the 7-chloro (**33**) and 7-trifluoromethyl (**42**) substituted 4-aminoquinoline sulfonamide show a substantial increase in potency against all three breast cancer cell lines. The compound **33** is particularly effective on cancer cells, as its GI_50_ values are 5.97, 4.18 and 4.22 µM on MDA-MB231, MDA-MB468 and MCF7, respectively (Table 1). This demonstrates that the antigrowth effects of compound **33** on all three breast cancer cell lines are 3.7-fold to 9.1-fold more effective than the parental CQ (Table 1). Furthermore, compound **33** is 4.0-fold (MDA-MB231), 5.4-fold (MDA-MB468), 6.1-fold (MCF7) more effective than cisplatin, one of the most widely prescribed anticancer agents. Compared to its effect on cancer cells, compound **33** is less effective on non-cancer breast cell lines, as its GI_50_ values on the two non-cancer cell lines are 15.71 µM (184B5) to 16.48 µM (MCF10A) (Table 1). In contrast, the cytotoxic effects of cisplatin on cancer cells and non-cancer cells are similar. It is important to note that compounds **8**, **13**, **14**, **16**, **21**, **22**, 2**6**, **35**, **40**, and **42** show promise, as they possess effective and preferential antigrowth effects on cancer cells (Table 1). It may also appropriate to mention that 4-piperazinylquinoline derived sulfonyl compounds (**8**, **13**, **14**, **16**, **21** and **22)** are more active than previously reported analogs^7,8,17^. Overall, it is quite clear that a piperazinyl linker is favorable for the increase in anticancer activity, probably due to their rigid nature.

**Figure 3:**
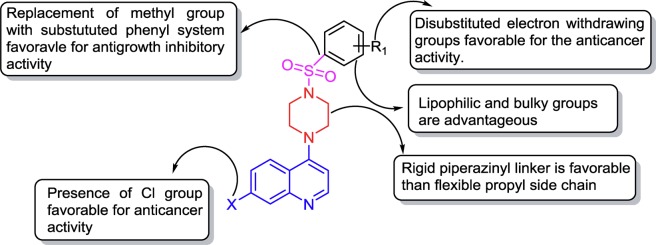
Summary of SAR analysis.

Many sulfonyl compounds show selective cytotoxicity on cancer over normal cells. This selectivity is likely the result of the heterocyclic attachment to the sulfonyl pharmacophore^17,20,24^. In the present study sulfonyl and sulfonamide groups are linked with “Sui-generic” pharmacophore of 4-aminoquinoline, therefore some of compounds including **8**, **13**, **14**, **16**, **21**, **22**, **26**, **35**, **40**, and **42** show selective anti-proliferative effect against cancer cells. SAR analysis discussed above is summarized in Fig. 3.

Among this series the compound **13** (a.k.a.VR23^25^) is particularly effective on cancer cells, as its GI_50_ values are 3.4 ± 0.1, 0.7 ± 0.1 and 2.3 ± 0.1 µM on MDA-MB231, MDA-MB468 and MCF7 cells, respectively (Table 1). These data from SRB were further confirmed by clonogenic assay, as examples are shown in Supplementary Fig. S1. Our data demonstrate that the antigrowth effects of compound **13** against cancer cell lines are 4.1- to 16.6-fold more effective than the parental CQ (Table 1). Compound **13** is also 4.3-fold (MDA-MB231), 9.2-fold (MDA-MB468), and 11.1-fold (MCF7) more effective than cisplatin. Compared to its effect on cancer cells, compound **13** is much less effective on non-cancer breast cell lines, as its GI_50_ values are 9.0 ± 0.1 μM and 12.3 ± 0.1 μM against 184B5 and MCF10A, respectively (Table 1). Furthermore, data from screening of the NCI-60 cancer panel show that compound **13** is effective on many different human cancers (Supplementary Fig. S2). Together, these data demonstrate that compound **13** has substantial potential as an effective and potentially safe anticancer agent. Therefore, we examined its effects on several cellular and molecular biological aspects to gain a better understanding about its mode of function.

### Cancer cells are arrested at prometa-meta phase in the presence of compound 13 due to abnormal mitotic chromosomal arrangement, eventually leading to cell death with sub-G1 DNA content

**Figure 4:**
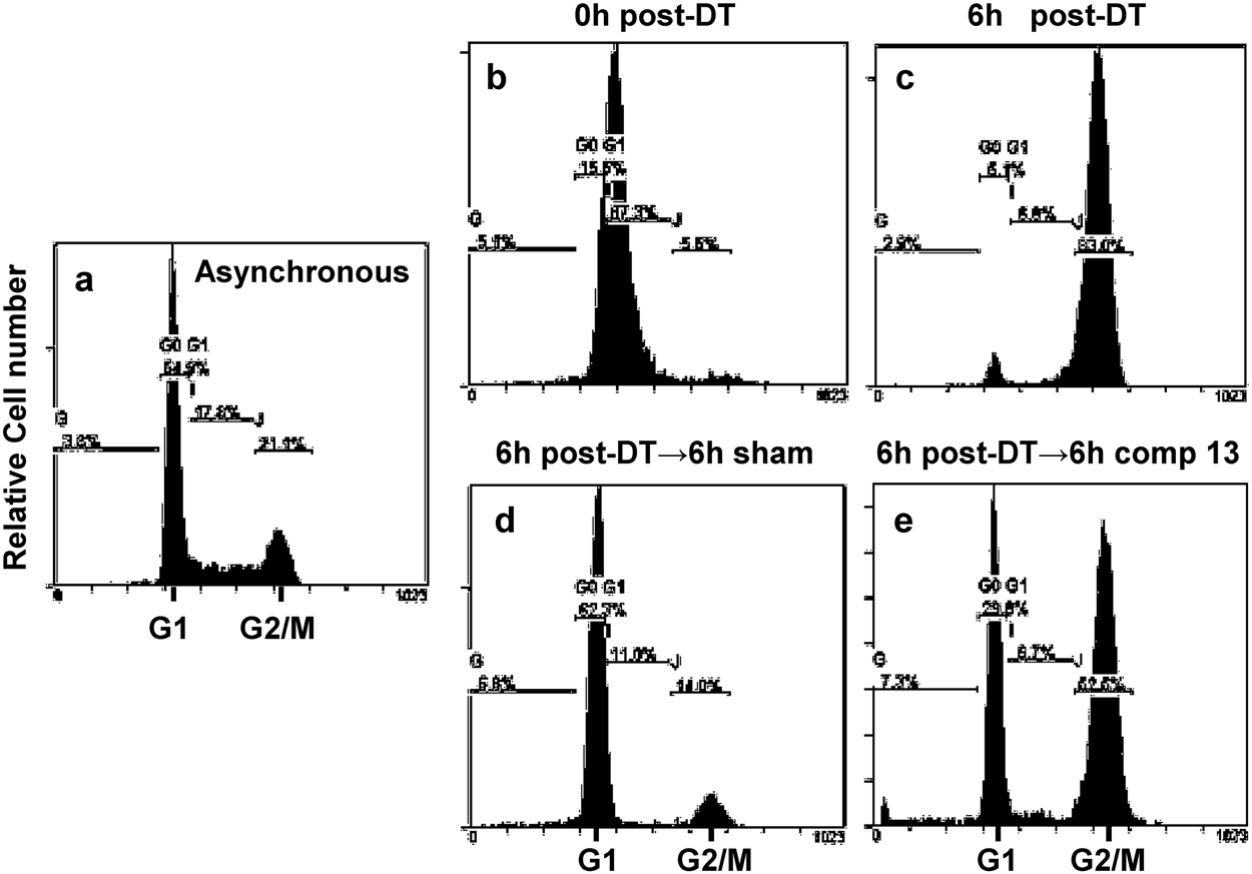
Compound **13** (VR23) causes a cell-cycle arrest at M phase. (**a**) Cell cycle profile of asynchronous HeLa S3 cells (a control profile). (**b**) HeLa cells were synchronized at the beginning of S phase by double thymidine (DT) block as described previously^31^. (**c**) Cells synchronized by DT treatment were released into drug-free complete medium for 6 h, at which time most cells were at late-S to G2/M. (**d**) Cells were continued to incubate in drug-free medium for additional 6 h, at which time most cells were already in G1 of the next cell cycle. (**e**) The same as the sample in panel d, but cells were incubated in the presence of 10 µM compound **13**.

**Figure 5:**
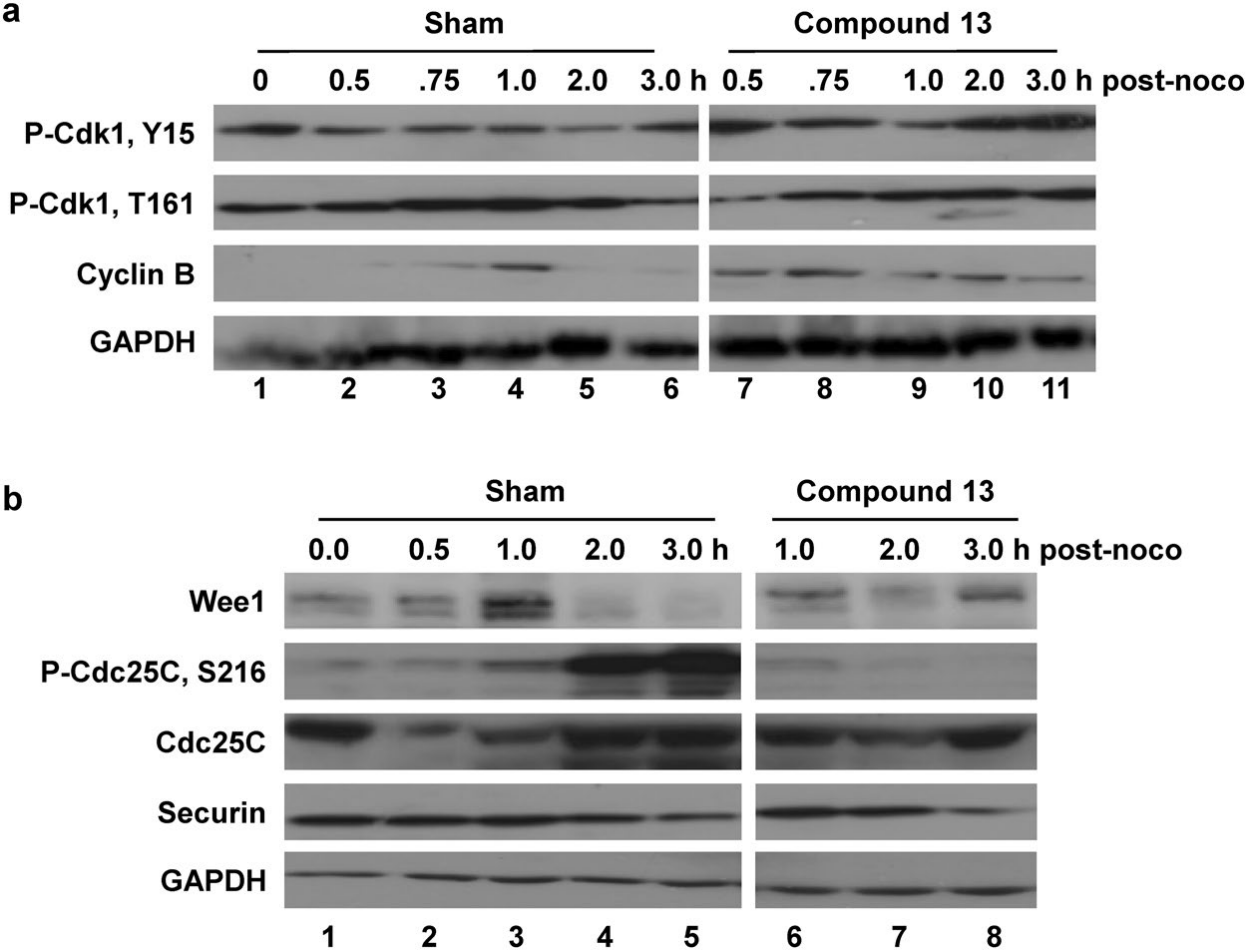
Compound **13**-treated cells are arrested at prometa-metaphase through the inactivation of Cdk1. HeLa S3 cells were synchronized at G2/M phase by incubating in 50 ng/ml nocodazole (noco) for 18 h. The cells were then released into complete medium at time 0 h in the absence (Sham) or presence of 10 µM compound **13**. Samples were then taken at the indicated times post-nocodazole. (**a**) Phosphorylation of Cdk1 on Tyr15 was reduced at 1 h, but dramatically increased at 2–3 h post-nocodazole arrest point. (**b**) Cdk1 may be inactivated by the combination of a high level of Wee1 kinase and inactivation of Cdc25C. The relative intensity of band signals are quantified by densitometry and presented the results in Supplementary Fig. S3.

To gain a better understanding about the molecular mechanism of compound **13** on antigrowth/cell-killing, HeLa S3 cells synchronized at G1/S by double thymidine treatment (DT) (Fig. 4b) were released into complete medium for 6 h, at which time most cells are at late S-G2/M phase (Fig. 4c). The cells were then maintained for additional 6 h in the absence (Fig. 4d) or presence of 10 μM compound **13** (Fig. 4e). More than 62% of cells progressed into G1 of the next cell cycle in the sham control (Fig. 4d). In contrast, more than 62% of cells were still in G2/M in the presence of compound **13**, indicating that the compound causes cell cycle arrest at G2/M (Fig. 4e). To gain further insights, we examined the active/inactive status of proteins involved in the regulation of the G2-M-G1 transition. Western blot data in Fig. 5a (and Supplementary Fig. S3 for quantitation) show that, unlike in the sham control, both Thr161 and Tyr15 residues on Cdk1 kinase are highly phosphorylated at 2–3 h post-nocodazole in the presence of compound **13**. Interestingly, the phosphorylation on Tyr15 was substantially down-regulated at 1 h post-nocodazole, prior to the dramatic increase in phosphorylation at 2–3 h post-nocodazole. Together, these data suggest that cancer cells briefly progressed toward M phase (i.e., 1 h post-nocodazole) even in the presence of compound **13**; however, they were then arrested at metaphase due to the inactivation of Cdk1, at the space between Thr161 phosphorylation and Tyr15 dephosphorylation. Cdc25C phosphatase is very active at 2–3 h post-nocodazole in the sham control but almost completely inactive in the presence of compound **13**. This indicates that the continuous activation of Cdk1 after 1 h post-nocodazole is blocked in cells treated with compound **13**, probably due to the failure of the Cdc25C-mediated Tyr15 dephosphorylation (Fig. 5b). Similarly, Wee1 is mostly inactive by 3 h post-nocodazole in sham control; however, it is very active in the presence of compound **13**. This also suggests that cells try to block further cell cycle progression beyond early M phase by inactivating Cdk1 activity. Since the level of securin is low, cell cycle is likely arrested prior to the chromatin separation stage in the presence of compound **13**.

**Figure 6:**
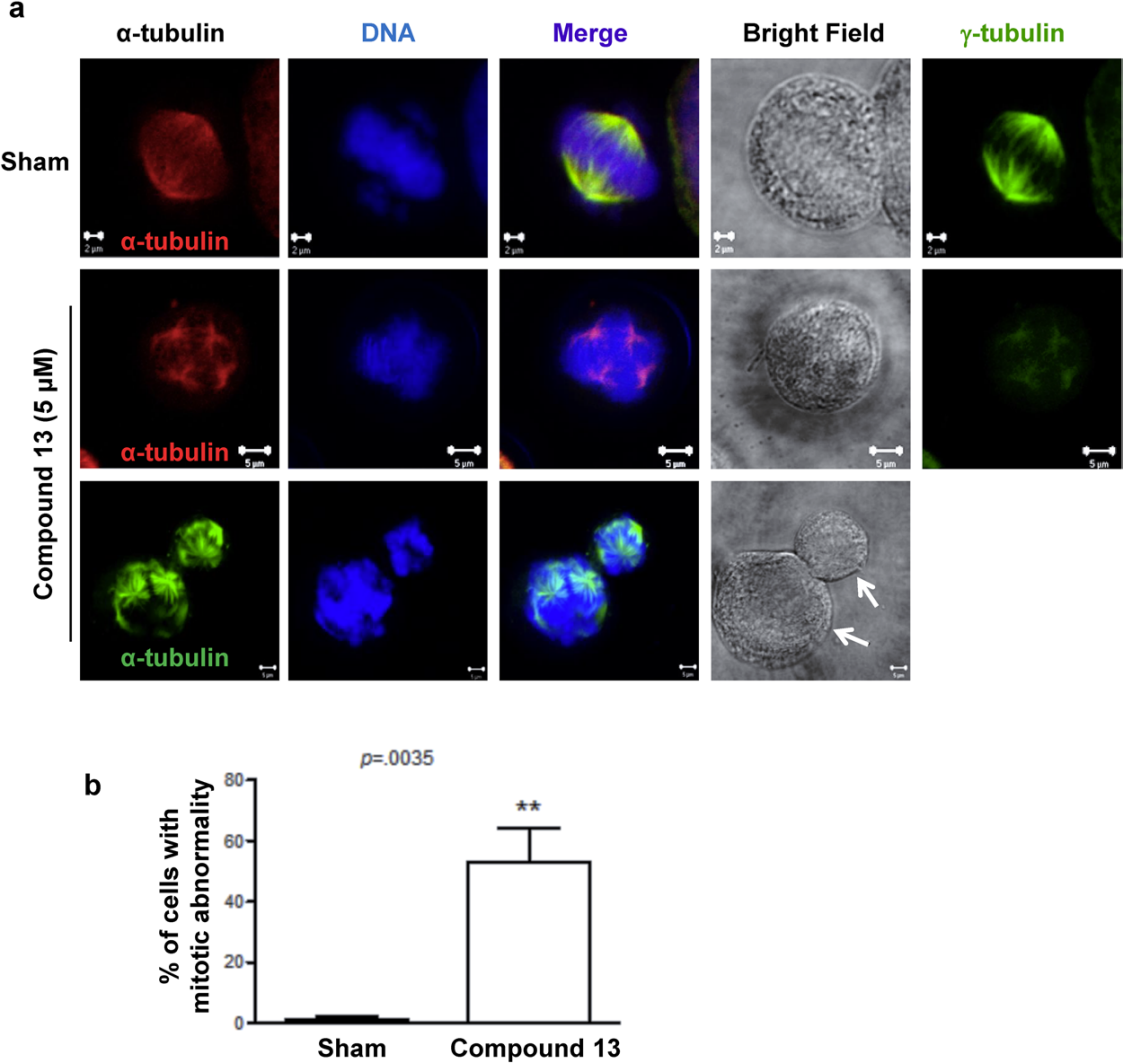
Compound **13** causes the formation of multiple spindle poles and uneven segregation. (**a**) HeLa S3 cells were synchronized at G2/M by nocodazole, followed by incubation for 6 h in drug-free medium in the absence (Sham control) or presence of 5 μM of compound **13**. Note the presence of uneven cell sizes (arrows) which were likely generated as a result of nondisjuctional chromosome segregation during mitosis. (**b**) Multiple spindle phenotypes were seen in 44.4% of compound **13**-treated cells. In contrast, the untreated control sample contains very few cells with abnormal spindles.

To gain further insight into the cellular mechanism involved in the cell cycle arrest, we examined cell morphology in the context of mitotic progress. We found that overall 44% of mitotic cells contained multiple centrosomes and other abnormalities in mitotic chromosomal arrangement (Fig. 6a,b; Supplementary Fig. S4). Many of these cells are apparently arrested at the spindle checkpoint stage. Even those cells undergo cell division (probably after prolonged arrest) often segregated with uneven cell sizes (arrows in Fig. 6a). Together, these data indicate that compound **13** causes the formation of multiple centrosomes, which results in defect in the arrangement of normal mitotic chromosomes, eventually leading to cell death.

### Compound 13 causes an increase in lysosome volume

**Figure 7:**
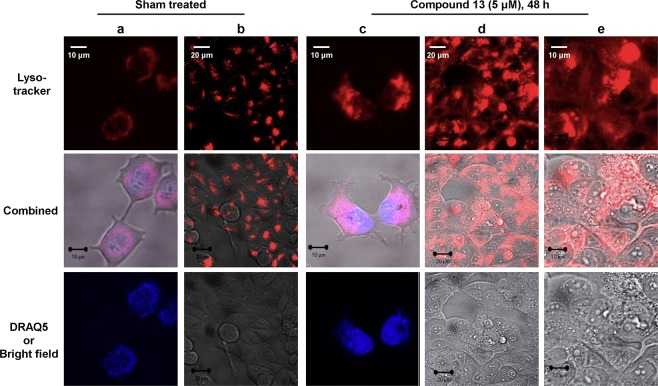
Lysosomal volumes are increased in MCF7 cells treated with compound **13**. Lysomes were visualized by treating MCF7 cells with lysoTracker Red for 48 h in the absence (Sham) or presence of 5 μM compound **13**. The nuclei of cells in rows **a** and **c** were visualized by staining with DRAQ5. Note that rows **a**, **c** and **e** are enlarged images.

We previously found that CQ causes an increase in lysosomal volume^5^, probably due to the accumulation of protonated CQ in the lysosome^26,27^. Since the structure of compound **13** contains the main scaffold of CQ, we examined whether cells treated with compound **13** also results an increase in the lysosomal volume. As shown in Fig. 7, the treatment of cells with compound **13** resulted in a substantial increase in lysosomal volume. Interestingly, the MCF10A non-cancer cells treated with compound **13** did not show the same degree of increase in lysosomal volume (Supplementary Fig. S5). This different reaction between cancer and non-cancer cells could be, at least in part, why compound **13** is more potent against cancer than non-cancer cells.

### Compound 13 kills cancer cells highly effectively when combined with bortezomib (BTZ) or monastrol

**Figure 8:**
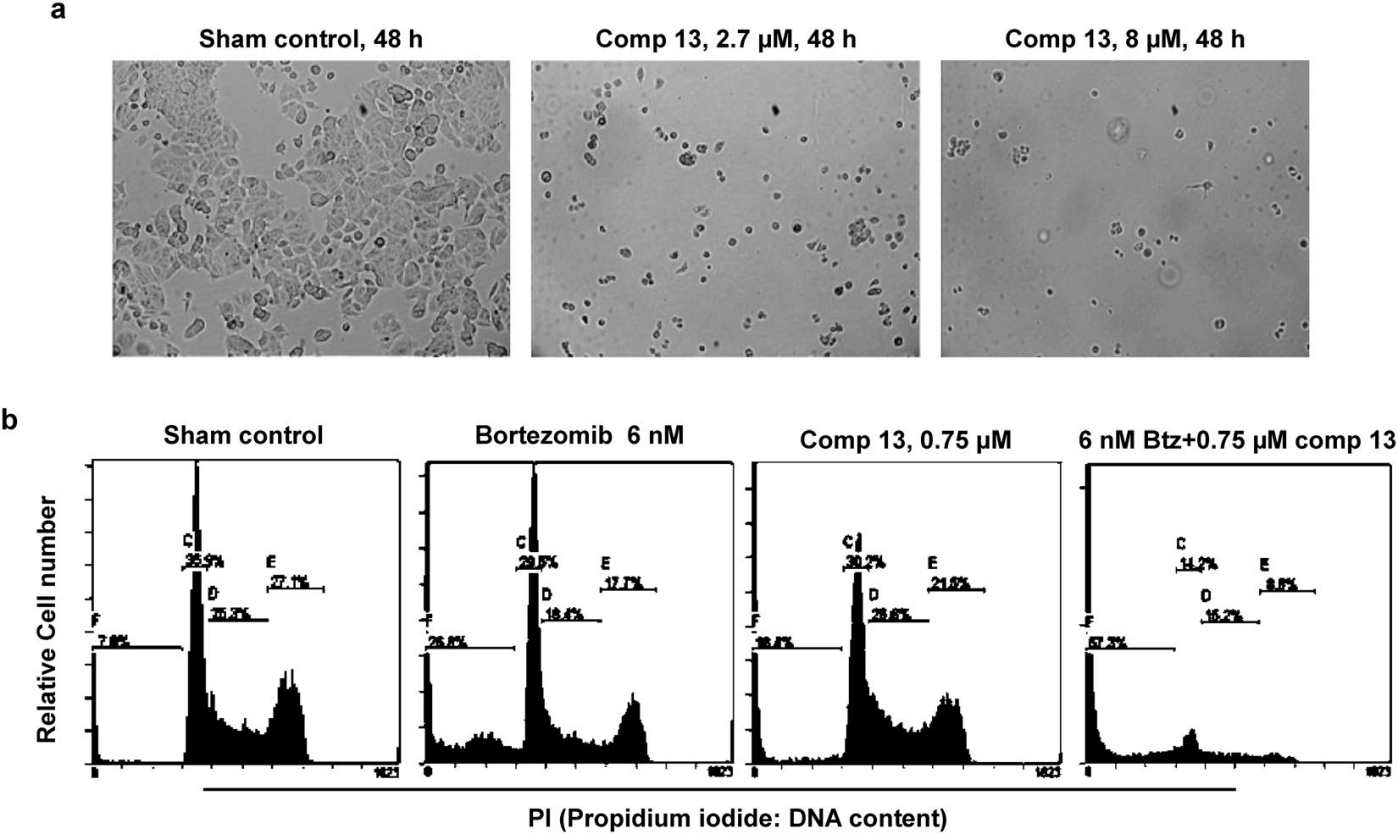
Combination of compound **13** and BTZ is highly effective. (**a**) 2.7–8.0 μM of compound **13** effectively killed cancer cells by 48 h. Asynchronous MCF7 cells were incubated for 48 h in the absence (Sham control) or presence of compound **13** at 2.7 or 8.0 µM. (**b**) The combination of low doses of compound **13** (0.75 μM) and BTZ (6 nM) shows synergistic effects on Jurkat cells. Asynchronously growing Jurkat lymphoma cells were incubated for 48 h in the absence (sham control) or presence of BTZ ± VR23, followed by cell analysis by cytometry.

Compound **13** at 2.7 μM effectively killed MCF7 cells by 48 h of treatment (Fig. 8a). Furthermore, compound **13** showed highly synergistic effects when combined with BTZ or monastrol (Fig. 8b; Supplementary Fig. S6). For example, neither 6 nM BTZ nor 0.75 µM of compound **13** resulted in substantial HeLa cell death by 48 h post-treatment. However, the combination of 6 nM BTZ and 0.75 μM of compound **13** essentially wiped out the entire cell population by 48 h post-treatment, with sub-G1 DNA content typically shown with cells undergoing apoptosis. Similarly, the sequential treatment of monastrol and compound **13** wiped out the entire HeLa cell population within 6 h of the treatment (Supplementary Fig. S6).

## Conclusion

We here report the examination of thirty-six 4-aminoquinoline derived sulfonyl analogs designed and synthesized by a pharmacophore hybrid approach. Most of the hybrid compounds exhibited improved anticancer activity against human breast cancer cells. We found that compounds **8**, **13**, **14**, **16**, **21**, **22**, 2**6**, **35**, **40** and **42** are promising, as they showed stronger antigrowth/antiproliferation activity against cancer cells than non-cancer cells. Among them, 7-chloro-4-(4-(2,4-dinitrophenylsulfonyl)piperazin-1-yl)quinoline (**13**; VR23^28^) is especially desirable as it is not only the most potent one among this series but also shows preferential growth inhibition/cell-killing against cancer over non-cancer cells. For example, up to 17.6-fold higher concentration of compound **13** is required to achieve the same GI_50_ value against non-cancer cells when compared to cancer cells (0.7 μM for MDA-MB468 *vs* 12.3 μM for MCF10A in Table 1). Furthermore, compound **13** is effective on many different types of cancers (Supplementary Fig. S2).

Our data show that compound **13** causes cell cycle arrest at the prometa-metaphase cell cycle position due to the inactivation of Cdk1 through the down-regulation of Cdc25C activity and upregulation of wee1 (Figs 4, 5 and Supplementary Fig. S3), which is likely caused by the formation of multiple centrosomes in response to compound **13** (Fig. 6; Supplementary Fig. S4). As a result, cells eventually die with sub-G1 DNA content typically shown with apoptotic cells (Fig. 8; Supplementary Fig. S6). Compound **13** shows highly synergistic effects when combined with BTZ or monastrol (Fig. 8b; Supplementary Fig. S6).

Like its parental CQ, compound **13** causes an increase in lysomal volume in cancer cells (Fig. 7). We previously found that CQ-mediated increase in lysosomal volumes makes cells vulnerable to anticancer therapies such as radiation^5,6^. Since compound **13**-mediated increase in lysosomal volumes is more cancer cell specific (Fig. 7: Supplementary Fig. S5), the differential effects on cancer and non-cancer cells may contribute at least in part to the preferential cancer cell-killing effect by compound **13**. Overall, our data presented here demonstrates that the hybrid pharmacophore-based approach is very useful in developing effective and potentially safe anticancer agents, and compound **13** possesses a highly desirable property as potential anticancer agent.

## Materials and Methods

Melting points (mp) were taken in open capillaries on the Complab melting point apparatus. Elemental analysis was performed on a Perkin-Elmer 2400 C, H, N analyzer and values were within the acceptable limits of the calculated values. The ^1^H spectra were recorded on a DPX-500 MHz Bruker FT-NMR spectrometer using CDCl_3_ and DMSO-*d*_6_ as solvent. The chemical shifts were reported as parts per million (δ ppm) tetramethylsilane (TMS) as an internal standard. Mass spectra were obtained on a JEOL-SX-102 instrument using electron spray mass spectroscopy (ES-MS). The progress of the reaction was monitored on readymade silica-gel plates (Merck) using chloroform-methanol (9:1) as solvent. Iodine was used as a developing agent or by spraying with the Dragendorff’s reagent. Chromatographic purification was performed over a silica gel (100–200 mesh). All chemicals and reagents obtained from Aldrich (USA) were used without further purification.

### General synthesis of 7-substituted-4-piperazin-1-yl-quinoline (3–4)

A mixture of 7-substituted-4-chloro-quinoline (10.10 mmol), piperazine (2.61 g, 30.30 mmol) or propane-1,3-diamine (30.30 mmol) and triethylamine (1.4 mL, 10.10 mmol) were heated slowly to 80 °C > 1 h while stirring. The temperature was then increased to 130–140 °C for 6 h where it was kept while stirring continuously. The reaction mixture was cooled to room temperature and taken up in dichloromethane. The organic layer was washed with 5% aq. NaHCO_3_, followed by washing with water and then with brine. The organic layer was dried over anhydrous Na_2_SO_4_ and solvent was removed under reduced pressure, and the residue was then precipitated by addition of mixture of solvent hexane: chloroform (8:2).

### 7-Chloro-4-piperazin-1-yl-quinoline (3)

^1^H NMR (500 MHz, CDCl_3_): δ 2.31 (br s, 1 H, N*H*), 3.15 (s, 4 H, N(C*H*_2_CH_2_)_2_NAr), 3.18 (s, 4 H, N(CH_2_C*H*_2_)_2_NAr), 6.80–6.81 (d, *J* = 5.0 Hz, 1 H, Ar-*H*), 7.46–7.47 (d, *J* = 5.0 Hz, 1 H, Ar-*H*), 7.92–7.94 (d, *J* = 10.0 Hz, 1 H, Ar-*H*), 8.01 (s, 1 H, Ar-*H*), 8.68–8.69 (d, *J* = 5.0 Hz, 1 H, Ar-*H*); ^13^C NMR (125 MHz, CDCl_3_): δ 46.10 (2 C), 53.58 (2 C), 108.97, 121.97, 125.24, 126.09, 128.91, 134.84, 150.22, 151.99, 157.38; ES-MS *m/z* 248 [M + H]^+^; Anal.Calcd for C_13_H_14_ClN_3_: C, 63.03; H, 5.70; N, 16.96; found: C, 63.01; H, 5.73; N, 16.99.

### 4-Piperazin-1-yl-7-trifluoromethyl-quinoline (4)

^1^H NMR (500 MHz, CDCl_3_): δ 1.78 (br s, 1 H, N*H*), 3.18 (s, 4 H, N(C*H*_2_CH_2_)_2_NAr), 3.24 (s, 4 H, N(CH_2_C*H*_2_)_2_NAr), 7.07–7.08 (d, *J* = 5.0 Hz, 1 H, Ar-*H*), 7.46–7.47 (d, *J* = 5.0 Hz, 1 H, Ar-*H*), 7.63–7.65 (d, *J* = 10.0 Hz, 1 H, Ar-*H*), 8.13–8.14 (d, *J* = 5.0 Hz, 1 H, Ar-*H*), 8.34 (s, 1 H, Ar-*H*), 8.81–8.82 (d, *J* = 5.0 Hz, 1 H, Ar-*H*); ^13^C NMR (125 MHz, CDCl_3_): δ 52.15 (2 C), 53.48 (2 C), 110.64, 120.79, 125.14, 125.22, 127.76, 130.70, 130.96, 148.73, 152.20, 157.19; ES-MS *m/z* 282 [M + H]^+^; Anal.Calcd for C_14_H_14_F_3_N_3_: C, 59.78; H, 5.02; N, 14.94; found: C, 59.75; H, 4.98; N, 14.97.

### *N*^1^-(7-Chloroquinolin-4-yl)-propane-1,3-diamine (5)

Yellowish white solid; 88% yield; mp 96–98 °C; IR (KBr, cm^−1^): 3328.7; 2236.7; 1587.5; 1216.4_;_^1^H NMR (500 MHz, CDCl_3_): δ 1.89–1.92 (m, 2 H, C*H*_2_), 2.73 (br s, 2 H, N*H*_2_ D_2_O-exchangeable), 3.02–3.06 (m, 2 H, C*H*_2_), 3.35–3.42 (m, 2 H, C*H*_2_), 6.29–6.30 (d, *J* = 5.00 Hz, 1 H, 3 *H* quinoline), 7.28–7.30 (d, *J* = 10.0 Hz, 1 H, 6 *H* quinoline), 7.45 (br s, 1 H, N*H* D_2_O-exchangeable), 7.70–7.72 (d, *J* = 10.0 Hz, 1 H, 5 *H* quinoline), 7.91–7.92 (d, *J* = 5.0 Hz, 1 H, 8 *H* quinoline), 8.47–8.48 (d, *J* = 5.0 Hz, 1 H, 2 *H* quinoline); ^13^C NMR (125 MHz, CDCl_3_): δ 29.81, 37.97, 40.06, 97.35, 116.56, 122.73, 123.01, 126.58, 132.70, 148.09, 149.35, 150.78; FAB-MS *m/z* 236 [M + H]^+^; Anal.Calcd for C_12_H_14_ClN_3_: C, 61.15; H, 5.99; N, 17.83; found: C, 61.15; H, 6.00; N, 17.91.

### *N*^1^-(7-Trifluoromethyl-quinolin-4-yl)-propane-1,3-diamine (6)

White solid; 86% yield; mp 108–110 °C; IR (KBr, cm^−1^): 3332.9; 2231.9; 1584.5; 1212.4_;_^1^H NMR (500 MHz, CDCl_3_): δ 1.74 (br s, 2 H, N*H*_2_ D_2_O-exchangeable), 1.92–1.94 (m, 2 H, CH_2_), 3.02–3.06 (m, 2 H, CH_2_), 3.40–3.44 (m, 2 H, CH_2_), 6.53–6.54 (d, *J* = 5.0 Hz, 1 H, Ar-*H*), 7.55–7.57 (d, *J* = 10.0 Hz, 1 H, Ar-*H*), 7.68 (br s, 1 H, N*H* D_2_O-exchangeable), 7.92–7.93 (d, *J* = 5.0 Hz, 1 H, Ar-*H*), 8.29 (s, 1 H, Ar-*H*), 8.63–8.64 (d, *J* = 10.0 Hz, 1 H, Ar-*H*); ^13^C NMR (125 MHz, CDCl_3_): δ 29.75, 41.56, 43.89, 99.22, 119.75, 120.86, 121.82, 123.03, 125.20, 127.38, 147.70, 150.31, 152.34; ES-MS *m/z* 270 [M + H]^+^; Anal.Calcd for C_13_H_14_F_3_N_3_:C, 57.99; H, 5.24; N, 15.61; found: C, 58.01; H, 5.22; N, 15.65.

### General synthesis of 7-substituted-4-(4-(alkyl/aryl/heteroalkylsulfonyl)piperazin-1-yl)quinolone (7–24)

A solution of compound 7-substituted-4-piperazin-1-yl-quinoline (3.20 mmol) in anhydrous THF (25 mL) under a nitrogen atmosphere was added triethylamine (0.44 mL, 3.20 mmol). The mixture was cooled to below 0 °C. Alkyl/aryl/heteroalkyl sulfonyl chloride (3.20 mmol) was added slowly, keeping the temperature below 5 °C, and the reaction was stirred in an ice bath 1 h. After dilution with saturated NaHCO_3_ solution (20 mL), the reaction was extracted with ether (2X). The organic extracts were dried over Na_2_SO_4_, filtered and evaporated to leave crude compound. The crude product was purified through chromatographed on silica gel, eluting with chloroform/methanol (10/0 to 9/1).

### 7-Chloro-4-(4-(methylsulfonyl)piperazin-1-yl)quinoline (7)

White solid; 72% yield; IR (KBr, cm^−1^): 1167.5 (SO_2_); H NMR (500 MHz, CDCl_3_): δ 2.89 (s, 3 H, SO_2_C*H*_3_), 3.32 (s, 4 H, N(C*H*_2_CH_2_)_2_N-Ar), 3.54 (s, 4 H, N(CH_2_C*H*_2_)_2_N-Ar), 6.88–6.89 (d, *J* = 5.0 Hz, 1 H, Ar-*H*), 7.47–7.49 (d, *J* = 10.0 Hz, 1 H, Ar-*H*), 7.91–7.92 (d, *J* = 5.0 Hz, 1 H, Ar-*H*), 8.07 (s, 1 H, Ar-*H*), 8.79–8.80 (d, *J* = 5.0 Hz, 1 H, Ar-*H*); ^13^C NMR (125 MHz, CDCl_3_): δ 30.32, 45.85 (2 C), 51.77 (2 C), 109.60, 121.77, 124.96, 126.71, 128.74, 135.20, 150.15, 152.01, 156.09; ES-MS *m/z* 327 [M + H]^+^; Anal.Calcd for C_14_H_16_ClN_3_O_2_S: C, 51.61; H, 4.95; N, 12.90; found: C, 51.59; H, 4.97; N, 12.87.

### 7-Chloro-4-(4-tosylpiperazin-1-yl)quinoline (8)

Pale yellowish white solid; 66% yield; mp 190–192 °C; IR (KBr, cm^−1^): 1168.7 (SO_2_); ^1^H NMR (500 MHz, CDCl_3_): δ 2.51 (s, 3 H, CH_3_), 3.37 (s, 8 H, N(C*H*_2_C*H*_2_)_2_N), 6.86–6.87 (d, *J* = 5.0 Hz, 1 H, Ar-*H*), 7.39–7.42 (m, 3 H, Ar-*H*), 7.74–7.79 (m, 3 H, Ar-*H*), 8.06 (s, 1 H, Ar-*H*), 8.75–8.76 (d, *J* = 5.0 Hz, 1 H, Ar-*H*); ES-MS *m/z* 403 [M + H]^+^; Anal.Calcd for C_20_H_20_ClN_3_O_2_S: C, 59.77; H, 5.02; N, 10.46; found: C, 59.74; H, 5.06; N, 10.42.

### 7-Chloro-4-(4-(biphenylsulfonyl)piperazin-1-yl)quinoline (9)

Plae yellow solid; 68% yield; mp 238–240 °C; IR (KBr, cm^−1^): 1165.3 (SO_2_); ^1^H NMR (500 MHz, CDCl_3_): δ 3.30 (s, 4 H, N(CH_2_C*H*_2_)_2_N-Ar), 3.39 (s, 4 H, N(C*H*_2_CH_2_)_2_N-Ar), 6.95–6.96 (d, *J* = 5.0 Hz, 1 H, Ar-*H*), 7.42–7.44 (d, *J* = 10.0 Hz, 1 H, Ar-*H*), 7.46–7.50 (m, 2 H, Ar-*H*), 7.55–7.57 (d, *J* = 10.0 Hz, 2 H, Ar-*H*), 7.61–7.63 (d, *J* = 10.0 Hz, 2 H, Ar-*H*), 7.68–7.70 (d, *J* = 10.0 Hz, 2 H, Ar-*H*), 7.98–8.00 (d, *J* = 10.0 Hz, 2 H, Ar-*H*), 8.35 (s, 1 H, Ar-*H*), 8.84–8.85 (d, *J* = 5.0 Hz, 1 H, Ar-*H*); ^13^C NMR (125 MHz, CDCl_3_): δ 43.52, 46.07, 51.57 (2 C), 109.50, 121.69, 124.64, 126.55, 127.34, 127.88, 128.37, 128.67, 129.30, 130.89, 132.64, 134.07, 135.09, 139.06, 146.09, 150.09, 151.99,156.06; ES-MS *m/z* 465 [M + H]^+^; Anal.Calcd for C_25_H_22_ClN_3_O_2_S: C, 64.72; H, 4.78; N, 9.06; found: C, 64.76; H, 4.80; N, 9.04.

### 7-Chloro-4-(4-(4-chlorophenylsulfonyl)piperazin-1-yl)quinoline (10)

Pale yellowish white solid; 73% yield; IR (KBr, cm^−1^): 1170.7 (SO_2_); ^1^H NMR (500 MHz, CDCl_3_): δ 3.31 (s, 4 H, N(CH_2_C*H*_2_)_2_N-Ar), 3.35 (s, 4 H, N(C*H*_2_CH_2_)_2_N-Ar), 6.87–6.88 (d, *J* = 5.0 Hz, 1 H, Ar-*H*), 7.23 (s, 1 H, Ar-*H*), 7.40–7.44 (dd, *J*_1_ = 10.0 Hz, *J*_2_ = 5.0 Hz, 2 H, Ar-*H*), 7.61–7.62 (d, *J* = 5.0 Hz, 1 H, Ar-*H*), 7.78–7.82 (dd, *J*_1_ = 10.0 Hz, *J*_2_ = 5.0 Hz, 2 H, Ar-*H*), 8.07 (s, 1 H, Ar-*H*), 8.77–8.78 (d, *J* = 5.0 Hz, 1 H, Ar-*H*); ^13^C NMR (125 MHz, CDCl_3_): δ 46.02 (2 C), 51.95 (2 C), 109.52, 121.69, 124.49, 126.67 (2 C), 129.18, 129.22, 129.68 (2 C), 134.14, 135.22, 139.91, 150.16, 151.99, 155.98; ES-MS *m/z* 423 [M + H]^+^; Anal.Calcd for C_19_H_17_Cl_2_N_3_O_2_S: C, 54.03; H, 4.06; N, 9.95; found: C, 54.06; H, 4.04; N, 9.97.

### 7-Chloro-4-(4-(2,4-dichlorophenylsulfonyl)piperazin-1-yl)quinoline (11)

Pale yellowish white solid; 68% yield; mp 145–147 °C; IR (KBr, cm^−1^): 1172.3 (SO_2_); ^1^H NMR (500 MHz, CDCl_3_): δ 3.24 (s, 4 H, N(CH_2_C*H*_2_)_2_N-Ar), 3.63 (s, 4 H, N(C*H*_2_CH_2_)_2_N-Ar), 6.86–6.87 (d, *J* = 5.0 Hz, 1 H, Ar-*H*), 7.43–7.45 (m, 2 H, Ar-*H*), 7.54–7.55 (d, *J* = 5.0 Hz, 1 H, Ar-*H*), 7.61 (s, 1 H, Ar-*H*), 7.87–7.89 (d, *J* = 10.0 Hz, 1 H, Ar-*H*), 8.05 (s, 1 H, Ar-*H*), 8.76–8.77 (d, *J* = 5.0 Hz, 1 H, Ar-*H*); ^13^C NMR (125 MHz, CDCl_3_): δ 41.00 (2 C), 47.26 (2 C), 104.81, 117.02, 119.81, 121.96, 122.75, 124.38, 127.38, 128.34, 128.59, 129.86, 130.47, 135.13, 145.40, 147.22, 151.40; ES-MS *m/z* 458 [M + H]^+^; Anal.Calcd for C_19_H_16_Cl_3_N_3_O_2_S: C, 49.96; H, 3.53; N, 9.20; found: C, 49.99; H, 3.51; N, 9.18.

### 7-Chloro-4-(4-(3-nitrophenylsulfonyl)piperazin-1-yl)quinoline (12)

Yellow solid; 73% yield; mp 138–140 °C; IR (KBr, cm^−1^): 1174.7 (SO_2_); ^1^H NMR (500 MHz, CDCl_3_): δ 3.33 (s, 4 H, N(CH_2_C*H*_2_)_2_N-Ar), 3.42 (s, 4 H, N(C*H*_2_CH_2_)_2_N-Ar), 6.87–6.88 (d, *J* = 5.0 Hz, 1 H, Ar-*H*), 7.40–7.43 (dd, *J*_1_ = 10.0 Hz, *J*_2_ = 5.0 Hz, 1 H, Ar-*H*), 7.76–7.68 (d, *J* = 10.0 Hz, 1 H, Ar-*H*), 7.86–7.89 (dd, *J*_1_ = 10.0 Hz, *J*_2_ = 5.0 Hz, 1 H, Ar-*H*), 8.10 (s, 1 H, Ar-*H*), 8.19–8.20 (d, *J* = 5.0 Hz, 1 H, Ar-*H*), 8.52–8.53 (dd, *J*_1_ = 10.0 Hz, *J*_2_ = 5.0 Hz, 1 H, Ar-*H*), 8.70 (s, 1 H, Ar-*H*), 8.81–82 (d, *J* = 5.0 Hz, 1 H, Ar-*H*); ^13^C NMR (125 MHz, CDCl_3_): δ 46.09 (2 C), 52.22 (2 C), 109.57, 121.63, 122.83, 124.38, 126.75, 127.67, 129.20, 130.79, 133.19, 135.27, 138.20, 148.57, 150.14, 151.99, 155.81; ES-MS *m/z* 434 [M + H]^+^; Anal.Calcd for C_19_H_17_ClN_4_O_4_S: C, 52.72; H, 3.96; N, 12.94; found: C, 52.70; H, 3.99; N, 12.92.

### 7-Chloro-4-(4-(2,4-dinitrophenylsulfonyl)piperazin-1-yl)quinoline (13)

Yellow solid; 68% yield; mp 238–240 °C; IR (KBr, cm^−1^): 1174.3 (SO_2_); ^1^H NMR (500 MHz, CDCl_3_): δ 3.33 (s, 4 H, N(CH_2_C*H*_2_)_2_N-Ar), 3.69 (s, 4 H, N(C*H*_2_CH_2_)_2_N-Ar), 6.89–6.90 (d, *J* = 5.0 Hz, 1 H, Ar-*H*), 7.46–7.48 (d, *J* = 10.0 Hz, 1 H, Ar-*H*), 7.87–7.89 (d, *J* = 10.0 Hz, 1 H, Ar-*H*), 8.09–8.10 (d, *J* = 5.0 Hz, 1 H, Ar-*H*), 8.31–8.33 (d, *J* = 10.0 Hz, 1 H, Ar-*H*), 8.55 (s, 1 H, Ar-*H*), 8.57–8.59 (d, *J* = 5.0 Hz, 1 H, Ar-*H*), 8.78–8.79 (d, *J* = 5.0 Hz, 1 H, Ar-*H*); ^13^C NMR (125 MHz, CDCl_3_): δ 46.19 (2 C), 51.93 (2 C), 109.67, 119.91, 121.71, 124.36, 126.20, 126.89, 129.24, 132.78, 135.36, 137.10, 140.51, 150.18, 151.99, 155.85, 159.75; ES-MS *m/z* 479 [M + H]^+^; Anal.Calcd for C_19_H_16_ClN_5_O_6_S: C, 47.75; H, 3.37; N, 14.66; found: C, 47.77; H, 3.39; N, 14.63.

### 5-(4-(7-Chloroquinolin-4-yl)piperazin-1-ylsulfonyl)-N,N-dimethylnaphthalen-1-amine (14)

Pale yellowish white solid; 68% yield; mp 145–147 °C; IR (KBr, cm^−1^): 3295.7 (NH); 1192.3 (SO_2_); ^1^H NMR (500 MHz, CDCl_3_): δ 2.94 (s, 6 H, N(C*H*_3_)_2_), 3.25 (s, 4 H, N(CH_2_C*H*_2_)_2_N-Ar), 3.52 (s, 4 H, N(C*H*_2_CH_2_)_2_N-Ar), 6.80–6.81 (d, *J* = 5.0 Hz, 1 H, Ar-*H*), 7.22–7.23 (d, *J* = 5.0 Hz, 1 H, Ar-*H*), 7.28–7.29 (d, *J* = 5.0 Hz, 1 H, Ar-*H*), 7.58–7.60 (d, *J* = 10.0 Hz, 1 H, Ar-*H*), 7.78–7.79 (d, *J* = 5.0 Hz, 1 H, Ar-*H*), 7.80–7.81 (d, *J* = 5.0 Hz, 1 H, Ar-*H*), 8.29–8.31 (dd, *J*_1_ = 10.0 Hz, *J*_2_ = 5.0 Hz, 1 H, Ar-*H*), 8.46–8.48 (dd, *J*_1_ = 10.0 Hz, *J*_2_ = 5.0 Hz, 1 H, Ar-*H*), 8.63–8.65 (dd, *J*_1_ = 10.0 Hz, *J*_2_ = 5.0 Hz, 1 H, Ar-*H*), 8.71 (s, 1 H, Ar-*H*), 8.72–8.73 (d, *J* = 5.0 Hz, 1 H, Ar-*H*); ^13^C NMR (125 MHz, CDCl_3_): δ 45.45 (2 C), 45.54 (2 C), 51.79 (2 C), 109.44, 115.37, 119.53, 121.72, 123.24, 124.64, 126.58, 128.26, 128.26, 129.05, 130.16, 130.47, 130.88, 131.05, 132.53, 135.14, 150.10, 151.92, 156.19; ES-MS *m/z* 482 [M + H]^+^; Anal.Calcd for C_25_H_25_ClN_4_O_2_S: C, 62.42; H, 5.24; N, 11.65; found: C, 62.44; H, 5.21; N, 11.61.

### Methyl 3-(4-(7-chloroquinolin-4-yl)piperazin-1-ylsulfonyl)thiophene-2-carboxylate (15)

Pale yellowish white solid; 72% yield; mp 117–119 °C; IR (KBr, cm^−1^): 1169.8 (SO_2_); ^1^H NMR (500 MHz, CDCl_3_): δ 3.44 (s, 4 H, N(C*H*_2_CH_2_)_2_N-Ar), 3.65 (s, 4 H, N(CH_2_C*H*_2_)_2_N-Ar), 3.91 (s, 3 H, COOC*H*_3_), 6.85–6.86 (d, *J* = 5.0 Hz, 1 H, Ar-*H*), 7.41–7.42 (d, *J* = 5.0 Hz, 1 H, Ar-*H*), 7.54–7.57 (dd, *J*_1_ = 10.0 Hz, *J*_2_ = 5.0 Hz, 1 H, Ar-*H*), 7.72–7.73 (d, *J* = 5.0 Hz, 1 H, Ar-*H*), 7.86–7.88 (d, *J* = 10.0 Hz, 1 H, Ar-*H*), 8.06 (s, 1 H, Ar-*H*), 8.74–8.75 (d, *J* = 5.0 Hz, 1 H, Ar-*H*); ^13^C NMR (125 MHz, CDCl_3_): δ 46.13 (2 C), 52.02 (2 C), 109.48, 121.77, 124.69, 126.59, 128.80, 129.31, 130.89, 132.45, 134.07, 135.14, 140.44, 151.97, 156.27, 159.95, 167.75; ES-MS *m/z* 453 [M + H]^+^; Anal.Calcd for C_19_H_18_ClN_3_O_4_S_2_: C, 50.49; H, 4.01; N, 9.30; found: C, 50.51; H, 4.04; N, 9.34.

### 4-(4-Methanesulfonyl-piperazin-1-yl)-7-trifluoromethyl-quinoline (16)

Pale yellowish white solid; 70% yield; mp 116–118 °C; IR (KBr, cm^−1^): 1170.5 (SO_2_); ^1^H NMR (500 MHz, CDCl_3_): δ 2.91 (s, 3 H, SO_2_C*H*_3_), 3.38 (s, 4 H, N(C*H*_2_CH_2_)_2_N-Ar), 3.59 (s, 4 H, N(CH_2_C*H*_2_)_2_N-Ar), 7.01–7.02 (d, *J* = 5.0 Hz, 1 H, Ar-*H*), 7.70–7.71 (d, *J* = 5.0 Hz, 1 H, Ar-*H*), 8.11–8.13 (d, *J* = 10.0 Hz, 1 H, Ar-*H*), 8.41 (s, 1 H, Ar-*H*), 8.88–8.89 (d, *J* = 5.0 Hz, 1 H, Ar-*H*); ^13^C NMR (125 MHz, CDCl_3_): δ 34.94, 45.83 (2 C), 51.79 (2 C), 110.90, 121.40, 124.95, 125.04, 128.11, 131.06, 131.32, 148.79, 152.27, 155.94; ES-MS *m/z* 360 [M + H]^+^; Anal.Calcd for C_15_H_16_F_3_N_3_O_2_S: C, 50.13; H, 4.49; N, 11.69; found: C, 50.15; H, 4.51; N, 11.66.

### 4-[4-(Toluene-4-sulfonyl)-piperazin-1-yl]-7-trifluoromethyl-quinoline (17)

Creamy white solid; 74% yield; mp 126–128 °C; IR (KBr, cm^−1^): 1165.2 (SO_2_); ^1^H NMR (500 MHz, CDCl_3_): δ 2.49 (s, 3 H, CH_3_), 3.35 (s, 8 H, N(C*H*_2_C*H*_2_)_2_N), 6.96–6.97 (d, *J* = 5.0 Hz, 1 H, Ar-*H*), 7.42–7.43 (d, *J* = 10.0 Hz, 1 H, Ar-*H*), 7.61–7.62 (d, *J* = 5.0 Hz, 1 H, Ar-*H*), 7.74–7.76 (d, *J* = 10.0 Hz, 2 H, Ar-*H*), 7.96–7.98 (d, *J* = 10.0 Hz, 2 H, Ar-*H*), 8.37 (s, 1 H, Ar-*H*), 8.84–8.85 (d, *J* = 5.0 Hz, 1 H, Ar-*H*); ^13^C NMR (125 MHz, CDCl_3_): δ 21.62, 46.00 (2 C), 51.65 (2 C), 110.74, 121.23, 124.53, 124.95, 127.89, 127.98, 128.02 (2 C), 129.95 (2 C), 131.22, 132.48, 144.17, 148.68, 152.21, 155.95; ES-MS *m/z* 436 [M + H]^+^; Anal.Calcd for C_21_H_20_F_3_N_3_O_2_S: C, 57.92; H, 4.63; N, 9.65; found: C, 57.89; H, 4.60; N, 9.62.

### 4-[4-(Biphenyl-4-sulfonyl)-piperazin-1-yl]-7-trifluoromethyl-quinoline (18)

White solid; 76% yield; mp 148–150 °C; IR (KBr, cm^−1^) 1165.3 (SO_2_); ^1^H NMR (500 MHz, CDCl_3_): δ 3.37 (s, 4 H, N(CH_2_C*H*_2_)_2_N-Ar), 3.43 (s, 4 H, N(C*H*_2_CH_2_)_2_N-Ar), 6.97–6.98 (d, *J* = 5.0 Hz, 1 H, Ar-*H*), 7.45–7.47 (d, *J* = 10.0 Hz, 1 H, Ar-*H*), 7.48–7.52 (m, 2 H, Ar-*H*), 7.53–7.55 (d, *J* = 10.0 Hz, 2 H, Ar-*H*), 7.61–7.63 (d, *J* = 10.0 Hz, 2 H, Ar-*H*), 7.66–7.68 (d, *J* = 10.0 Hz, 2 H, Ar-*H*), 7.98–8.00 (d, *J* = 10.0 Hz, 2 H, Ar-*H*), 8.37 (s, 1 H, Ar-*H*), 8.85–8.86 (d, *J* = 5.0 Hz, 1 H, Ar-*H*); ^13^C NMR (125 MHz, CDCl_3_): δ 45.99, 46.05, 51.52, 51.59, 110.78, 121.27, 124.53, 124.96, 127.35, 127.91, 128.05 (2 C), 128.38 (2 C), 128.72 (2 C), 129.17 (2 C), 129.30, 130.97, 134.06, 139.08, 146.18, 148.73, 152.24, 155.91; ES-MS *m/z* 499 [M + H]^+^; Anal.Calcd for C_26_H_22_F_3_N_3_O_2_S: C, 62.77; H, 4.46; N, 8.45; found: C, 62.77; H, 4.46; N, 8.45.

### 4-[4-(4-Chloro-benzenesulfonyl)-piperazin-1-yl]-7-trifluoromethyl-quinoline (19)

White solid; 69% yield; mp 144–146 °C; IR (KBr, cm^−1^): 1174.9 (SO_2_); mp 171–173 °C; ^1^H NMR (500 MHz, CDCl_3_): δ 3.23 (s, 4 H, N(CH_2_C*H*_2_)_2_N-Ar), 3.57 (s, 4 H, N(C*H*_2_CH_2_)_2_N-Ar), 7.01–7.02 (d, *J* = 5.0 Hz, 1 H, Ar-*H*), 7.64–7.66 (d, *J* = 10.0 Hz, 2 H, Ar-*H*), 7.72–7.73 (d, *J* = 5.0 Hz, 1 H, Ar-*H*), 7.85–7.87 (d, *J* = 10.0 Hz, 2 H, Ar-*H*), 7.97–7.99 (d, *J* = 10.0 Hz, 1 H, Ar-*H*), 8.47 (s, 1 H, Ar-*H*), 8.84–8.85 (d, *J* = 5.0 Hz, 1 H, Ar-*H*); ^13^C NMR (125 MHz, CDCl_3_): δ 45.91 (2 C), 51.52 (2 C), 110.47, 122.55, 124.47, 124.73, 126.89, 127.54, 129.20 (2 C), 129.73 (2 C), 131.84, 134.03, 140.01, 147.36, 151.09, 156.58; ES-MS *m/z* 457 [M + H]^+^; Anal.Calcd for C_20_H_17_ClF_3_N_3_O_2_S: C, 52.69; H, 3.76; N, 9.22; found: C, 52.71; H, 3.74; N, 9.20.

### 4-[4-(2,4-Dichloro-benzenesulfonyl)-piperazin-1-yl]-7-trifluoromethyl-quinoline (20)

White solid; 70% yield; mp 137–139 °C; IR (KBr, cm^−1^): 1169.8 (SO_2_); ^1^H NMR (500 MHz, CDCl_3_): δ 3.29 (s, 4 H, N(CH_2_C*H*_2_)_2_N-Ar), 3.63 (s, 4 H, N(C*H*_2_CH_2_)_2_N-Ar), 6.97–6.98 (d, *J* = 5.0 Hz, 1 H, Ar-*H*), 7.44–7.46 (d, *J* = 5.0 Hz, 1 H, Ar-*H*), 7.61 (s, 1 H, Ar-*H*), 7.66–7.68 (d, *J* = 10.0 Hz, 1 H, Ar-*H*), 8.08–8.10 (d, *J* = 10.0 Hz, 2 H, Ar-*H*), 8.38 (s, 1 H, Ar-*H*), 8.85–8.86 (d, *J* = 5.0 Hz, 1 H, Ar-*H*); ^13^C NMR (125 MHz, CDCl_3_): δ 45.72 (2 C), 52.00 (2 C), 110.86, 120.60, 121.39, 124.50, 124.94, 125.02, 127.11, 128.07, 132.15, 133.09, 133.34, 134.58, 139.93, 148.76, 152.23, 155.99; ES-MS *m/z* 491 [M + H]^+^; Anal.Calcd for C_20_H_16_Cl_2_F_3_N_3_O_2_S: C, 48.99; H, 3.29; N, 8.57; found: C, 49.01; H, 3.31; N, 8.61.

### 4-[4-(3-Nitro-benzenesulfonyl)-piperazin-1-yl]-7-trifluoromethyl-quinoline (21)

Pale yellowish white solid; 65% yield; mp 199–201 °C; IR (KBr, cm^−1^): 1168.9 (SO_2_);^1^H NMR (500 MHz, CDCl_3_): δ 3.36 (s, 4 H, N(CH_2_C*H*_2_)_2_N-Ar), 3.54 (s, 4 H, N(C*H*_2_CH_2_)_2_N-Ar), 6.98–6.99 (d, *J* = 5.0 Hz, 1 H, Ar-*H*), 7.62–7.64 (d, *J* = 10.0 Hz, 1 H, Ar-*H*), 7.86–7.88 (d, *J* = 10.0 Hz, 1 H, Ar-*H*), 7.90–7.91 (d, *J* = 5.0 Hz, 1 H, Ar-*H*), 7.96–7.97 (d, *J* = 5.0 Hz, 1 H, Ar-*H*), 8.20–8.21 (d, *J* = 5.0 Hz, 1 H, Ar-*H*), 8.55–8.57 (d, *J* = 10.0 Hz, 1 H, Ar-*H*), 8.71 (s, 1 H, Ar-*H*), 8.85–8.86 (d, *J* = 5.0 Hz, 1 H, Ar-*H*); ^13^C NMR (125 MHz, CDCl_3_): δ 46.05 (2 C), 51.52 (2 C), 110.87, 121.40, 122.83, 124.32, 124.89, 127.70, 128.14 (2 C), 130.81, 131.08, 133.19, 138.20, 148.58, 148.78, 152.23, 155.65; ES-MS *m/z* 467 [M + H]^+^; Anal.Calcd for C_20_H_17_F_3_N_4_O_4_S: C, 51.50; H, 3.67; N, 12.01; found: C, 51.47; H, 3.70; N, 11.97.

### 4-[4-(2,4-Dinitro-benzenesulfonyl)-piperazin-1-yl]-7-trifluoromethyl-quinoline (22)

Yellow solid; 66% yield; mp 178–180 °C; IR (KBr, cm^−1^): 1160.3 (SO_2_); ^1^H NMR (500 MHz, CDCl_3_): δ 3.36 (s, 4 H, N(CH_2_C*H*_2_)_2_N-Ar), 3.71 (s, 4 H, N(C*H*_2_CH_2_)_2_N-Ar), 6.99–7.00 (d, *J* = 5.0 Hz, 1 H, Ar-*H*), 7.68–7.70 (d, *J* = 10.0 Hz, 1 H, Ar-*H*), 8.06–8.07 (d, *J* = 5.0 Hz, 1 H, Ar-*H*), 8.31–8.33 (d, *J* = 10.0 Hz, 1 H, Ar-*H*), 8.55–8.57 (d, *J* = 10.0 Hz, 1 H, Ar-*H*), 8.57–8.59 (d, *J* = 10.0 Hz, 1 H, Ar-*H*), 8.62 (s, 1 H, Ar-*H*), 8.87–8.88 (d, *J* = 5.0 Hz, 1 H, Ar-*H*); ^13^C NMR (125 MHz, CDCl_3_): δ 46.15 (2 C), 51.91 92 (2 C), 110.97, 119.53, 121.54, 124.31 (2 C), 124.89, 124.96, 126.24, 128.15, 132.78, 137.07, 148.41, 148.77, 150.03, 152.23, 155.70; ES-MS *m/z* 512 [M + H]^+^; Anal.Calcd for C_20_H_16_F_3_N_5_O_6_S: C, 46.97; H, 3.15; N, 13.69; found: C, 46.94; H, 3.17; N, 13.66.

### Dimethyl-{5-[4-(7-trifluoromethyl-quinolin-4-yl)-piperazine-1-sulfonyl]-naphthalen-1-yl}-amine (23)

Pale yellowish white solid; 69% yield; mp 98–100 °C; IR (KBr, cm^−1^): 1170.8 (SO_2_); ^1^H NMR (500 MHz, CDCl_3_): δ 2.91 (s, 6 H, N(C*H*_3_)_2_), 3.28 (s, 4 H, N(CH_2_C*H*_2_)_2_N-Ar), 3.55 (s, 4 H, N(C*H*_2_CH_2_)_2_N-Ar), 6.92–6.93 (d, *J* = 5.0 Hz, 1 H, Ar-*H*), 7.24–7.25 (d, *J* = 5.0 Hz, 1 H, Ar-*H*), 7.60–7.63 (m, 3 H, Ar-*H*), 7.98–8.00 (d, *J* = 10.0 Hz, 1 H, Ar-*H*), 8.30–8.32 (d, *J* = 10.0 Hz, 1 H, Ar-*H*), 8.36 (s, 1 H, Ar-*H*), 8.47–8.48 (d, *J* = 5.0 Hz, 1 H, Ar-*H*), 8.64–8.65 (d, *J* = 5.0 Hz, 1 H, Ar-*H*), 8.82–8.83 (d, *J* = 5.0 Hz, 1 H, Ar-*H*); ^13^C NMR (125 MHz, CDCl_3_): δ 45.45 (2 C), 45.51 (2 C), 51.78 (2 C), 110.72, 115.38, 119.50, 121.27, 123.25, 124.56, 124.93, 124.98, 127.97, 128.28, 130.17, 130.47, 130.90, 131.09, 131.22, 132.51, 148.71, 151.94, 152.18, 156.02; ES-MS *m/z* 516 [M + H]^+^; Anal.Calcd for C_26_H_25_F_3_N_4_O_2_S: C, 60.69; H, 4.90; N, 10.89; found: C, 60.65; H, 4.93; N, 10.87.

### 3-[4-(7-Trifluoromethyl-quinolin-4-yl)-piperazine-1-sulfonyl]-thiophene-2-carboxylic acid methyl ester (24)

White solid; 68% yield; mp 117–119 °C; IR (KBr, cm^−1^): 1165.6 (SO_2_); ^1^H NMR (500 MHz, CDCl_3_): δ 3.32 (s, 4 H, N(C*H*_2_CH_2_)_2_N-Ar), 3.68 (s, 4 H, N(CH_2_C*H*_2_)_2_N-Ar), 3.91 (s, 3 H, COOC*H*_3_), 6.96–6.97 (d, *J* = 5.0 Hz, 1 H, Ar-*H*), 7.54–7.58 (dd, *J*_1_ = 10.0 Hz, *J*_2_ = 5.0 Hz, 2 H, Ar-*H*), 7.64–7.66 (d, *J* = 10.0 Hz, 1 H, Ar-*H*), 8.05–8.07 (d, *J* = 10.0 Hz, 1 H, Ar-*H*), 8.05–8.07 (d, *J* = 10.0 Hz, 1 H, Ar-*H*), 8.37 (s, 1 H, Ar-*H*), 8.83–8.84 (d, *J* = 5.0 Hz, 1 H, Ar-*H*); ^13^C NMR (125 MHz, CDCl_3_): δ 46.09 (2 C), 52.01 (2 C), 53.13, 110.74, 121.25, 122.78, 124.99, 127.90, 129.37, 130.98, 131.24, 131.42, 134.07, 140.42, 148.59, 152.31, 156.01, 159.93; ES-MS *m/z* 487 [M + H]^+^; Anal.Calcd for C_20_H_18_F_3_N_3_O_4_S_2_: C, 49.48; H, 3.74; N, 8.66; found: C, 49.52; H, 3.77; N, 8.68.

### General synthesis of N-[3-(7-Chloro-quinolin-4-ylamino)-propyl]- alkene/aryene/heteroalkene sulfonamide (25–42)

A solution of compound *N*^1^-(7-subtituted-quinolin-4-yl)-popane-1,3-diamine (**5** or **6**) (3.20 mmol) in anhydrous THF (25 mL) under a nitrogen atmosphere triethylamine (0.44 mL, 3.20 mmol) was added. The mixture was cooled to below 0 °C. Alkyl/aryl/heteroalkyl sulfonyl chloride (3.20 mmol) was added slowly, keeping the temperature below 5 °C, and the reaction was stirred in an ice bath 1 h. After dilution with saturated NaHCO_3_ solution (20 mL), the reaction was extracted with ether (2X). The organic extracts were dried over Na_2_SO_4_, filtered and evaporated to leave crude compound. The crude product was purified through chromatographed on silica gel, eluting with chloroform/methanol (10/0 to 8/2).

### *N*-[3-(7-Chloro-quinolin-4-ylamino)-propyl]-methanesulfonamide (25)

White solid; 76% yield; IR (KBr, cm^−1^) 3320.5 (NH); 1185.3 (SO_2_); ^1^H NMR (500 MHz, CDCl_3_): δ 1.88–1.92 (m, 2 H, CH_2_), 2.86 (s, 3 H, SO_2_C*H*_3_), 3.24–3.27 (m, 2 H, CH_2_), 3.42–3.46 (m, 2 H, CH_2_), 6.37–6.38 (d, *J* = 5.0 Hz, 1 H, Ar-*H*), 7.00 (br s, 1 H, N*H*), 7.08 (br s, 1 H, N*H*), 7.27–7.28 (d, *J* = 5.0 Hz, 1 H, Ar-*H*), 7.72–7.74 (d, Hz, *J* = 10.0 Hz, 1 H, Ar-*H*), 8.11–8.13 (d, *J* = 10.0 Hz, 1 H, Ar-*H*), 8.35–8.37 (d, *J* = 5.0 Hz, 1 H, Ar-*H*); ^13^C NMR (125 MHz, CDCl_3_): δ 28.36, 39.64, 39.97, 47.81, 98.75, 117.80, 123.70, 124.42, 127.92, 134.21, 149.32, 150.45, 151.95; ES-MS *m/z* 391 [M + H]^+^; Anal.Calcd for C_13_H_16_ClN_3_O_2_S: C, 49.76; H, 5.14; N, 13.39; found: C, 49.71; H, 5.10; N, 13.41.

### *N*-[3-(7-Chloro-quinolin-4-ylamino)-propyl]-4-methyl-benzenesulfonamide (26)

Creamy white solid; 74% yield; IR (KBr, cm^−1^): 3290.6 (NH); 1175.2 (SO_2_); ^1^H NMR (500 MHz, CDCl_3_): δ 1.87–1.97 (m, 2 H, CH_2_), 2.27 (s, 3 H, CH_3_), 3.06–3.13 (m, 2 H, CH_2_), 3.52–3.56 (m, 2 H, CH_2_), 5.69 (br s, 1 H, N*H* D_2_O-exchangeable), 6.34–6.35 (d, = 5.0 Hz, 1 H, Ar-*H*), 7.31–7.38 (m, 4 H, Ar-*H*), 7.40–7.41 (d, *J* = 5.0 Hz, 1 H, Ar-*H*), 7.74 (br s, 1 H, N*H* D_2_O-exchangeable), 7.76–7.78 (d, *J* = 10.0 Hz, 1 H, Ar-*H*), 7.97–7.99 (d, *J* = 10.0 Hz, 1 H, Ar-*H*), 8.48–8.49 (d, *J* = 5.0 Hz, 1 H, Ar-*H*); ES-MS *m/z* 391 [M + H]^+^; Anal.Calcd for C_19_H_20_ClN_3_O_2_S: C, 58.53; H, 5.17; N, 10.78; found: C, 58.49; H, 5.19; N, 10.81.

### Biphenyl-4-sulfonic acid [3-(7-chloro-quinolin-4-ylamino)-propyl]-amide (27)

Pale yellowish white solid; 70% yield; mp 116–118 °C; IR (KBr, cm^−1^): 3260.9 (NH); 1180.5 (SO_2_); ^1^H NMR (500 MHz, CDCl_3_): δ 1.82–1.85 (m, 2 H, CH_2_), 2.89–2.94 (m, 2 H, CH_2_), 3.48–3.52 (m, 2 H, CH_2_), 6.30–6.31 (d, *J* = 5.0 Hz, 1 H, Ar-*H*), 6.92 (br s, 1 H, N*H* D_2_O exchangeable), 7.28–7.29 (d, *J* = 5.0 Hz, 1 H, Ar-*H*), 7.32–7.35 (d, *J* = 10.0 Hz, 1 H, Ar-*H*), 7.38–7.40 (d, *J* = 10.0 Hz, 1 H, Ar-*H*), 7.49–7.51 (d, *J* = 10.0 Hz, 1 H, Ar-*H*), 7.54 (s, 1 H, Ar-*H*), 7.59–7.61 (d, *J* = 10.0 Hz, 2 H, Ar-*H*), 7.63–7.65 (d, *J* = 10.0 Hz, 2 H, Ar-*H*), 7.70 (s, 1 H, Ar-*H*), 7.77 (br s, 1 H, N*H* D_2_O exchangeable), 7.82–7.84 (d, *J* = 10.0 Hz, 1 H, Ar-*H*), 8.02–8.04 (d, *J* = 10.0 Hz, 1 H, Ar-*H*), 8.30–8.31 (d, *J* = 5.0 Hz, 1 H, Ar-*H*); ^13^C NMR (125 MHz, CDCl_3_): δ 22.55, 27.69, 40.62, 98.57, 117.42, 123.29, 124.93 (2 C), 127.04 (2 C), 127.17 (2 C), 127.37 (2 C), 127.51 (2 C), 128.41, 129.05, 134.99, 139.14, 144.91, 147.91, 150.67, 150.87; ES-MS *m/z* 453 [M + H]^+^; Anal.Calcd for C_24_H_22_ClN_3_O_2_S: C, 63.78; H, 4.91; N, 9.30; found: C, 63.74; H, 4.89; N, 9.28.

### 4-Chloro-N-(3-(7-chloroquinolin-4-ylamino)propyl)benzenesulfonamide (28)

Pale yellowish white solid; 72% yield; mp 117–119 °C; IR (KBr, cm^−1^): 3300.7 (NH); 1189.8 (SO_2_); ^1^H NMR (500 MHz, CDCl_3_): δ 1.79–1.83 (m, 2 H, CH_2_), 2.89–2.92 (m, 2 H, CH_2_), 3.76–3.79 (m, 2 H, CH_2_), 6.24–6.25 (d, *J* = 5.0 Hz, 1 H, Ar-*H*), 6.97 (br s, 1 H, N*H* D_2_O exchangeable), 7.27–7.29 (d, *J* = 10.0 Hz, 1 H, Ar-*H*), 7.42–7.44 (d, *J* = 10.0 Hz, 1 H, Ar-*H*), 7.42–7.44(d, *J* = 10.0 Hz, 2 H, Ar-*H*), 7.70–7.72 (d, *J* = 10.0 Hz, 1 H, Ar-*H*), 7.73–7.35 (d, *J* = 10.0 Hz, 1 H, Ar-*H*), 7.87–7.88 (d, *J* = 5.0 Hz, 1 H, Ar-*H*); 7.95 (br s, 1 H, N*H* D_2_O exchangeable), 8.33 (s, 1 H, Ar-*H*); ^13^C NMR (125 MHz, CDCl_3_): δ 28.36, 40.51, 41.50, 99.05, 117.94, 124.49, 124.58, 127.95, 128.83 (2 C), 129.63 (2 C), 133.81, 137.20, 140.57, 149.54, 150.43, 152.35; ES-MS *m/z* 411 [M + H]^+^; Anal.Calcd for C_18_H_17_Cl_2_N_3_O_2_S: C, 52.69; H, 4.18; N, 10.24; found: C, 52.71; H, 4.15; N, 10.22.

### 2,4-Dichloro-*N*-[3-(7-chloro-quinolin-4-ylamino)-propyl]-benzenesulfonamide (29)

Pale yellowish white solid; 68% yield; mp 145–147 °C; IR (KBr, cm^−1^): 3295.7 (NH); 1192.3 (SO_2_); ^1^H NMR (500 MHz, CDCl_3_): δ 1.75–1.78 (m, 2 H, CH_2_), 2.99–3.02 (m, 2 H, CH_2_), 3.18–3.22 (m, 2 H, CH_2_), 6.36–6.37 (d, *J* = 5.0 Hz, 1 H, Ar-*H*), 6.50 (br s, 1 H, N*H* D_2_O exchangeable), 7.43–7.45 (d, *J* = 10.0 Hz, 1 H, Ar-*H*), 7.46 (br s, 1 H, N*H* D_2_O exchangeable), 7.50–7.52 (d, *J* = 10.0 Hz, 1 H, Ar-*H*), 7.74–7.76 (d, *J* = 10.0 Hz, 2 H, Ar-*H*), 7.91–7.92 (d, *J* = 5.0 Hz, 1 H, Ar-*H*), 8.20–8.22 (d, *J* = 10.0 Hz, 1 H, Ar-*H*), 8.37–8.38 (d, *J* = 5.0 Hz, 1 H, Ar-*H*); ^13^C NMR (125 MHz, CDCl_3_): δ 28.39, 39.45, 41.30, 99.00, 117.90, 124.47 (2 C), 124.60, 127.89, 128.05, 131.51, 132.23, 132.33 (2 C), 133.83, 149.47, 150.43, 152.29; ES-MS *m/z* 446 [M + H]^+^; Anal.Calcd for C_18_H_16_Cl_3_N_3_O_2_S: C, 48.61; H, 3.63; N, 9.45; found: C, 48.59; H, 3.65; N, 9.43.

### *N*-(3-(7-chloroquinolin-4-ylamino)propyl)-3-nitrobenzenesulfonamide (30)

Pale yellowish white solid; 73% yield; IR (KBr, cm^−1^): 3280.9 (NH); 1190.7 (SO_2_); ^1^H NMR (500 MHz, DMSO*d*_6_ + CDCl_3_): δ 1.80–1.86 (m, 2 H, CH_2_), 2.95–2.98 (m, 2 H, CH_2_), 3.19 (br s, 1 H, N*H*), 3.26–3.30 (m, 2 H, CH_2_), 6.31–6.32 (d, *J* = 5.0 Hz, 1 H, Ar-*H*), 6.77 (br s, 1 H, N*H*), 7.26–7.28 (d, *J* = 10.0 Hz, 1 H, Ar-*H*), 7.53 (s, 1 H, Ar-*H*), 7.64–7.66 (d, *J* = 10.0 Hz, 1 H, Ar-*H*), 7.81–7.86 (m, 2 H, Ar-*H*), 8.09–8.11 (d, *J* = 10.0 Hz, 1 H, Ar-*H*), 8.25–8.27 (d, *J* = 10.0 Hz, 1 H, Ar-*H*), 8.62 (s, 1 H, Ar-*H*); ^13^C NMR (125 MHz, DMSO*d*_6_ + CDCl_3_): δ 27.07, 39.93, 39.98, 97.98, 117.00, 121.23, 122.73, 123.77, 125.99, 127.25, 129.97, 131.91, 133.60, 142.06, 147.43, 148.53, 149.58, 151.16; ES-MS *m/z* 422 [M + H]^+^; Anal.Calcd for C_18_H_17_ClN_4_O_4_S: C, 51.37; H, 4.07; N, 13.31; found: C, 51.33; H, 4.09; N, 13.29.

### *N*-[3-(7-Chloro-quinolin-4-ylamino)-propyl]-2,4-dinitro-benzenesulfonamide (31)

Yellow solid; 68% yield; mp 238–240 °C; IR (KBr, cm^−1^): 3310.6 (NH); 1185.3 (SO_2_); ^1^H NMR (500 MHz, CDCl_3_): δ 2.05–5.08 (m, 2 H, CH_2_), 2.29 (br s, 1 H, N*H* D_2_O exchangeable), 3.29–3.32 (m, 2 H, CH_2_), 3.61–3.65 (m, 2 H, CH_2_), 6.41–6.42 (d, *J* = 5.0 Hz, 1 H, Ar-*H*), 7.16–7.17 (d, *J* = 5.0 Hz, 1 H, Ar-*H*), 7.28 (br s, 1 H, N*H* D_2_O exchangeable), 7.33–7.35 (d, *J* = 10.0 Hz, 2 H, Ar-*H*), 7.75 (s, 1 H, Ar-*H*), 8.12–8.14 (d, *J* = 10.0 Hz, 1 H, Ar-*H*), 8.36–8.37 (d, *J* = 5.0 Hz, 1 H, Ar-*H*), 8.89 (s, 1 H, Ar-*H*); ^13^C NMR (125 MHz, CDCl_3_): δ 27.12 (2 C), 41.13 (2 C), 98.92, 115.26, 117.89, 124.00, 124.15, 124.44, 127.80, 130.21, 131.41, 135.35, 148.55, 149.26, 150.50, 151.93; ES-MS *m/z* 467 [M + H]^+^; Anal.Calcd for C_18_H_16_ClN_5_O_6_S: C, 46.41; H, 3.46; N, 15.03; found: C, 46.37; H, 3.49; N, 15.29.

### 5-Dimethylamino-naphthalene-1-sulfonic acid [3-(7-chloro-quinolin-4-ylamino)-propyl]-amide (32)

Pale yellowish white solid; 66% yield; mp 190–192 °C; IR (KBr, cm^−1^): 3300.5 (NH); 1188.7 (SO_2_); ^1^H NMR (500 MHz, CDCl_3_): δ 1.81–1.85 (m, 2 H, CH_2_), 2.94 (s, 6 H, N(C*H*_3_)_2_), 3.02–3.07 (m, 2 H, CH_2_), 3.49–3.52 (m, 2 H, CH_2_), 5.53 (br s, 1 H, N*H* D_2_O exchangeable), 5.69 (br s, 1 H, N*H* D_2_O exchangeable), 7.18–7.20 (d, *J* = 10.0 Hz, 1 H, Ar-*H*), 7.28 (s, 1 H, Ar-*H*), 7.34–7.35 (d, *J* = 5.0 Hz, 1 H, Ar-*H*), 7.52–7.55 (d, *J* = 10.0 Hz, 2 H, Ar-*H*), 7.66–7.68 (d, *J* = 5.0 Hz, 1 H, Ar-*H*), 7.89 (s, 1 H, Ar-*H*), 8.26–8.27 (d, *J* = 5.0 Hz, 1 H, Ar-*H*), 8.31–8.33 (d, *J* = 10.0 Hz, 1 H, Ar-*H*), 8.44 (s, 1 H, Ar-*H*), 8.55–8.57 (d, *J* = 10.0 Hz, 1 H, Ar-*H*); ES-MS *m/z* 470 [M + H]^+^; Anal.Calcd for C_24_H_25_ClN_4_O_2_S: C, 61.46; H, 5.37; N, 11.95; found: C, 61.49; H, 5.33; N, 11.98.

### 3-[3-(7-Chloro-quinolin-4-ylamino)-propylsulfamoyl]-thiophene-2-carboxylic acid methyl ester (33)

White solid; 72% yield; IR (KBr, cm^−1^): 3310.5 (NH); 1187.5 (SO_2_); ^1^H NMR (500 MHz, CDCl_3_): δ 1.98–2.01 (m, 2 H, CH_2_), 3.06–3.12 (m, 2 H, CH_2_), 3.59–3.62 (m, 2 H, CH_2_), 3.98 (s, 3 H, COOC*H*_3_), 6.41–6.42 (d, *J* = 5.0 Hz, 1 H, Ar-*H*), 6.64 (br s, 1 H, N*H* D_2_O exchangeable), 6.75 (br s, 1 H, N*H* D_2_O exchangeable), 7.38–7.39 (d, *J* = 5.0 Hz, 1 H, Ar-*H*), 7.53–7.55 (d, *J* = 10.0 Hz, 1 H, Ar-*H*), 7.56–7.58 (d, *J* = 10.0 Hz, 1 H, Ar-*H*), 7.97 (s, 1 H, Ar-*H*), 8.00–8.02 (d, *J* = 10.0 Hz, 1 H, Ar-*H*), 8.44–8.45 (d, *J* = 5.0 Hz, 1 H, Ar-*H*); ^13^C NMR (125 MHz, CDCl_3_): δ 27.94, 39.84, 40.55, 53.31, 98.37, 109.87, 117.89, 122.57, 126.05 (2 C), 130.67 (2 C), 130.92 (2 C), 131.17 (2 C), 144.46, 161.18; ES-MS *m/z* 441 [M + H]^+^; Anal.Calcd for C_18_H_18_ClN_3_O_4_S_2_: C, 49.14; H, 4.12; N, 9.55; found: C, 49.12; H, 4.09; N, 9.57.

### *N*-[3-(7-Trifluoromethyl-quinolin-4-ylamino)-propyl]-methanesulfonamide (34)

White solid; 69% yield; IR (KBr, cm^−1^): 3305.4 (NH); 1174.9 (SO_2_); mp 171–173 °C; ^1^H NMR (500 MHz, CDCl_3_): δ 1.88–1.93 (m, 2 H, CH_2_), 2.85 (s, 3 H, SO_2_C*H*_3_), 3.03–3.13 (m, 2 H, CH_2_), 3.40–3.46 (m, 2 H, CH_2_), 6.43–6.44 (d, *J* = 5.0 Hz, 1 H, Ar-*H*), 6.82 (br s, 1 H, N*H* D_2_O-exchangeable), 7.01 (br s, 1 H, N*H* D_2_O-exchangeable), 7.47–7.49 (d, *J* = 5.0 Hz, 1 H, Ar-*H*), 8.06 (s, 1 H, Ar-*H*), 8.18–8.20 (d, *J* = 10.0 Hz, 1 H, Ar-*H*), 8.47–8.48 (d, *J* = 5.0 Hz, 1 H, Ar-*H*); ^13^C NMR (125 MHz, CDCl_3_): δ 28.07, 39.63, 40.62, 99.95, 119.31, 121.09, 122.95, 123.10, 125.26, 126.94, 130.10, 147.81, 150.10, 152.24; ES-MS *m/z* 348 [M + H]^+^; Anal.Calcd for C_14_H_16_F_3_N_3_O_2_S: C, 48.41; H, 4.64; N, 12.10; found: C, 48.39; H, 4.67; N, 12.12.

### 4-Methyl-*N*-[3-(7-trifluoromethyl-quinolin-4-ylamino)-propyl]-benzenesulfonamide (35)

Pale yellowish white solid; 65% yield; IR (KBr, cm^−1^): 3290.6 (NH); 1189.8 (SO_2_); mp 77–79 °C; ^1^H NMR (500 MHz, CDCl_3_): δ 1.91–1.95 (m, 2 H, CH_2_), 2.41 (s, 3 H, CH_3_), 3.08–3.13 (m, 2 H, CH_2_), 3.49–3.52 (m, 2 H, CH_2_), 6.24 (br s, 1 H, N*H*), 6.33–6.34 (d, *J* = 5.0 Hz, 1 H, Ar-*H*), 6.55 (br s, 1 H, N*H*), 7.24–7.26 (d, *J* = 10.0 Hz, 2 H, Ar-*H*), 7.43–7.45 (dd, *J*_1_ = 5.0 Hz, *J*_2_ = 10.0 Hz, 1 H, Ar-*H*), 7.75–7.77 (d, *J* = 10.0 Hz, 2 H, Ar-*H*), 7.98–8.00 (d, *J* = 10.0 Hz, 1 H, Ar-*H*), 8.12 (s, 1 H, Ar-*H*), 8.47–8.48 (d, *J* = 5.0 Hz, 1 H, Ar-*H*); ES-MS *m/z* 424 [M + H]^+^; Anal.Calcd for C_20_H_20_F_3_N_3_O_2_S: C, 56.73; H, 4.76; N, 9.92; found: C, 56.70; H, 4.72; N, 9.89.

### Biphenyl-4-sulfonic acid [3-(7-trifluoromethyl-quinolin-4-ylamino)-propyl]-amide (36)

White solid; 72% yield; mp 110–112 °C; IR (KBr, cm^−1^): 3310.5 (NH); 1185.6 (SO_2_); ^1^H NMR (500 MHz, CDCl_3_): δ 1.96–1.98 (m, 2 H, CH_2_), 3.21–3.23 (m, 2 H, CH_2_), 3.58–3.62 (m, 2 H, CH_2_), 5.70 (br s, 1 H, N*H* D_2_O exchangeable), 6.40–6.41 (d, *J* = 5.0 Hz, 1 H, Ar-*H*), 7.43–7.51 (m, 6 H, Ar-*H*), 7.60 (br s, 1 H, N*H* D_2_O exchangeable), 7.64–7.66 (m, 2 H, Ar-*H*), 7.86–7.90 (m, 3 H, Ar-*H*), 8.27 (s, 1 H, Ar-*H*), 8.55–8.56 (d, *J* = 5.0 Hz, 1 H, Ar-*H*); ^13^C NMR (125 MHz, CDCl_3_): δ 27.90, 39.52, 40.53, 99.69, 120.25 (2 C), 120.57, 121.17, 127.27 (2 C), 127.44 (2 C), 127.90 (2 C), 128.64 (2 C), 129.11 (2 C), 138.19 (2 C), 139.04, 145.92, 147.63, 149.27, 152.08; ES-MS *m/z* 487 [M + H]^+^; Anal.Calcd for C_25_H_22_F_3_N_3_O_2_S: C, 61.84; H, 4.57; N, 8.65; found: C, 61.87; H, 4.55; N, 8.68.

### 4-Chloro-*N*-[3-(7-trifluoromethyl-quinolin-4-ylamino)-propyl]-benzenesulfonamide (37)

Pale yellowish white solid; 66% yield; mp 70–72 °C; IR (KBr, cm^−1^): 3275.5 (NH); 1170.3 (SO_2_); ^1^H NMR (500 MHz, CDCl_3_): δ 1.95–1.98 (m, 2 H, CH_2_), 3.11–3.15 (m, 2 H, CH_2_), 3.53–3.56 (m, 2 H, CH_2_), 6.47–6.48 (d, *J* = 5.0 Hz, 1 H, Ar-*H*), 6.68 (br s, 1 H, N*H* D_2_O exchangeable), 7.74–7.76 (d, *J* = 10.0 Hz, 2 H, Ar-*H*), 7.50–7.52 (d, *J* = 10.0 Hz, 1 H, Ar-*H*), 7.66 (br s, 1 H, N*H* D_2_O exchangeable), 7.75–7.77 (d, *J* = 10.0 Hz, 2 H, Ar-*H*), 8.05–8.07 (d, *J* = 10.0 Hz, 1 H, Ar-*H*), 8.09 (s, 1 H, Ar-*H*), 8.44–8.45 (d, *J* = 5.0 Hz, 1 H, Ar-*H*); ^13^C NMR (125 MHz, CDCl_3_): δ 27.79, 40.01, 40.56, 99.36, 120.11, 120.66, 122.14, 124.45, 127.45, 128.37 (2 C), 128.77, 129.51 (2 C), 138.36, 139.30, 145.31, 150.04, 150.65; ES-MS *m/z* 445 [M + H]^+^; Anal.Calcd for C_19_H_17_ClF_3_N_3_O_2_S: C, 51.41; H, 3.86; N, 9.47; found: C, 51.44; H, 3.89; N, 9.50.

### 2,4-Dichloro-*N*-[3-(7-trifluoromethyl-quinolin-4-ylamino)-propyl]-benzenesulfonamide (38)

White solid; 68% yield; mp 117–119 °C; IR (KBr, cm^−1^): 3260.5 (NH); 1185.6 (SO_2_); ^1^H NMR (500 MHz, CDCl_3_): δ 1.94–1.97 (m, 2 H, CH_2_), 3.12–3.16 (m, 2 H, CH_2_), 3.54–3.58 (m, 2 H, CH_2_), 5.69 (br s, 1 H, N*H* D_2_O exchangeable), 6.10 (br s, 1 H, N*H* D_2_O exchangeable), 6.48–6.49 (d, *J* = 5.0 Hz, 1 H, Ar-*H*), 7.38–7.40 (d, *J* = 10.0 Hz, 1 H, Ar-*H*), 7.47–4.49 (d, *J* = 10.0 Hz, 1 H, Ar-*H*), 7.56–7.58 (d, *J* = 10.0 Hz, 1 H, Ar-*H*), 7.80–7.82 (d, *J* = 10.0 Hz, 1 H, Ar-*H*), 7.88–7.90 (d, *J* = 10.0 Hz, 1 H, Ar-*H*), 8.25 (s, 1 H, Ar-*H*), 8.60–8.61 (d, *J* = 5.0 Hz, 1 H, Ar-*H*); ^13^C NMR (125 MHz, CDCl_3_): δ 27.89, 39.43, 40.43, 99.75, 120.23, 120.49, 121.07, 127.30, 127.69 (2 C), 131.57, 132.16 (2 C), 132.33, 135.50, 139.88, 147.55, 149.22, 152.11; ES-MS *m/z* 479 [M + H]^+^; Anal.Calcd for C_19_H_16_Cl_2_F_3_N_3_O_2_S: C, 47.71; H, 3.37; N, 8.79; found: C, 47.69; H, 3.39; N, 8.76.

### 3-Nitro-*N*-[3-(7-trifluoromethyl-quinolin-4-ylamino)-propyl]-benzenesulfonamide (39)

Pale yellowish white solid; 69% yield; mp 98–100 °C; IR (KBr, cm^−1^): 3295.0 (NH); 1180.8 (SO_2_); ^1^H NMR (500 MHz, CDCl_3_): δ 1.62–1.68 (m, 2 H, CH_2_), 2.62–2.68 (m, 2 H, CH_2_), 3.03–3.13 (m, 2 H, CH_2_), 6.11–6.12 (d, *J* = 5.0 Hz, 1 H, Ar-*H*), 6.43 (br s, 1 H, N*H* D_2_O exchangeable), 7.23–7.24 (d, *J* = 10.0 Hz, 1 H, Ar-*H*), 7.29 (s, 1 H, Ar-*H*), 7.35–7.37 (d, *J* = 10.0 Hz, 1 H, Ar-*H*), 7.64 (br s, 1 H, N*H* D_2_O exchangeable), 7.76 (s, 1 H, Ar-*H*), 7.87–7.89 (d, *J* = 10.0 Hz, 1 H, Ar-*H*), 8.08–8.10 (d, *J* = 10.0 Hz, 1 H, Ar-*H*), 8.20–8.21 (d, *J* = 5.0 Hz, 1 H, Ar-*H*), 8.42 (s, 1 H, Ar-*H*); ES-MS *m/z* 454 [M + H]^+^; Anal.Calcd for C_19_H_17_F_3_N_4_O_4_S: C, 50.22; H, 3.77; N, 12.33; found: C, 50.18; H, 3.81; N, 12.29.

### 2,4-Dinitro-*N*-[3-(7-trifluoromethyl-quinolin-4-ylamino)-propyl]-benzenesulfonamide (40)

Yellow solid; 66% yield; mp 198–200 °C; IR (KBr, cm^−1^): 3275.3 (NH); 1170.9 (SO_2_); ^1^H NMR (500 MHz, CDCl_3_): δ 2.04–2.08 (m, 2 H, CH_2_), 3.59–3.63 (m, 2 H, CH_2_), 3.69–3.72 (m, 2 H, CH_2_), 6.56–6.57 (d, *J* = 5.0 Hz, 1 H, Ar-*H*), 7.22–7.24 (d, *J* = 10.0 Hz, 1 H, Ar-*H*), 7.55 (br s, 1 H, N*H* D_2_O exchangeable), 7.60–7.62 (d, *J* = 10.0 Hz, 1 H, Ar-*H*), 8.05 (br s, 1 H, N*H* D_2_O exchangeable), 8.19–8.21 (d, *J* = 10.0 Hz, 1 H, Ar-*H*), 8.40–8.42 (d, *J* = 10.0 Hz, 1 H, Ar-*H*), 8.47–8.48 (d, *J* = 5.0 Hz, 1 H, Ar-*H*), 8.86–8.87 (d, *J* = 5.0 Hz, 1 H, Ar-*H*), 8.93 (s, 1 H, Ar-*H*); ^13^C NMR (125 MHz, CDCl_3_): δ 27.05, 41.16, 67.50, 100.06, 115.45, 119.20, 121.32, 123.43, 124.02, 124.15, 125.60, 126.80, 130.19, 130.26, 135.26, 147.92, 148.46, 150.18, 152.54; ES-MS *m/z* 500 [M + H]^+^; Anal.Calcd for C_19_H_16_F_3_N_5_O_6_S: C, 45.69; H, 3.23; N, 14.02; found: 45.71; H, 3.26; N, 14.06.

### 5-Dimethylamino-naphthalene-1-sulfonic acid [3-(7-trifluoromethyl-quinolin-4-ylamino)-propyl]-amide (41)

Pale yellowish white solid; 65% yield; mp 199–201 °C; IR (KBr, cm^−1^): 3275.6 (NH); 1180.9 (SO_2_); ^1^H NMR (500 MHz, CDCl_3_): δ 1.49–1.55 (m, 2 H, CH_2_), 2.28 (br s, 1 H, N*H* D_2_O exchangeable), 2.54 (s, 6 H, N(C*H*_3_)_2_), 2.72–2.76 (m, 2 H, CH_2_), 3.02–3.06 (m, 2 H, CH_2_), 5.98–5.99 (d, *J* = 5.0 Hz, 1 H, Ar-*H*), 6.40 (br s, 1 H, N*H* D_2_O exchangeable), 6.85–6.87 (d, *J* = 10.0 Hz, 1 H, Ar-*H*), 7.15–7.28 (m, 4 H, Ar-*H*), 7.84–7.87 (m, 2 H, Ar-*H*), 8.07–8.09 (d, *J* = 10.0 Hz, 1 H, Ar-*H*), 8.14 (s, 1 H, Ar-*H*), 8.15–8.16 (d, *J* = 5.0 Hz, 1 H, Ar-*H*); ^13^C NMR (125 MHz, CDCl_3_): δ 27.35, 39.39, 40.39, 45.11 (2 C), 99.31, 114.84, 118.90, 119.31 (2 C), 120.67, 122.50, 122.91, 126.44, 127.74, 128.53, 129.35, 129.55, 129.81, 130.26, 135.46, 147.19, 149.82, 151.58, 151.63; ES-MS *m/z* 502 [M + H]^+^; Anal.Calcd for C_25_H_25_F_3_N_4_O_2_S: C, 59.75; H, 5.01; N, 11.15; found: C, 59.77; H, 5.04; N, 11.11.

### 3-[3-(7-Trifluoromethyl-quinolin-4-ylamino)-propylsulfamoyl]-thiophene-2-carboxylic acid methyl ester (42)

White solid; 70% yield; mp 137–139 °C; IR (KBr, cm^−1^): 3305.6 (NH); 1189.8 (SO_2_); ^1^H NMR (500 MHz, CDCl_3_): δ 1.93–1.98 (m, 2 H, CH_2_), 3.12–3.16 (m, 2 H, CH_2_), 3.61–3.67 (m, 2 H, CH_2_), 3.98 (s, 3 H, COOC*H*_3_), 5.79 (br s, 1 H, N*H* D_2_O exchangeable), 6.50–6.51 (d, *J* = 5.0 Hz, 1 H, Ar-*H*), 6.55 (br s, 1 H, N*H* D_2_O exchangeable), 7.56–7.57 (d, *J* = 5.0 Hz, 1 H, Ar-*H*), 7.60–7.61 (d, *J* = 5.0 Hz, 1 H, Ar-*H*), 7.63–7.65 (d, *J* = 10.0 Hz, 1 H, Ar-*H*), 7.98–7.99 (d, *J* = 5.0 Hz, 1 H, Ar-*H*), 8.28 (s, 1 H, Ar-*H*), 8.62–8.63 (d, *J* = 5.0 Hz, 1 H, Ar-*H*); ^13^C NMR (125 MHz, CDCl_3_): δ 27.79, 39.94, 40.40, 53.31, 99.76, 120.19, 120.69, 121.11 (2 C), 127.69, 130.74, 130.79, 131.16 (2 C), 144.51, 147.90, 149.26, 152.23, 161.26; ES-MS *m/z* 474 [M + H]^+^; Anal.Calcd for C_19_H_18_F_3_N_3_O_4_S_2_: C, 48.20; H, 3.83; N, 8.87; found: C, 48.17; H, 3.81; N, 8.85.

### Cell lines

The human MDA-MB231, MCF7 and HeLa cell lines were purchased from American Tissue Culture Collection (ATCC) (Manassas, VA), and maintained in RPMI 1640 medium supplemented with 10% fetal bovine serum and 2 mM L-glutamine. 184B5 and MCF10A immortalized breast cells (ATCC) were maintained in mammary epithelial basal medium supplemented with an MEGM mammary epithelial singlequot kit (Cambrex). Cells were grown at 37 °C with 5% CO_2_, 95% air under the humidified conditions. Cell line authentication was carried out by Genetica DNA Laboratories (Burlington, NC) using a short tandem repeat (STR) profiling method (March 2015; July 2015; September 2016).

### Reagents

Chloroquine diphosphate and cisplatin were purchased from Sigma-Aldrich Canada Ltd (Oakaville, ON, Canada). All the compounds were dissolved in 10–20 mM dimethyl sulfoxide (DMSO) and stored at −20 °C until use. The stock solution was diluted in culture medium (0.1–100 μM) immediately before use. The final concentration of DMSO in the SRB-based cytotoxicity assays did not exceed 0.1%. To rule out that the DMSO concentration used may affect cell proliferation, culture medium containing equivalent concentration of DMSO was used as a negative control in all experiments. In all studies, the concentration of DMSO used did not notably show any antiproliferative effect.

### SRB assay

Antiproliferative/antigrowth effects were determined by a SRB-based protocol^6,23^. For a typical screening experiment, 5,000–10,000 cells were inoculated into 100 µL medium per well of a 96-well microtiter plate as described previously^8^. Briefly, after the inoculation, the microtiter plate was incubated at 37 °C, 5% CO_2_, 95% air and 100% relative humidity for 24 h, prior to addition of experimental drugs. Some of the sample wells were fixed with 25 µL of 50% tricholoroacetic acid (TCA) as a control of the cell population for each cell line at the time of drug addition (Tz). An aliquot of the frozen stock was thawed and diluted to a desired final maximum test-concentration with complete medium. Two to ten-fold serial dilutions were made to provide a total of seven drug concentrations (and a control [C]). Following addition of drugs, the culture plate was incubated for additional 48 h. Cells were fixed *in situ* by slowly adding 25 µL of ice-cold 50% (w/v) TCA (final concentration, 10% TCA), and were then incubated for 60 min at 4 °C. The supernatant was discarded, and the plate was washed five times with tap water, followed by air-dry. 50 µL of SRB solution at 0.4% (w/v) in 1% acetic acid was added to each well, and the plate was incubated for >30 min at room temperature. Unbound SRB was removed by five washes with tap water, followed by air-drying. The cells “stained” with SRB were solubilized with 10 mM trizma base, and the absorbance was read on an automated plate reader at a wavelength of 515–564 nm. The relative growth rate (%) was calculated for each of the compound concentrations according to the following formula:

$$({\rm{Ti}}-{\rm{Tz}})/({\rm{C}}-{\rm{Tz}})\times 100$$

In the formula, time zero (Tz), control growth (C), and OD for different concentration of tested compounds (Ti). The GI_50_ for each compound was obtained from a non-linear Sigmoidal dose-response (variable slope) curve which is fitted by GraphPad Prism v.4.03 software. Values were calculated for each of these parameters if the level of activity was reached. However, if the effect was not reached or was exceeded, the value for that parameter was expressed as greater or less than the maximum or minimum concentration tested.

### Flow cytometry

Cells (2.0 × 10^6^) were harvested by centrifugation at 1,000 rpm on a bench-top centrifuge for 5 min, followed by fixation with ice-cold ethanol (70%) for 30 min to overnight at −20 °C^13^. The ethanol was then removed by centrifugation, and cells were resuspended in 1 × PBS solution, followed by centrifuge. The cell pellet was than stained with propidium iodide (PI) master mix (100 μg/mL RNase A, 100 μg/mL PI, 0.3% Nonidet P-40 and 0.1% sodium citrate in distilled water) for 30 min at 37 °C. DNA content was measured using a Beckmann Coulter Cytomics FC500 (Beckman Coulter, Fullerton, CA), and the proportion of cell populations in G0/G1, S, and G2/M phases of cell cycle was calculated on the basis of DNA distribution histograms using CXP software.

### Microscopy and cell staining

All immunocytochemistry experiments were visualized by confocal microscopy using a Zeiss 510 Meta laser scanning microscope (Carl Zeiss) equipped with a 63× objective lens. Three lasers were utilized for excitation with the following band pass filter settings used for detection: Argon 488 nm (band pass 505–530), HeNe 543 nm (long pass 560) and 633 nm (long pass 650). All images were captured and analyzed using LSM 510 software included with the microscope (LSM Image Examiner, Carl Zeiss).

LysoTracker Red sDND-99 staining was carried out as recommended by the manufacturer (Molecular Probes, Eugene, OR). Briefly, cells grown on a cover slip were incubated in 50 nM LysoTracker Red DND-99 in serum-free RPMI-1640 medium for 30 min and then rinsed once with serum-free medium. The cells were washed three times with ice-cold medium, incubated in fresh medium for 45 min at 37 °C, and then washed once with fresh medium. Subsequently, the cells were examined by fluorescence microscopy using two filters (for green and red images) sequentially. Merging the two color images was done using Northern Eclipse software.

Western blot and densitometry were carried out as we described previously^29,30^.

## Supplementary information

Supplementary data information
