# Supplementary material for: Examination of novel 4-aminoquinoline derivatives designed and synthesized by a hybrid pharmacophore approach to enhance their anticancer activities
Source: Sci Rep. 2019 Apr 19;9:6315. doi: 10.1038/s41598-019-42816-4 (PMC6474902; doi:10.1038/s41598-019-42816-4)
Supplement: Supplementary file 1 — Supplementary data information [file 41598_2019_42816_MOESM1_ESM.pdf]

Supplementary Information (Figures S1-S6 & NMR spectral data)

Examination of novel 4-aminoquinoline derivatives designed and synthesized by a hybrid pharmacophore approach to enhance their anticancer activities

V. Raja Solomon[a,b], S. Pundir[a,c] and Hoyun Lee[a,b,c *]

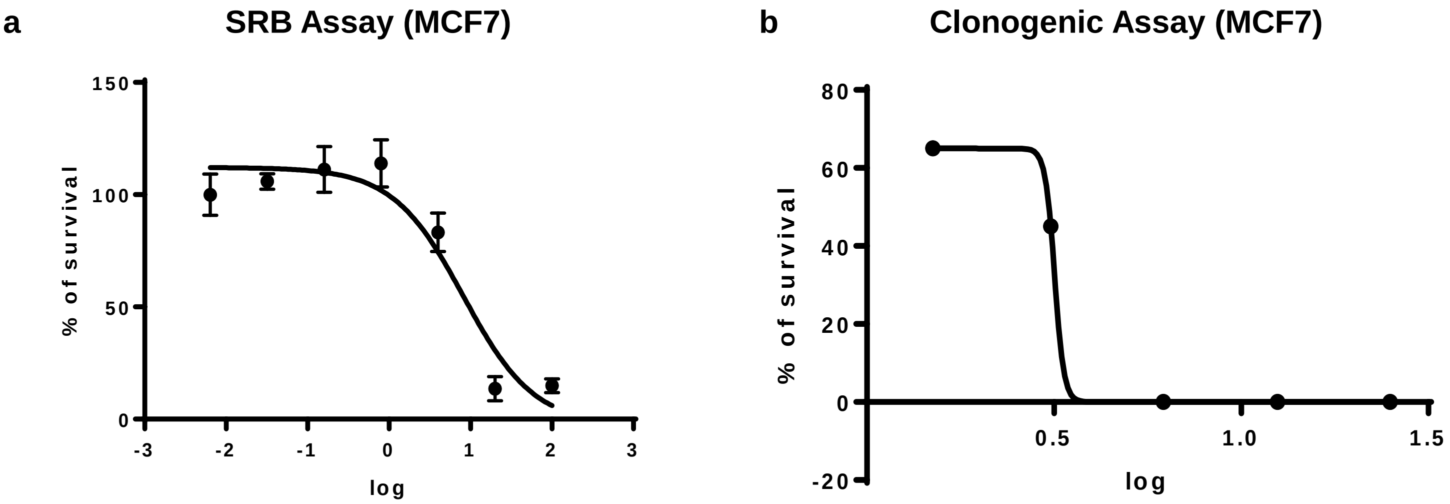

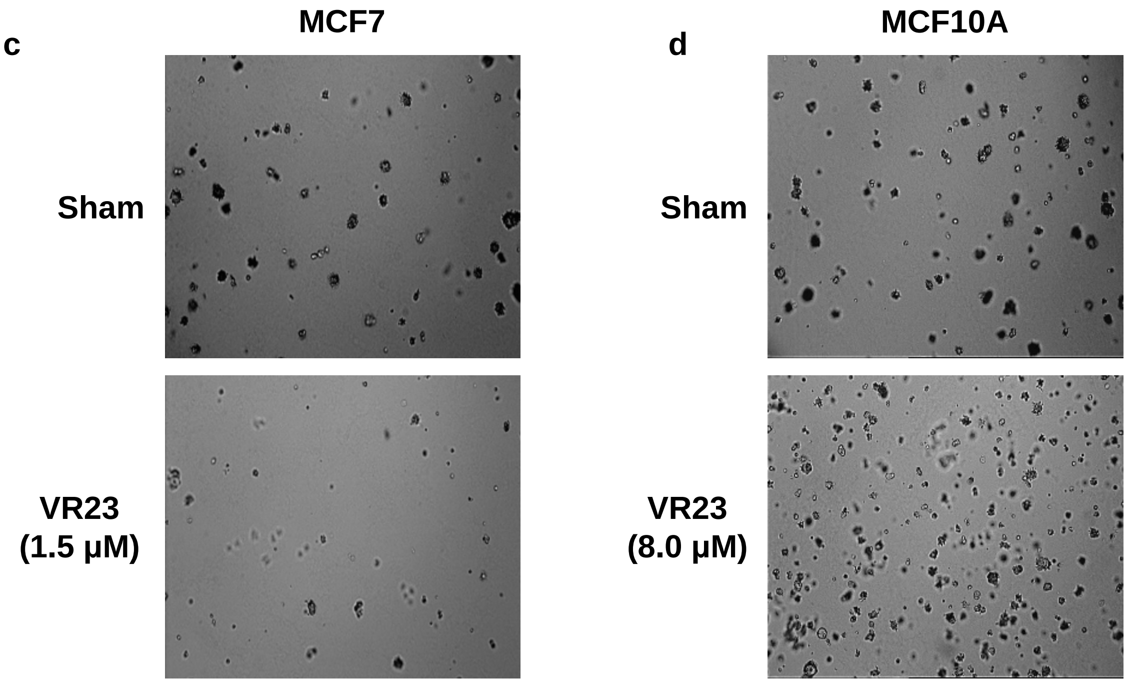

Supplementary Fig. S1. Determination of IC_{50} value using SRB (a) and clonogenic (b) assays. Panel c shows an example of clonogentic results, and panel d shows the growth of MCF10 in the absence (Sham) or presence of 8.0 μM compound **13** (VR23).

Developmental Therapeutics Program	NSC: D-787037 / 1	Conc: 1.00E-5 Molar	Test Date: Sep 08, 2015
One Dose Mean Graph	Experiment ID: 1509OS57		Report Date: Sep 25, 2015

Panel/Cell Line	Growth Percent
**Leukemia**	
CCRF-CEM	14.79
HL-60(TB)	75.70
K-562	18.03
MOLT-4	44.48
RPMI-8226	25.69
SR	3.12
**Non-Small Cell Lung Cancer**	
A549/ATCC	99.21
EKVX	87.87
HOP-62	96.42
HOP-92	82.07
NCI-H226	83.74
NCI-H23	97.20
NCI-H322M	106.97
NCI-H460	99.16
NCI-H522	46.06
**Colon Cancer**	
COLO 205	91.42
HCC-2998	105.53
HCT-116	77.52
HCT-15	66.61
HT29	93.47
KM12	102.98
SW-620	40.51
**CNS Cancer**	
SF-268	86.04
SF-295	99.96
SF-539	93.47
SNB-19	98.18
SNB-75	73.84
U251	93.90
**Melanoma**	
LOX IMVI	31.98
MALME-3M	76.16
M14	92.22
MDA-MB-435	98.10
SK-MEL-2	100.97
SK-MEL-28	107.87
SK-MEL-5	98.78
UACC-257	103.84
UACC-62	81.85
**Ovarian Cancer**	
IGROV1	99.48
OVCAR-3	93.02
OVCAR-4	95.26
OVCAR-5	106.30
OVCAR-8	97.11
NCI/ADR-RES	95.52
SK-OV-3	105.29
**Renal Cancer**	
786-0	80.78
A498	89.97
ACHN	84.34
CAKI-1	81.12
RXF 393	73.99
SN12C	88.19
TK-10	95.36
UO-31	55.40
**Prostate Cancer**	
PC-3	95.09
DU-145	92.29
**Breast Cancer**	
MCF7	52.67
MDA-MB-231/ATCC	85.27
HS 578T	89.08
BT-549	97.62
T-47D	60.29
MDA-MB-468	82.75
Mean	81.53
Delta	78.41
Range	104.75

Mean Growth Percent - Growth Percent

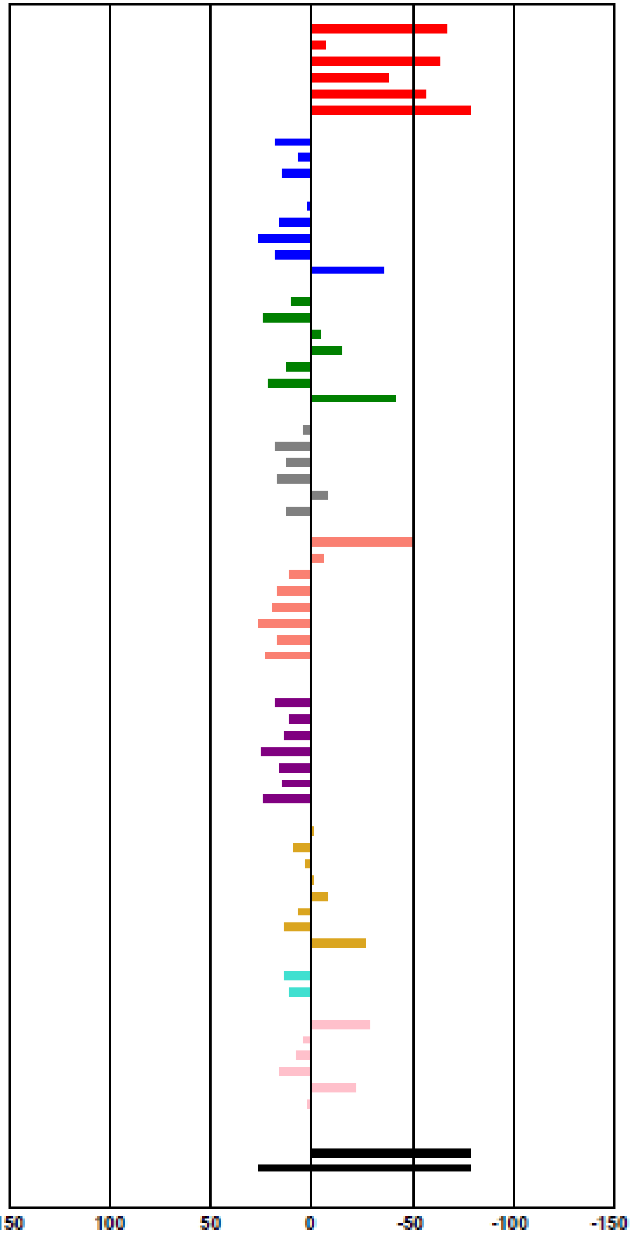

Supplementary Fig. S2. compound 13 shows effective growth inhibition on a wide range of cancers. Screening of compound **13** with the NCI-60 cancer panel was carried out by the USA National Cancer Institute.

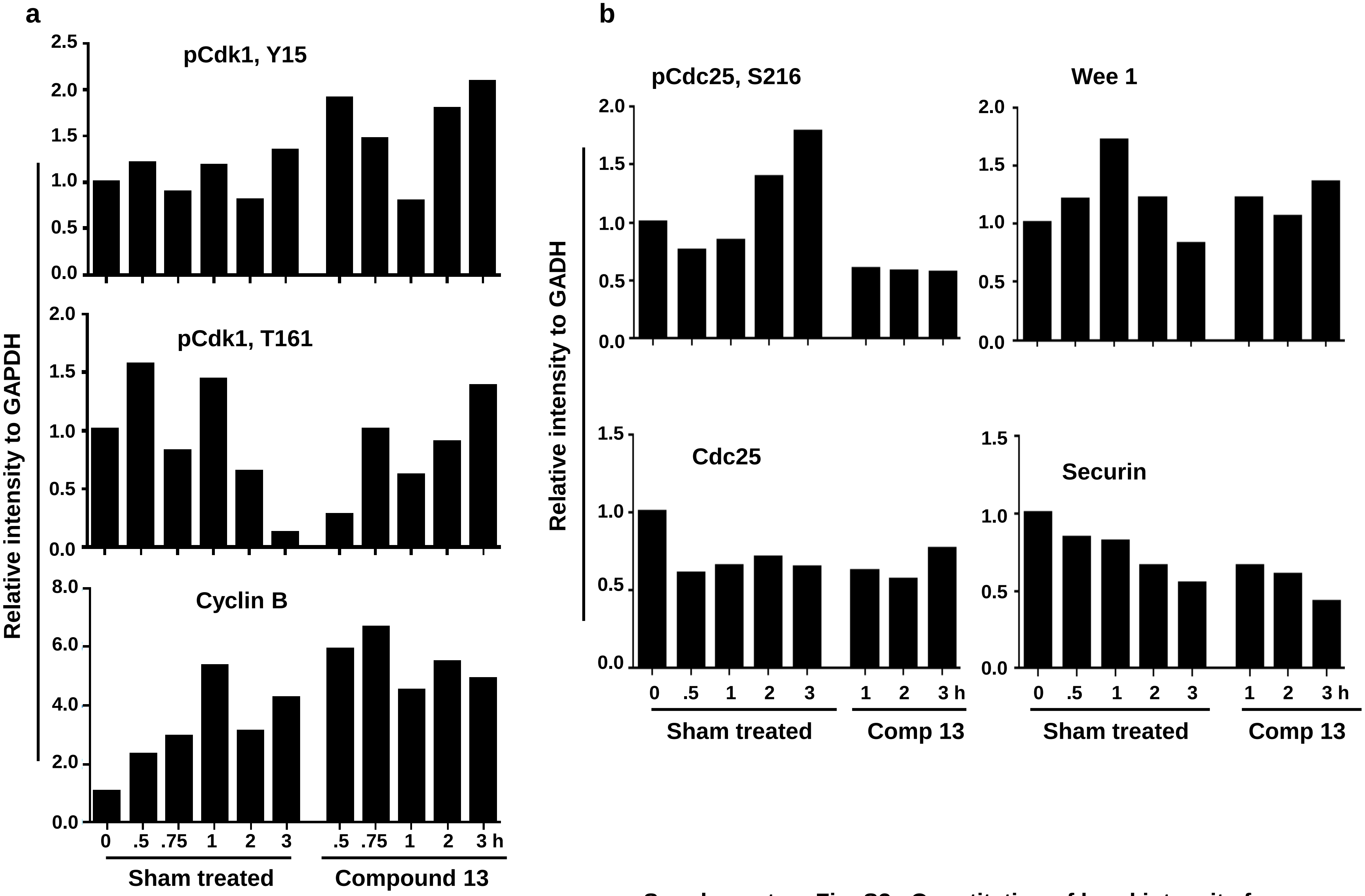

Supplementary Fig. S3. Quantitation of band intensity for those data shown in Fig. 5.

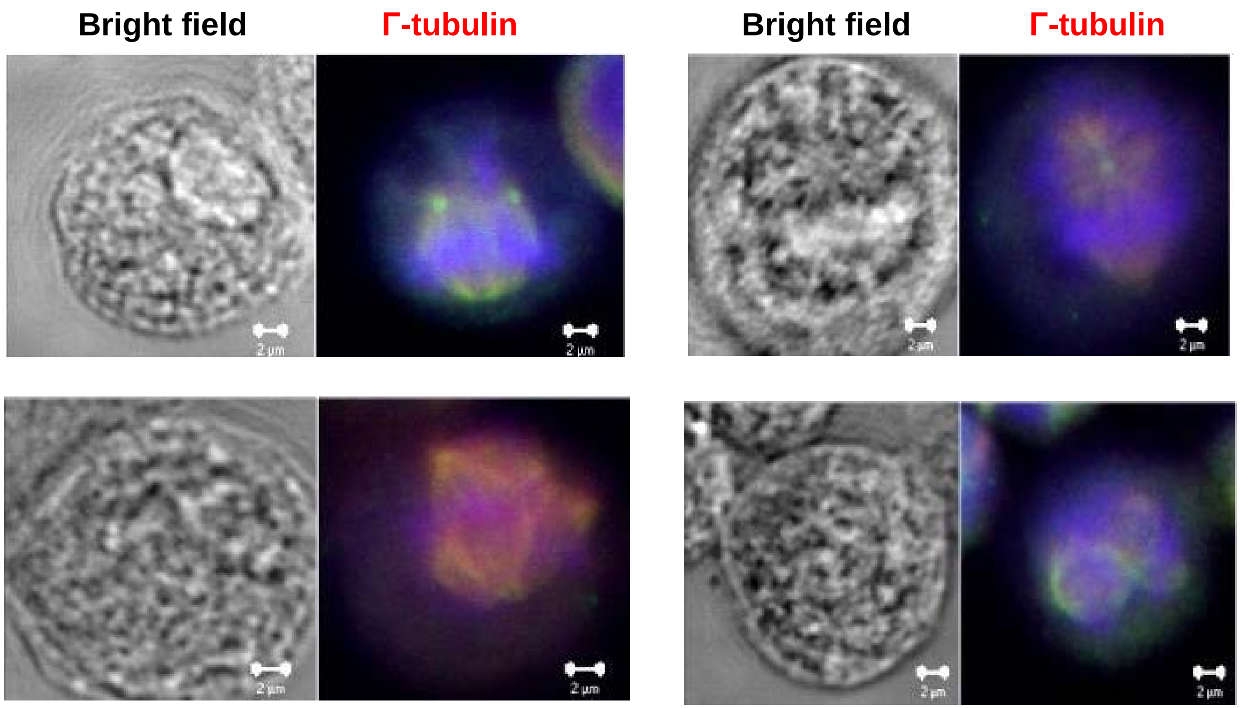

Supplementary Fig. S4. Examples of mitotic abnormality in MCF7 cells treated with 5 uM compound 13 (VR23) for 48 h.

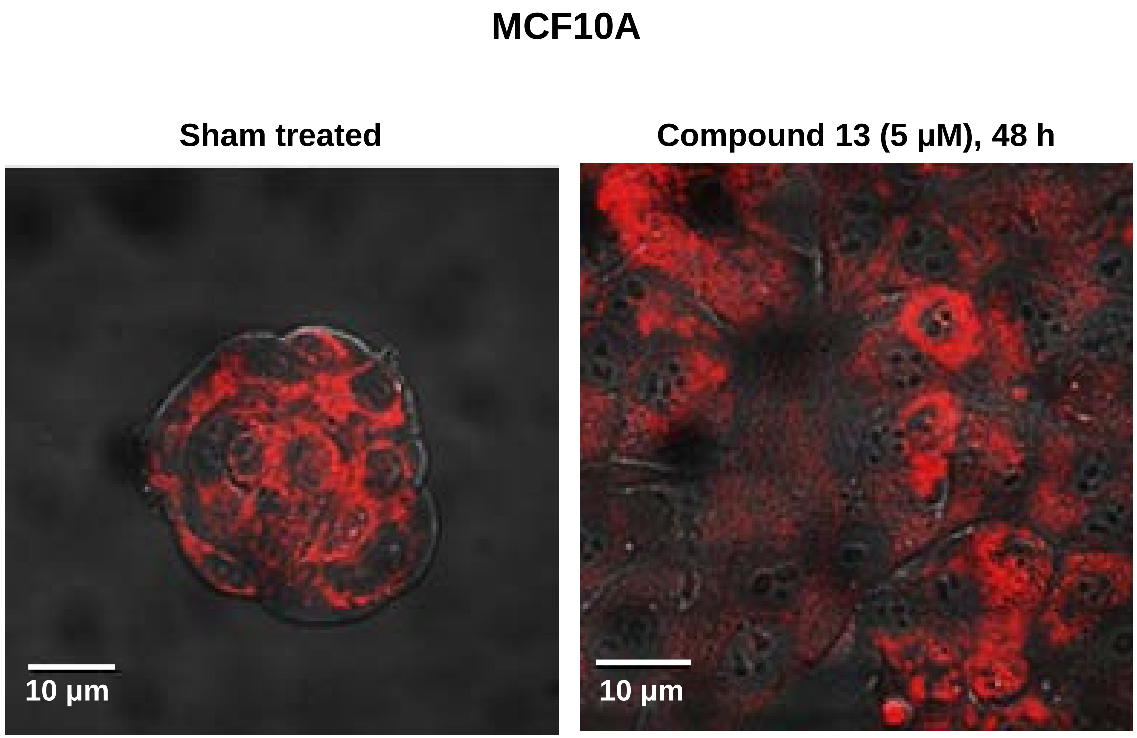

Supplementary Fig. S5. The lysosomal volume does not increase in response to 5 µM compound 13 (VR23) in non-cancer MCF10A, in contrast to cancer cells (MCF7, HeLa).

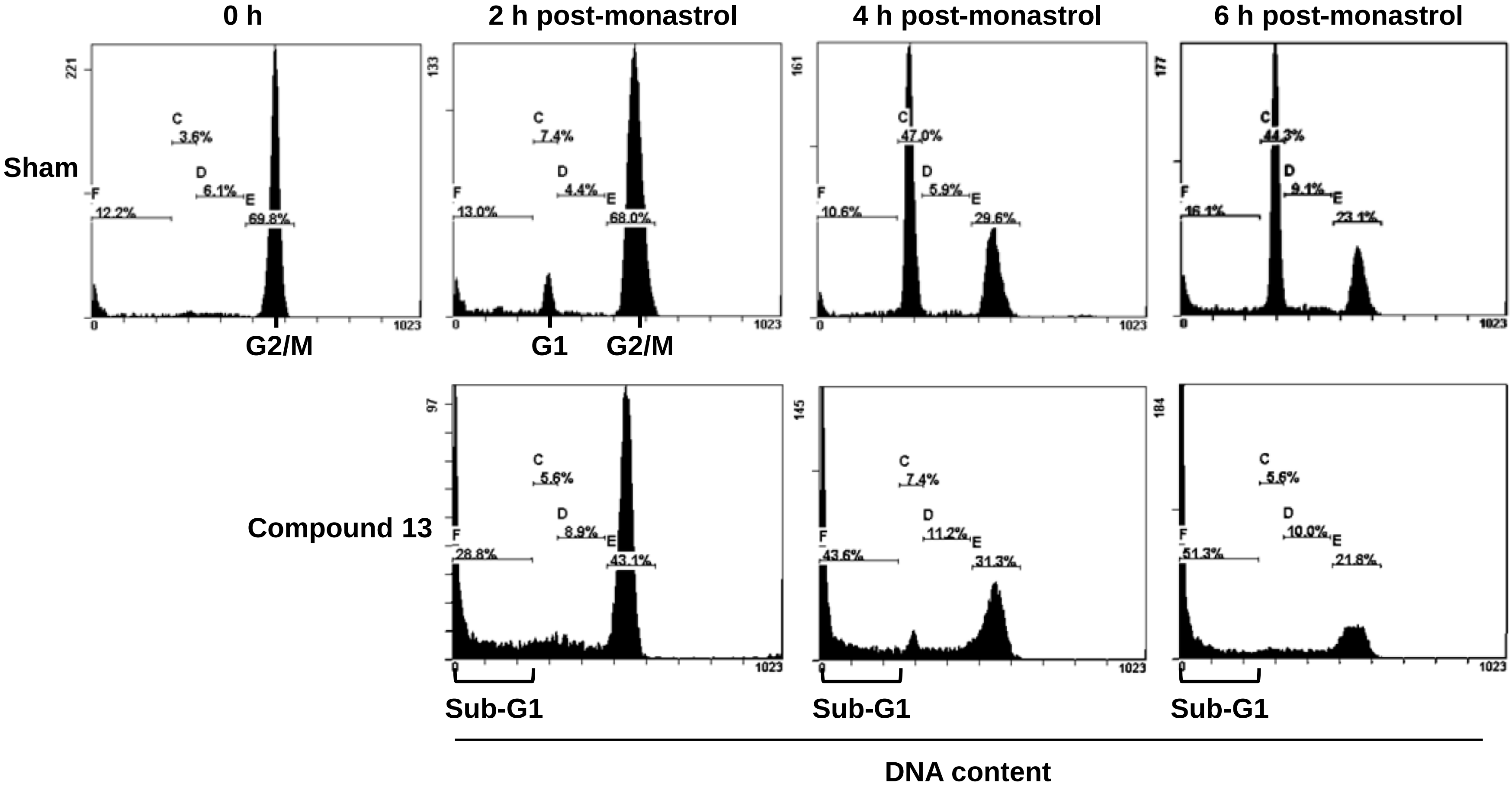

Supplementary Fig. S6. Sequential treatment of monastrol and compound 13 resulted in massive cell death. HeLa cells, treated with 100 μM of monastrol for 18 h (defined 0 h), were released into complete medium in the absence (sham) or presence of 10 μM compound 13 (VR23). Note that monastrol arrested cells at G2/M.

NMR Spectral data

Vt_20_13C_NMR1

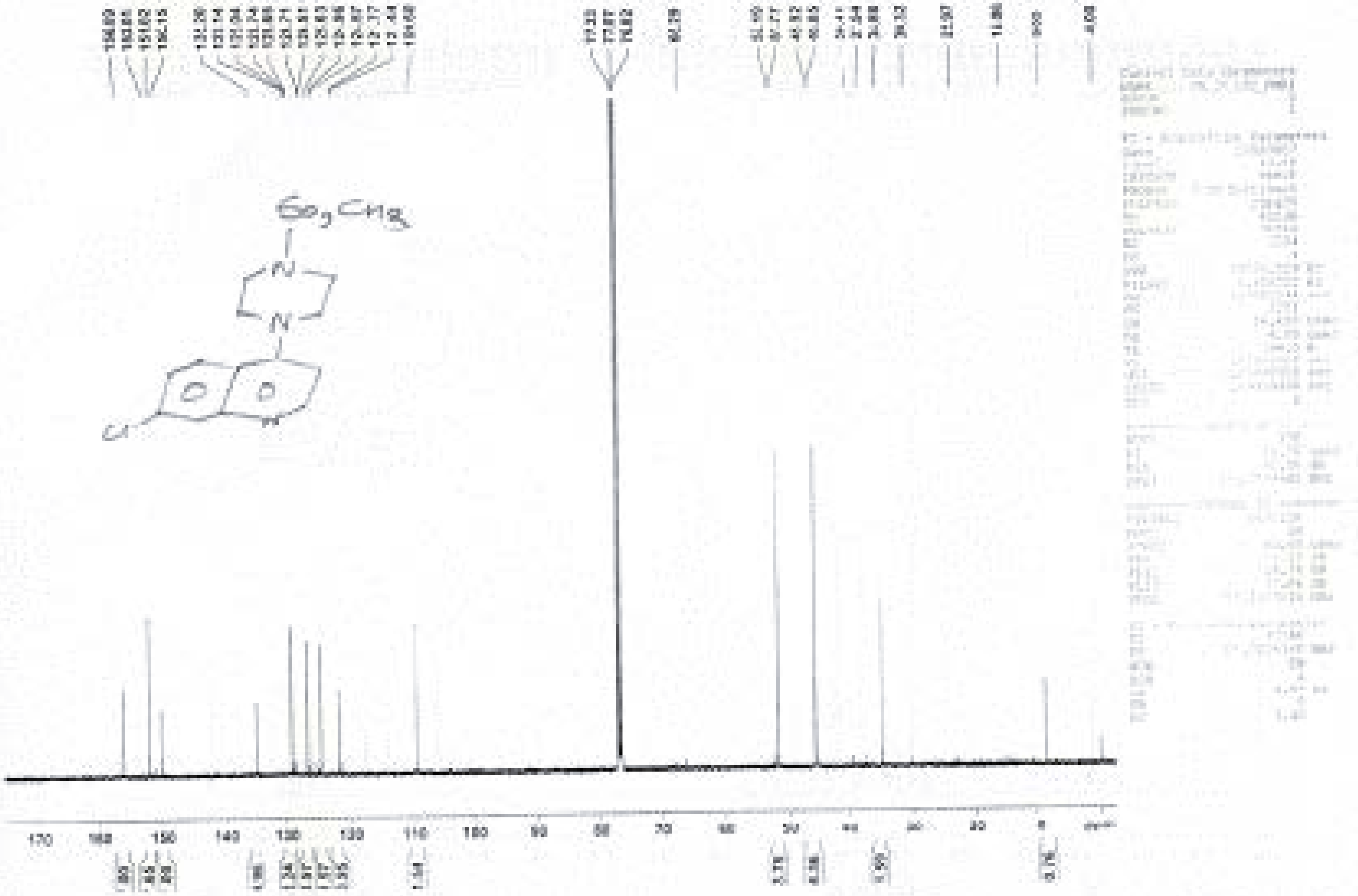

VR_21_1H_NMR

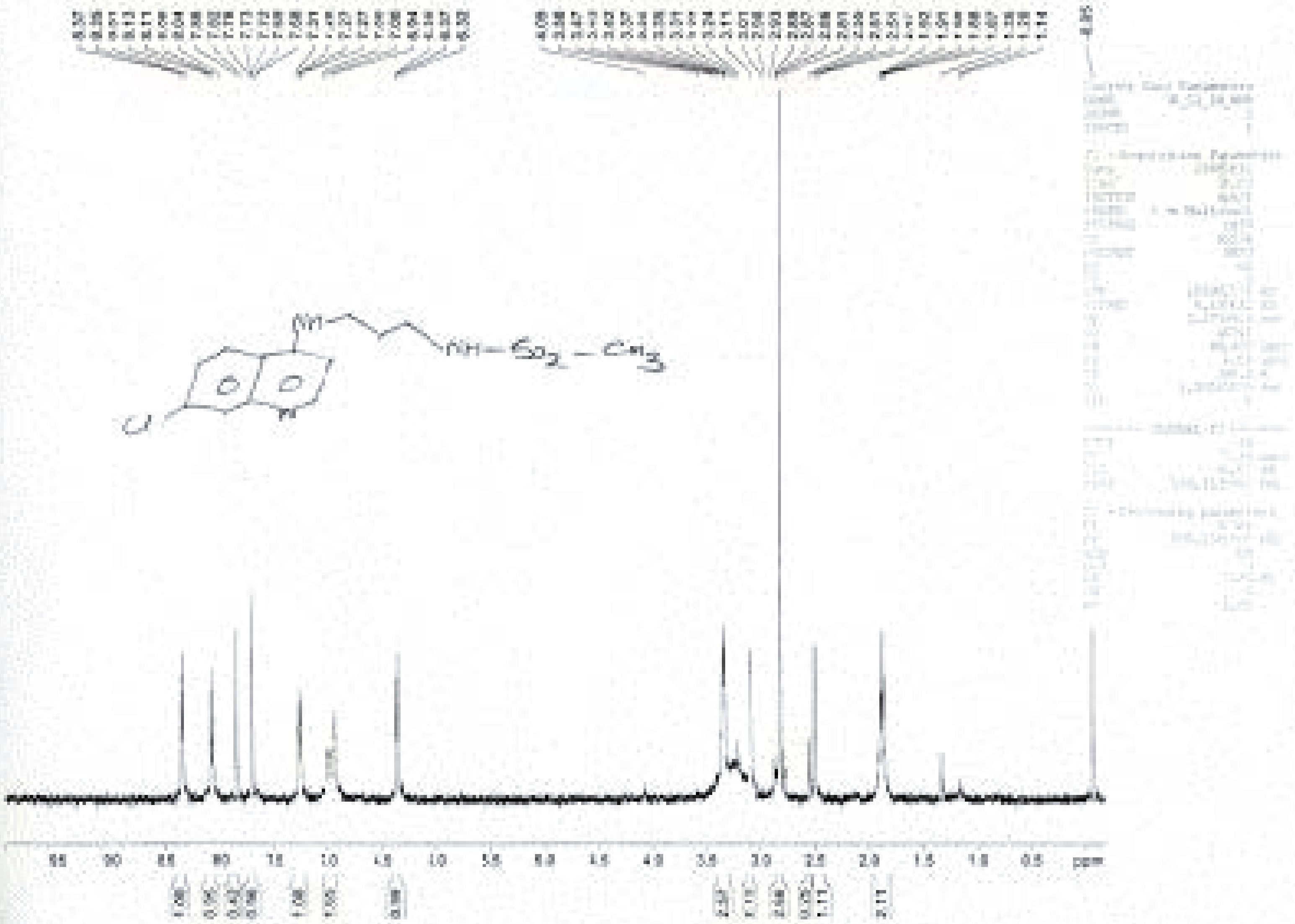

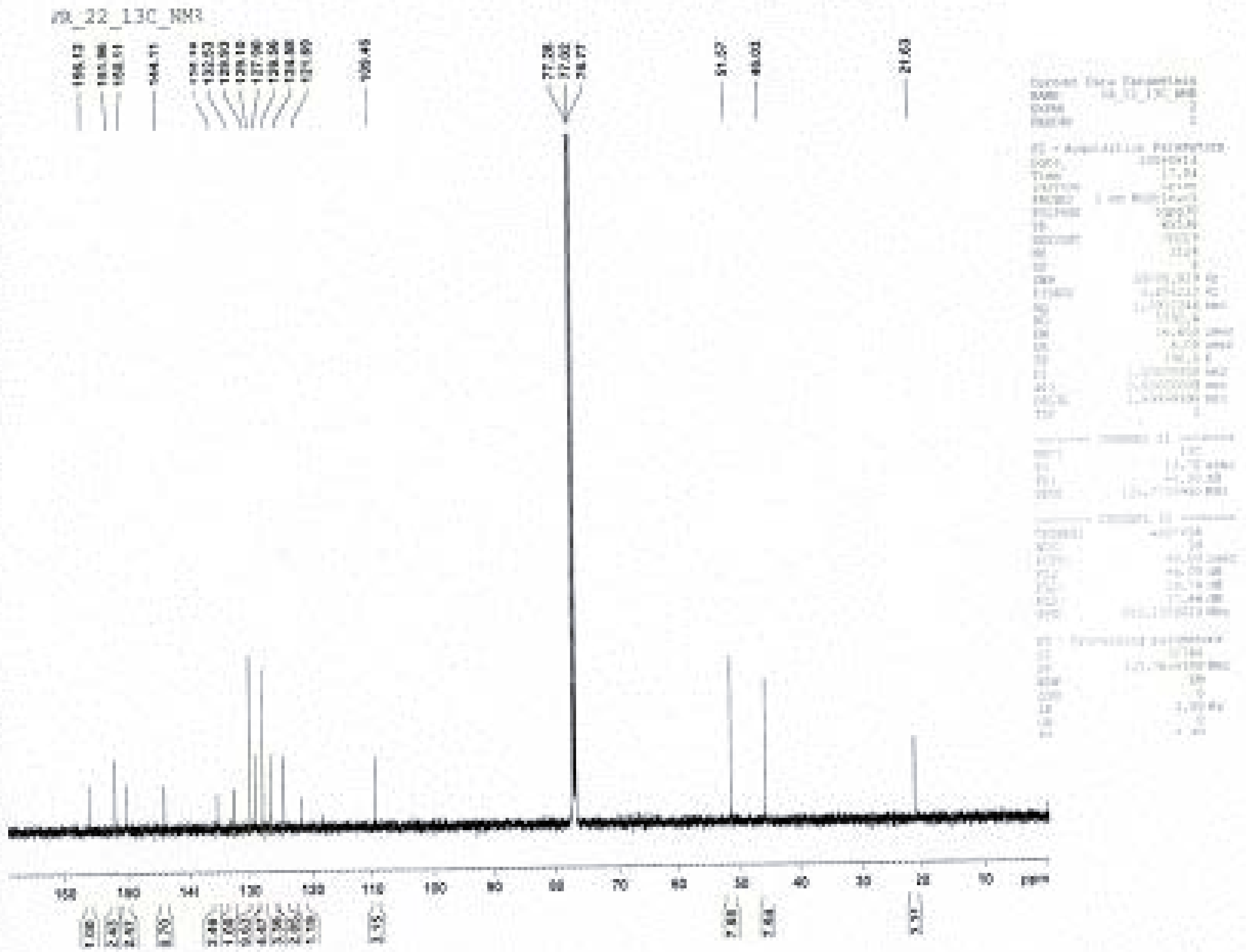

Purified & Confirmed

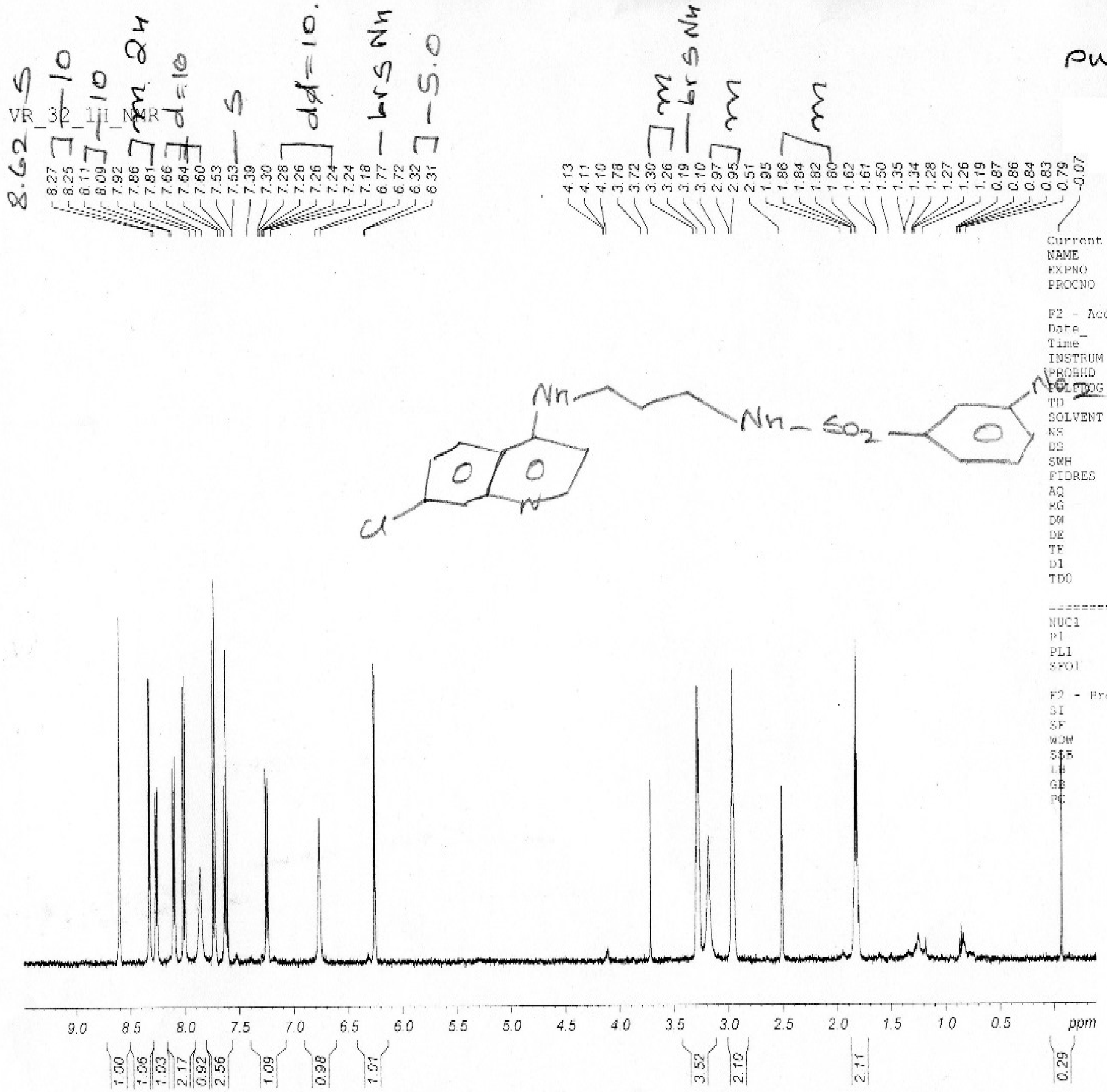

Current Data Parameters
NAME VR_32_1H_NMR
EXPNO 1
PROCNO 1

F2 - Acquisition Parameters
Date_ 20080908
Time 15.13
INSTRUM spect
PROBHD 5 mm Multinucl
PULPROG zg30
TD 65536
SOLVENT DMSO
NS 124
DS 2
SWH 10330.578 Hz
FIDRES 0.157632 Hz
AQ 3.1719923 sec
RG 46341
DW 48.400 usec
DE 6.00 usec
TE 298.0 K
D1 1.00000000 sec
TD0 1

======== CHANNEL f1 ========
NUC1 1H
P1 7.35 usec
PL1 -6.00 dB
SFO1 500.1530890 MHz

F2 - Processing parameters
SI 32768
SF 500.1500000 MHz
WDW EM
SSB 0
LB 0.30 Hz
GB 0
PC 1.00

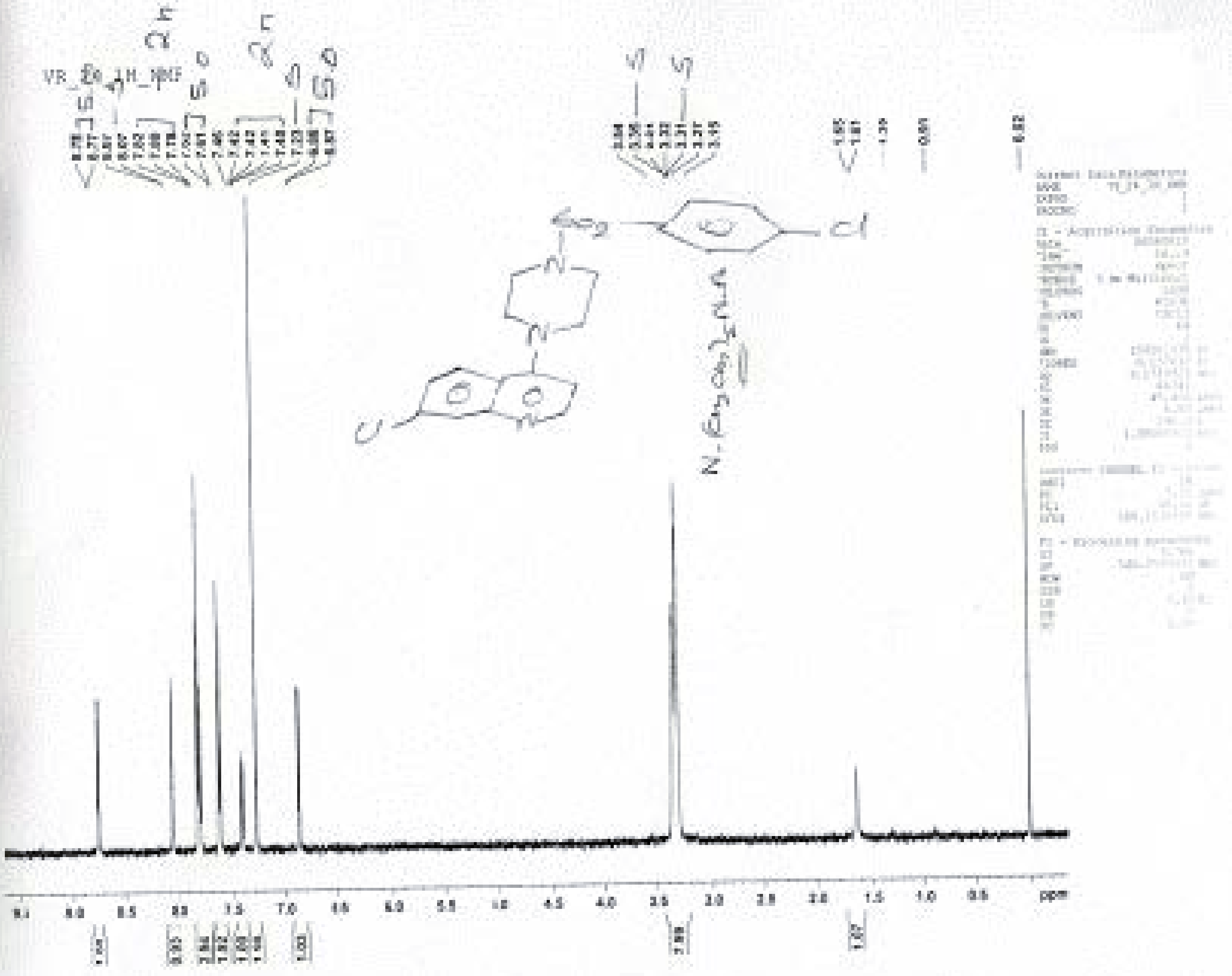

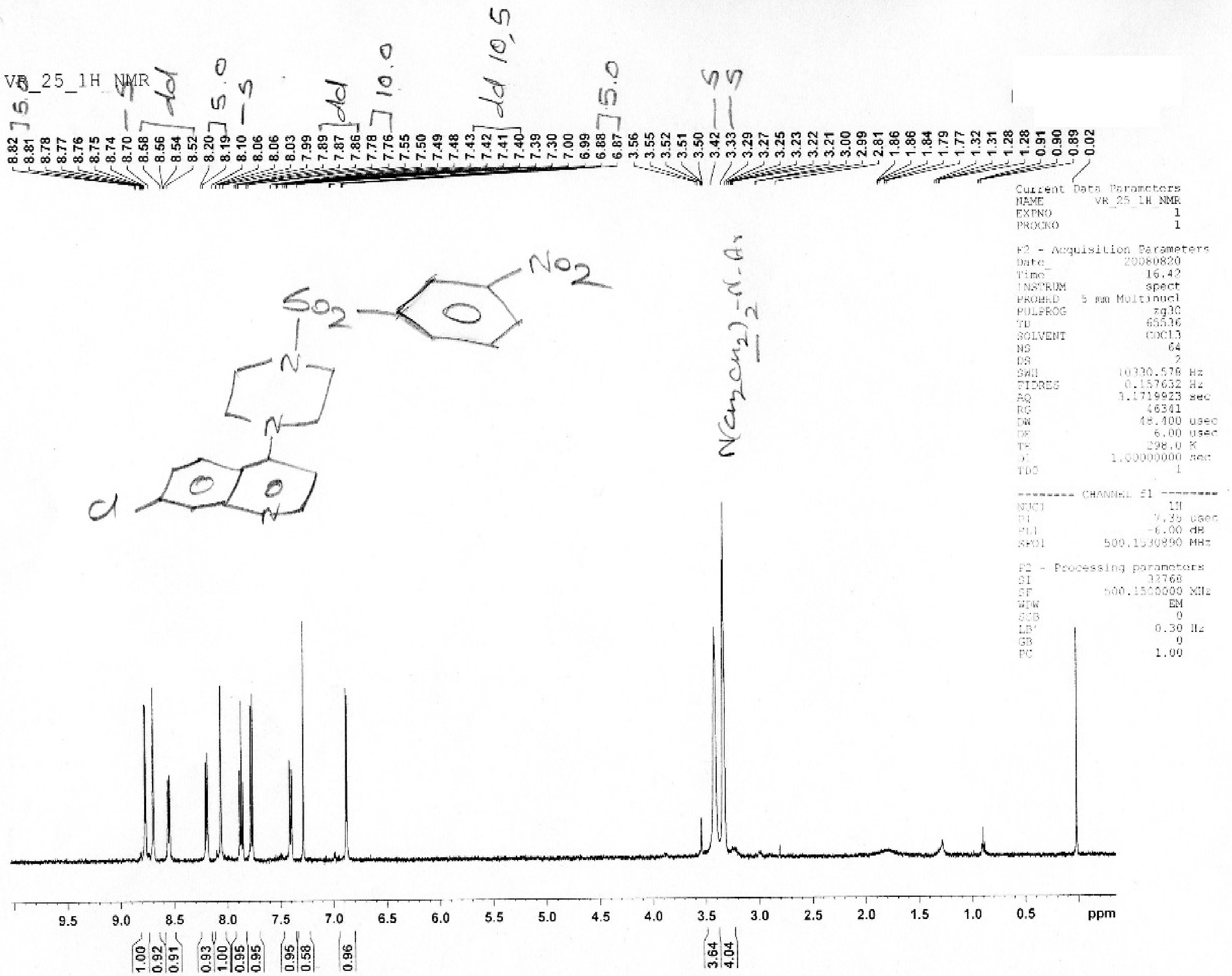
VR_25_1H NMR
8.82 8.81 8.78 8.77 8.76 8.75 8.74 8.70 8.58 8.56 8.54 8.52 8.20 8.19 8.10 8.06 8.03 7.99 7.89 7.87 7.86 7.78 7.76 7.55 7.50 7.49 7.48 7.43 7.42 7.41 7.40 7.39 7.30 7.00 6.99 6.88 6.87 3.56 3.55 3.52 3.51 3.50 3.42 3.33 3.29 3.27 3.25 3.23 3.22 3.21 3.00 2.99 2.81 1.86 1.86 1.84 1.79 1.77 1.32 1.31 1.28 1.28 0.91 0.90 0.89 0.02
Current Data Parameters
NAME VR_25_1H_NMR
EXPNO 1
PROCNO 1
F2 - Acquisition Parameters
Date_ 20080820
Time 16.42
INSTRUM spect
PROBHD 5 mm Multinucl
PULPROG zg30
TD 65536
SOLVENT CDCl3
NS 64
DS 2
SWH 10330.578 Hz
FIDRES 0.157632 Hz
AQ 3.1719923 sec
RG 46341
DW 48.400 usec
DE 6.00 usec
TE 298.0 K
D1 1.00000000 sec
TD0 1
======== CHANNEL f1 ========
NUC1 1H
P1 7.35 usec
PL1 -6.00 dB
SFO1 500.1530890 MHz
F2 - Processing parameters
SI 32768
SF 500.1500000 MHz
WDW EM
SSB 0
LB 0.30 Hz
GB 0
PC 1.00
9.5 9.0 8.5 8.0 7.5 7.0 6.5 6.0 5.5 5.0 4.5 4.0 3.5 3.0 2.5 2.0 1.5 1.0 0.5 ppm
1.00 0.92 0.91 0.93 1.00 0.95 0.95 0.95 0.58 0.96 3.64 4.04

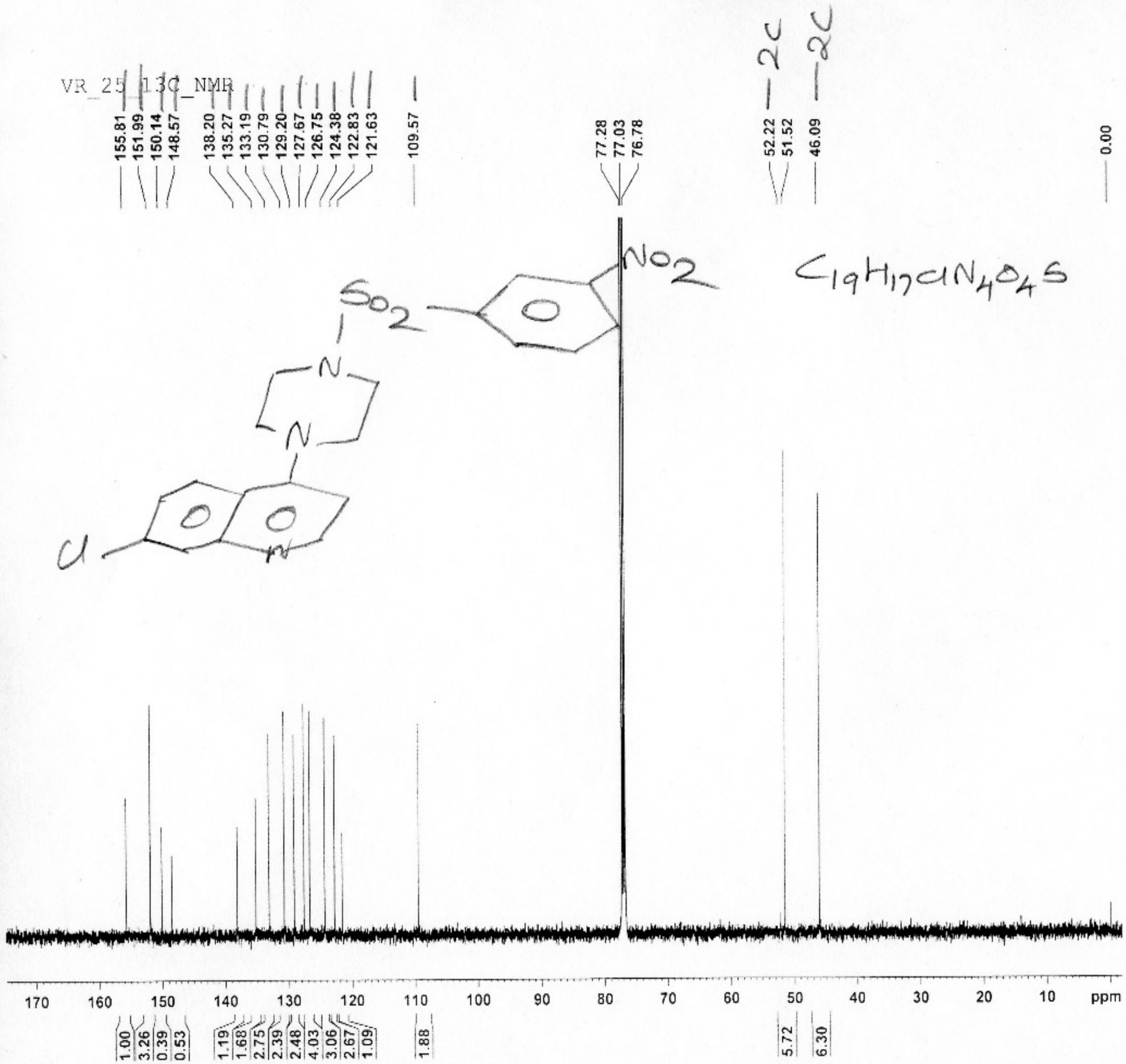

Current Data Parameters
NAME VR_25_13C_NMR
EXPNO 1
PROCNO 1

F2 - Acquisition Parameters
Date_ 20080821
Time 16.38
INSTRUM spect
PROBHD 5 mm Multinucl
PULPROG zgpg30
TD 65536
SOLVENT CDCl3
NS 5224
DS 4
SWH 30030.029 Hz
FIDRES 0.458222 Hz
AQ 1.0912244 sec
RG 8192
DW 16.650 usec
DE 6.00 usec
TE 298.0 K
D1 2.00000000 sec
d11 0.03000000 sec
DELTA 1.89999998 sec
TD0 1

======== CHANNEL f1 ========
NUC1 13C
P1 13.70 usec
PL1 -2.00 dB
SFO1 125.7753940 MHz

======== CHANNEL f2 ========
CPDPRG2 waltz16
NUC2 1H
PCPD2 90.00 usec
PL2 -6.00 dB
PL12 14.74 dB
PL13 17.66 dB
SFO2 500.1520010 MHz

F2 - Processing parameters
SI 32768
SF 125.7628180 MHz
WDW EM
SSB 0
LB 1.00 Hz
GB 0
PC 1.40

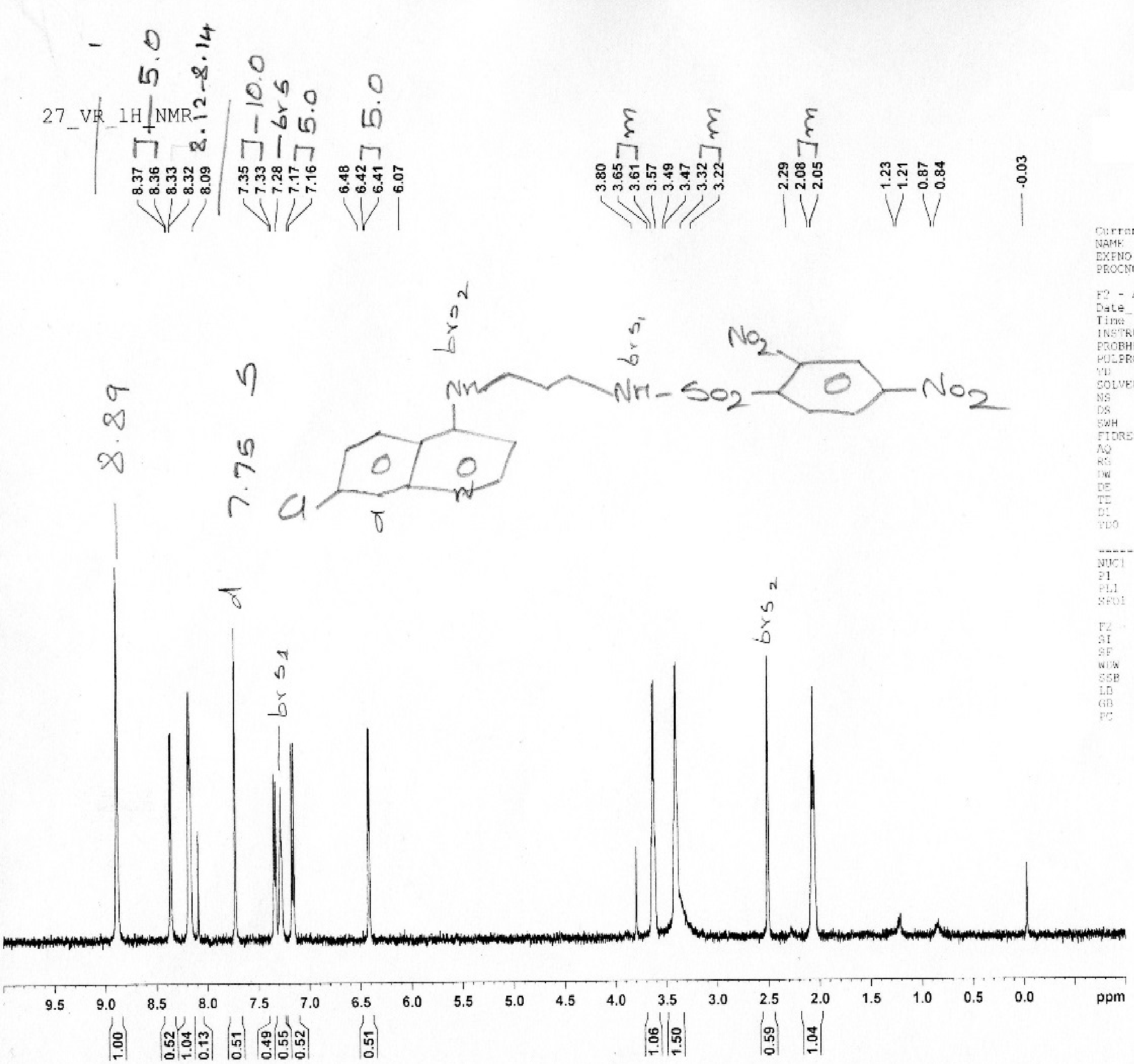

purified CC confirmed pure

Current Data Parameters
NAME 27_VR_1H_NMR
EXPNO 1
PROCNO 1

F2 - Acquisition Parameters
Date_ 20080826
Time 15.12
INSTRUM spect
PROBHD 5 mm Multinucl
PULPROG zg30
TD 65536
SOLVENT DMSO
NS 64
DS 2
SWH 10330.578 Hz
FIDRES 0.157632 Hz
AQ 3.1719923 sec
RG 46341
DW 48.400 usec
DE 6.00 usec
TE 298.0 K
D1 1.00000000 sec
TD0 1

======== CHANNEL f1 ========
NUC1 1H
P1 7.35 usec
PL1 -6.00 dB
SFO1 500.1530890 MHz

F2 - Processing parameters
SI 32768
SF 500.1500000 MHz
WDW EM
SSB 0
LB 0.30 Hz
GB 0
PC 1.00

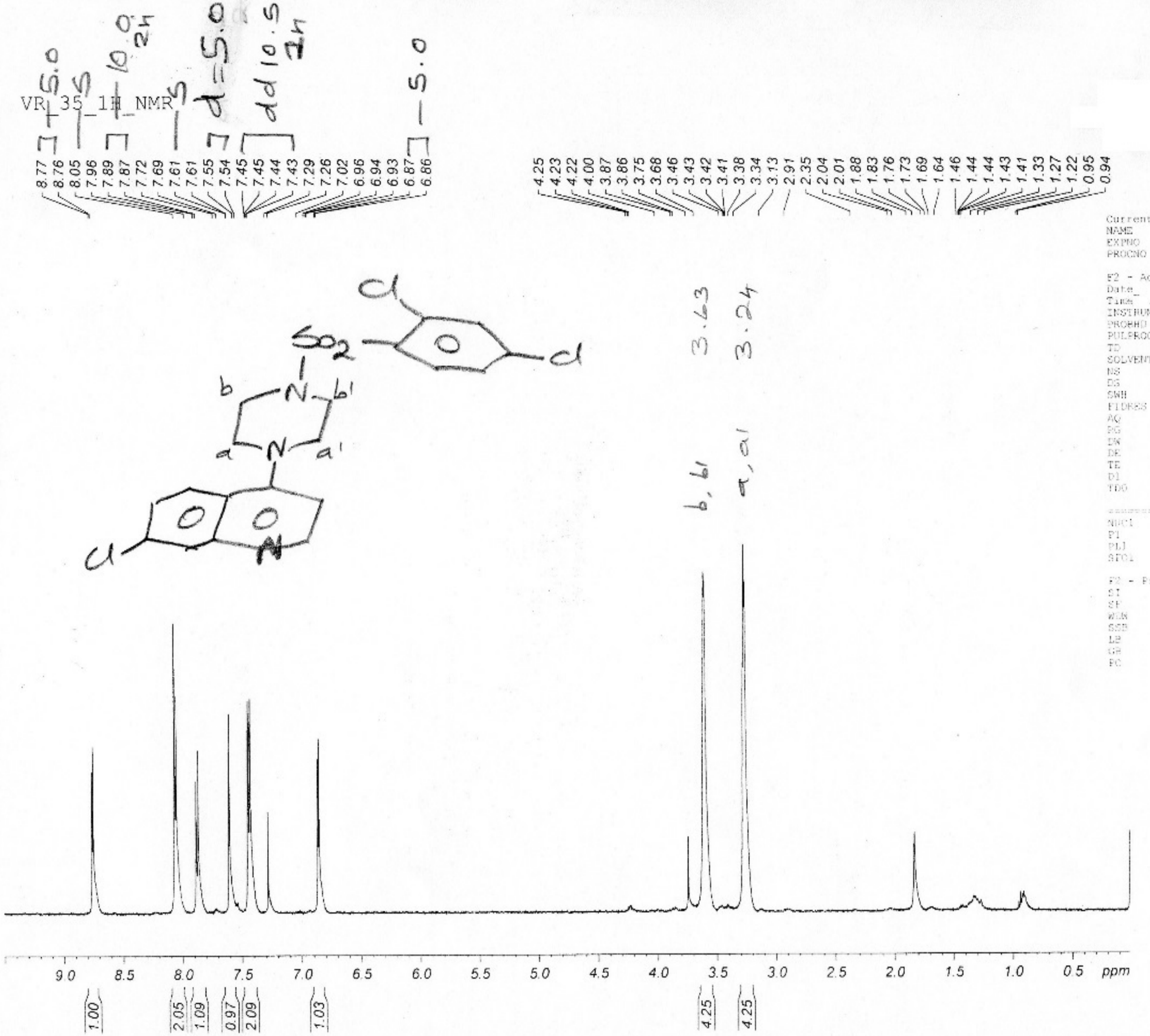

Current Data Parameters	
NAME	VR_35_1H_NMR
EXPNO	1
PROCNO	1
F2 - Acquisition Parameters	
Date_	20080922
Time	12.38
INSTRUM	spect
PROBHD	5 mm Multinucl
PULPROG	zg30
TD	65536
SOLVENT	CDCl3
NS	64
DS	2
SWH	10330.578 Hz
FIDRES	0.157632 Hz
AQ	3.1719923 sec
RG	20642.5
DW	48.400 usec
DE	6.00 usec
TE	298.0 K
D1	1.00000000 sec
TD0	1
======== CHANNEL f1 ========	
NUC1	1H
P1	7.35 usec
PL1	-6.00 dB
SFO1	500.1530890 MHz
F2 - Processing parameters	
SI	32768
SF	500.1500000 MHz
WDW	EM
SSB	0
LB	0.30 Hz
GB	0
PC	1.00

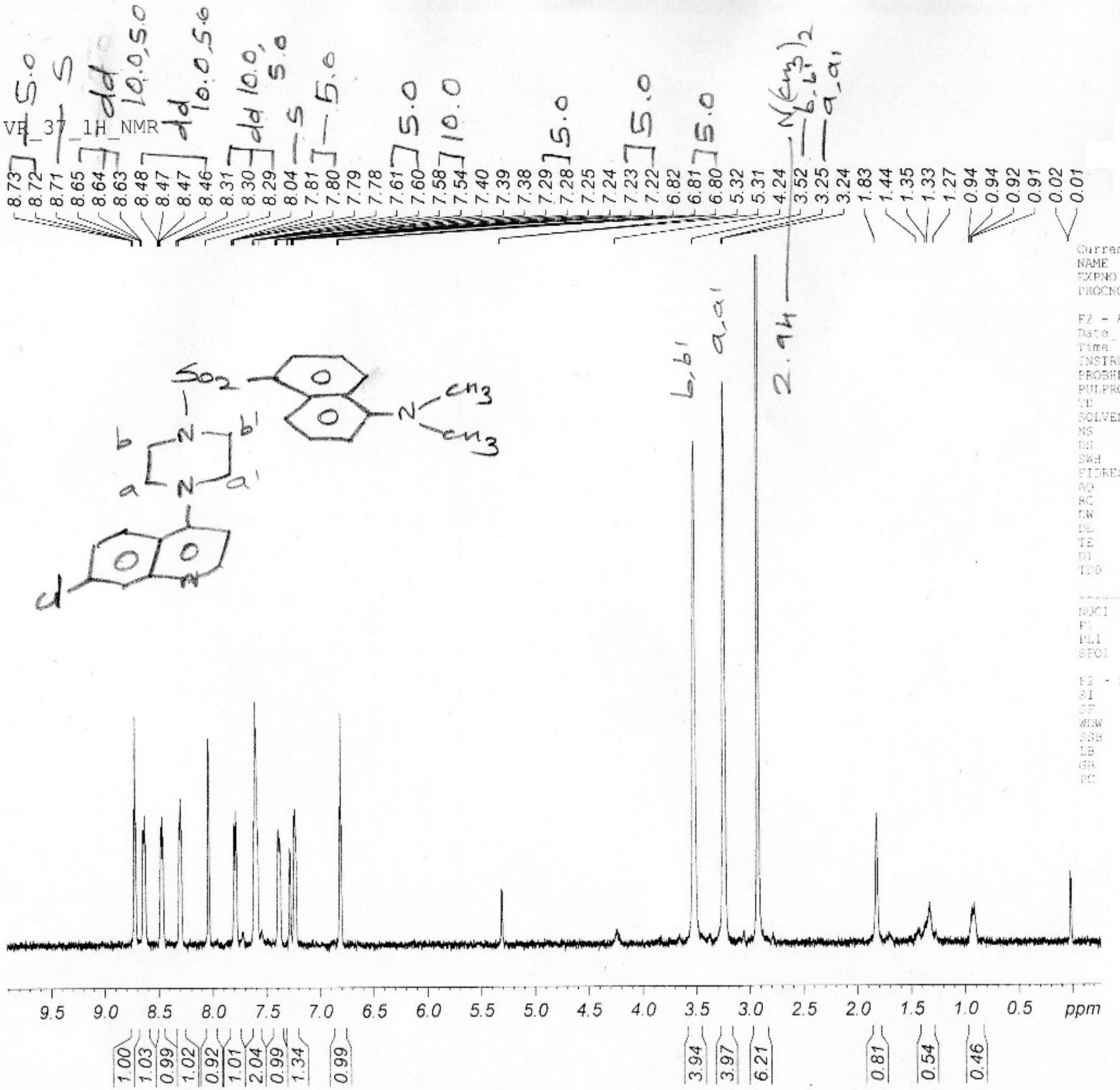


```
Current Data Parameters
NAME          VR_37_1H_NMR
EXPNO                    1
PROCNO                   1

F2 - Acquisition Parameters
Date_             20081001
Time                 11.07
INSTRUM              spect
PROBHD   5 mm Multinucl
PULPROG               zg30
TD                   65536
SOLVENT              CDCl3
NS                      64
DS                       2
SWH             10330.578 Hz
FIDRES           0.157632 Hz
AQ              3.1719923 sec
RC                   46341
DW                  48.400 usec
DE                    6.00 usec
TE                   298.0 K
D1             1.00000000 sec
TD0                      1

======== CHANNEL f1 ========
NUC1                    1H
P1                    7.35 usec
PL1                  -6.00 dB
SFO1           500.1530890 MHz

F2 - Processing parameters
SI                   32768
SF             500.1500000 MHz
WDW                     EM
SSB                      0
LB                    0.30 Hz
GB                       0
PC                    1.00
```

VR_38_1H_NMR

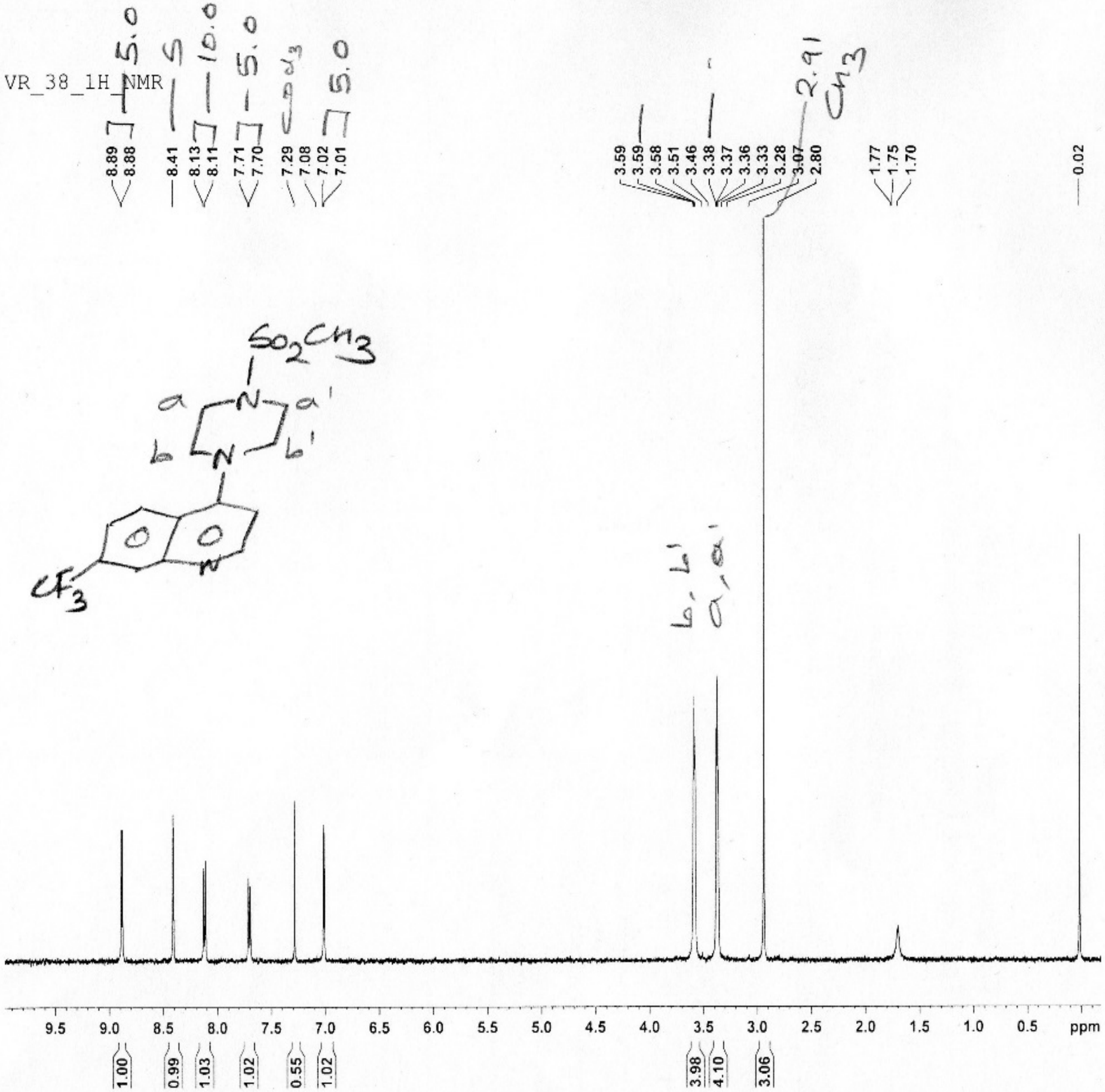

Current Data Parameters
NAME VR_38_1H_NMR
EXPNO 2
PROCNO 1

F2 - Acquisition Parameters
Date_ 20081002
Time 15.25
INSTRUM spect
PROBHD 5 mm Multinucl
PULPROG zg30
TD 65536
SOLVENT CDCl3
NS 64
DS 2
SWH 10330.578 Hz
FIDRES 0.157632 Hz
AQ 3.1719923 sec
RG 46341
DW 48.400 usec
DE 6.00 usec
TE 298.0 K
D1 1.00000000 sec
TD0 1

======== CHANNEL f1 ========
NUC1 1H
P1 7.35 usec
PL1 -6.00 dB
SFO1 500.1530890 MHz

F2 - Processing parameters
SI 32768
SF 500.1500000 MHz
WDW EM
SSB 0
LB 0.30 Hz
GB 0
PC 1.00

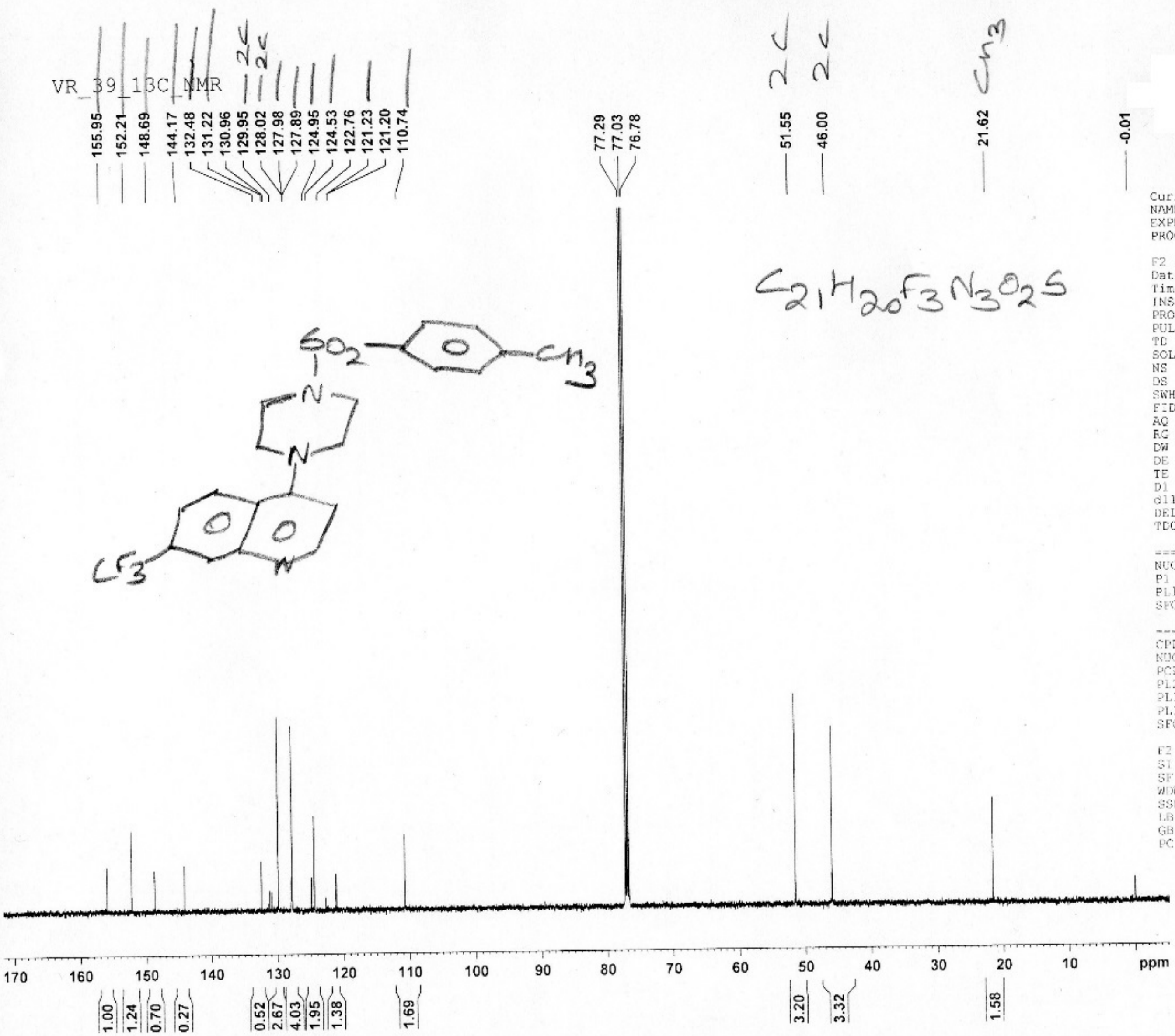

Current Data Parameters	
NAME	VR_39_13C_NMR
EXPNO	1
PROCNO	1

F2 - Acquisition Parameters	
Date_	20081003
Time	16.14
INSTRUM	spect
PROBHD	5 mm Multinucl
PULPROG	zgpg30
TD	65536
SOLVENT	CDCl3
NS	2048
DS	4
SWH	30030.029 Hz
FIDRES	0.458222 Hz
AQ	1.0912244 sec
RG	11585.2
DW	16.650 usec
DE	6.00 usec
TE	299.0 K
D1	2.00000000 sec
d11	0.03000000 sec
DELTA	1.89999998 sec
TD0	1

======== CHANNEL f1 ========	
NUC1	13C
P1	13.70 usec
PL1	-2.00 dB
SFO1	125.7753940 MHz

======== CHANNEL f2 ========	
CPDPRG2	waltz16
NUC2	1H
PCPD2	80.00 usec
PL2	-6.00 dB
PL12	14.74 dB
PL13	17.66 dB
SFO2	500.1520010 MHz

F2 - Processing parameters	
SI	32768
SF	125.7628180 MHz
WDW	EM
SSB	0
LB	1.00 Hz
GB	0
PC	1.40

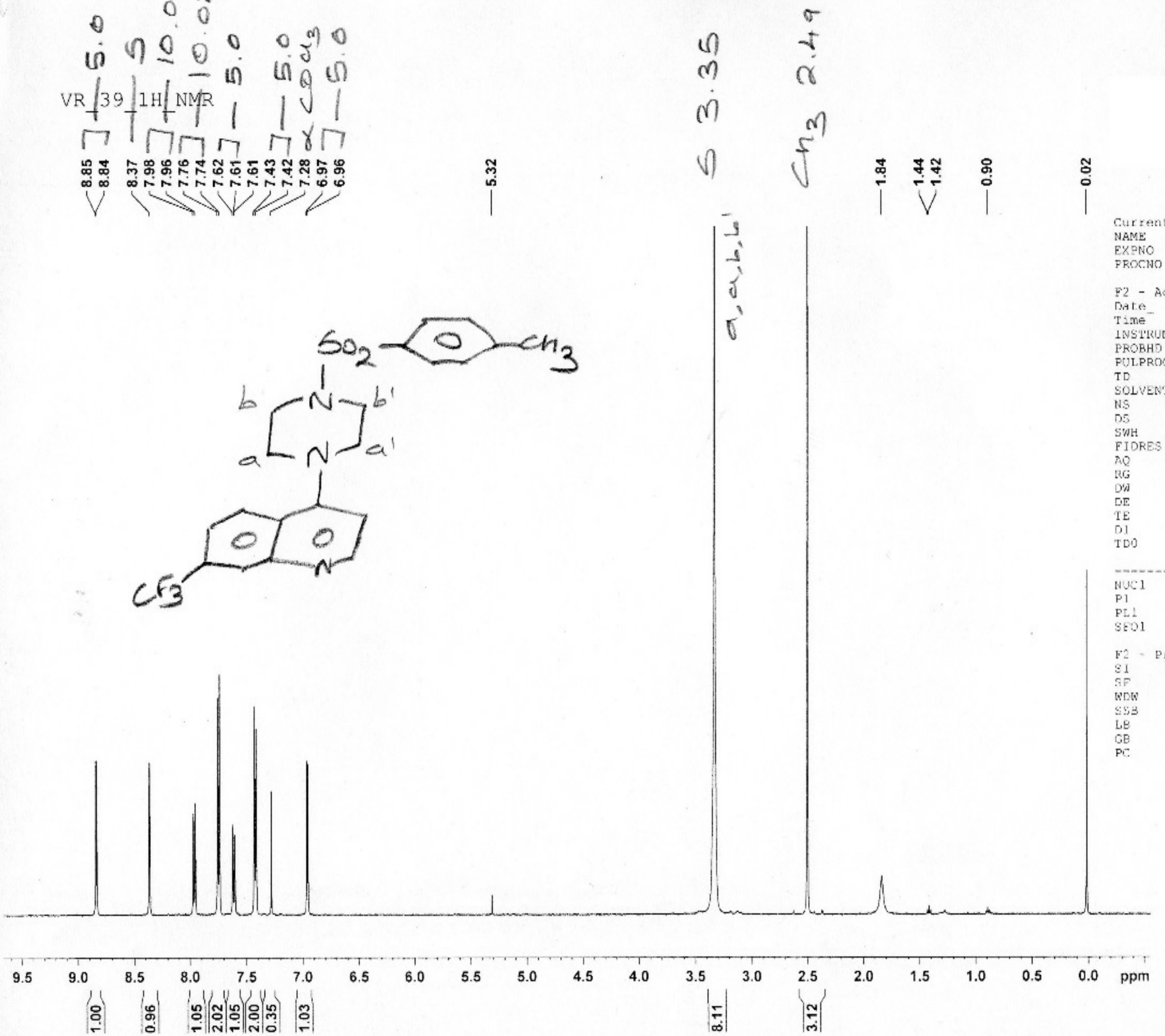

Current Data Parameters
NAME VR_39_1H_NMR
EXPNO 1
PROCNO 1

F2 - Acquisition Parameters
Date_ 20081003
Time 14.20
INSTRUM spect
PROBHD 5 mm Multinucl
PULPROG zg30
TD 65536
SOLVENT CDCl3
NS 64
DS 2
SWH 10330.578 Hz
FIDRES 0.157632 Hz
AQ 3.1719923 sec
RG 29193
DW 48.400 usec
DE 6.00 usec
TE 298.0 K
D1 1.00000000 sec
TD0 1

======== CHANNEL f1 ========
NUC1 1H
P1 7.35 usec
PL1 -6.00 dB
SFO1 500.1530890 MHz

F2 - Processing parameters
SI 32768
SF 500.1500000 MHz
WDW EM
SSB 0
LB 0.30 Hz
GB 0
PC 1.00

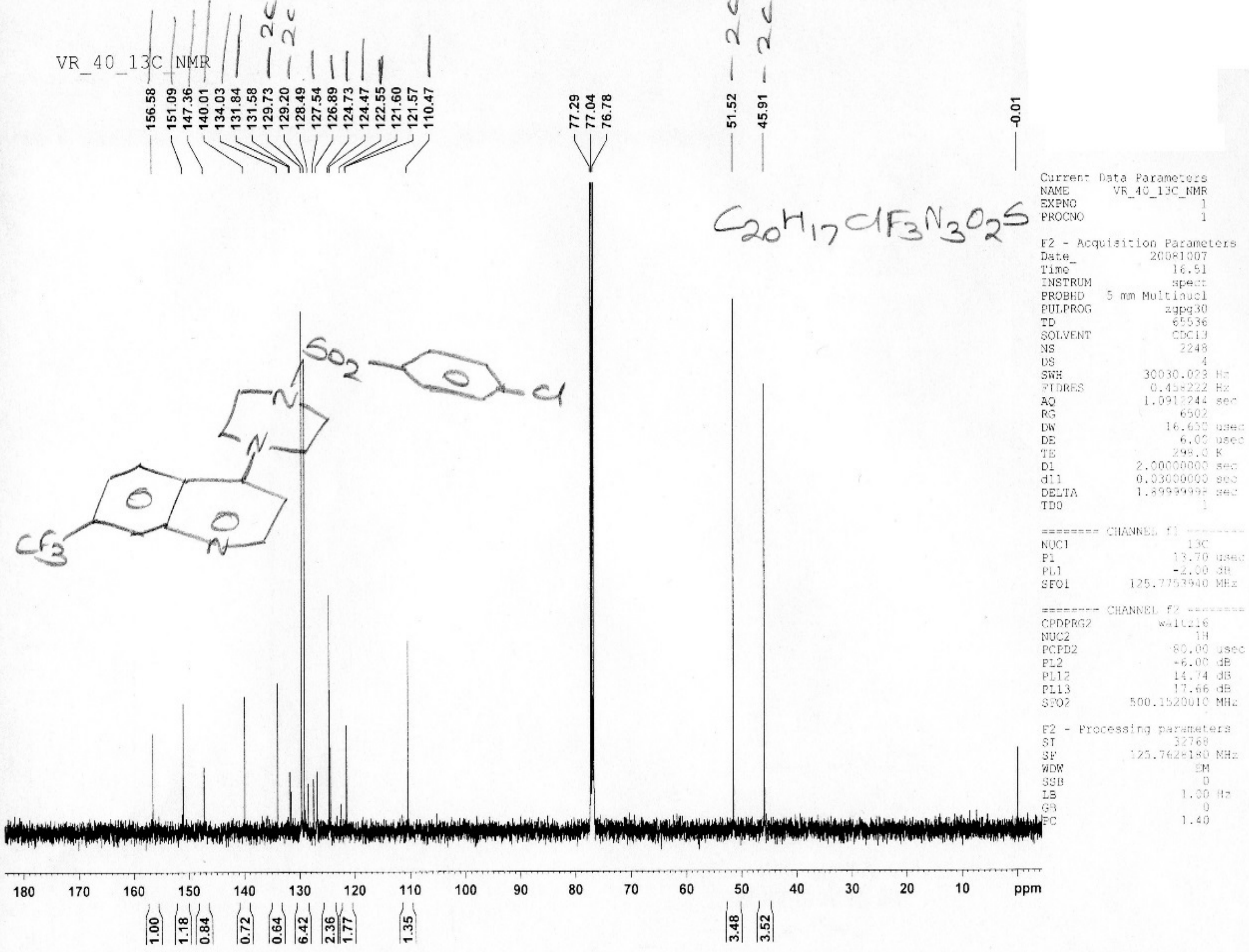

VR_40_13C_NMR
156.58
151.09
147.36
140.01
134.03
131.84
131.58
129.73
129.20
128.49
127.54
126.89
124.73
124.47
122.55
121.60
121.57
110.47
77.29
77.04
76.78
51.52
45.91
-0.01
2C
2C
2C
2C
SO2
Cl
N
N
N
CF3
C20H17ClF3N3O2S
180 170 160 150 140 130 120 110 100 90 80 70 60 50 40 30 20 10 ppm
1.00
1.18
0.84
0.72
0.64
6.42
2.36
1.77
1.35
3.48
3.52
Current Data Parameters
NAME VR_40_13C_NMR
EXPNO 1
PROCNO 1
F2 - Acquisition Parameters
Date_ 20081007
Time 16.51
INSTRUM spect
PROBHD 5 mm Multinucl
PULPROG zgpg30
TD 65536
SOLVENT CDCl3
NS 2248
DS 4
SWH 30030.029 Hz
FIDRES 0.458222 Hz
AQ 1.0912244 sec
RG 6502
DW 16.650 usec
DE 6.00 usec
TE 298.0 K
D1 2.00000000 sec
d11 0.03000000 sec
DELTA 1.89999998 sec
TD0 1
======== CHANNEL f1 ========
NUC1 13C
P1 13.70 usec
PL1 -2.00 dB
SFO1 125.7753940 MHz
======== CHANNEL f2 ========
CPDPRG2 waltz16
NUC2 1H
PCPD2 80.00 usec
PL2 -6.00 dB
PL12 14.74 dB
PL13 17.66 dB
SFO2 500.1520010 MHz
F2 - Processing parameters
SI 32768
SF 125.7628180 MHz
WDW EM
SSB 0
LB 1.00 Hz
GB 0
PC 1.40

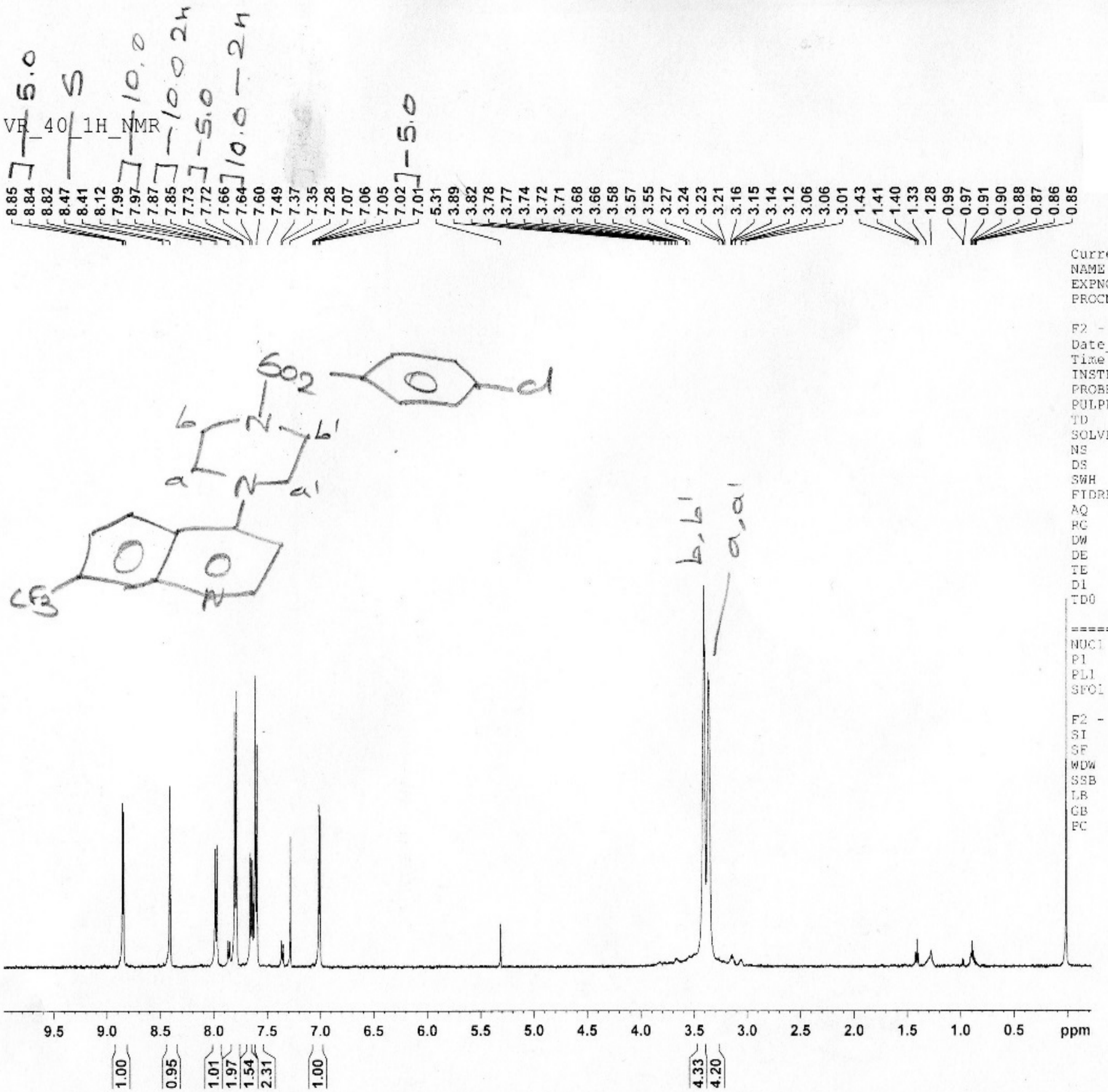


```
Current Data Parameters
NAME          VR_40_1H_NMR
EXPNO                    1
PROCNO                   1

F2 - Acquisition Parameters
Date_             20081007
Time                 14.44
INSTRUM              spect
PROBHD   5 mm Multinucl
PULPROG               zg30
TD                   65536
SOLVENT              CDCl3
NS                      64
DS                       2
SWH             10330.578 Hz
FIDRES           0.157632 Hz
AQ              3.1719923 sec
RG                   32768
DW                  48.400 usec
DE                    6.00 usec
TE                   298.0 K
D1              1.00000000 sec
TD0                      1

======== CHANNEL f1 ========
NUC1                    1H
P1                    7.35 usec
PL1                  -6.00 dB
SFO1           500.1530890 MHz

F2 - Processing parameters
SI                   32768
SF             500.1500000 MHz
WDW                     EM
SSB                      0
LB                    0.30 Hz
GB                       0
PC                    1.00
```

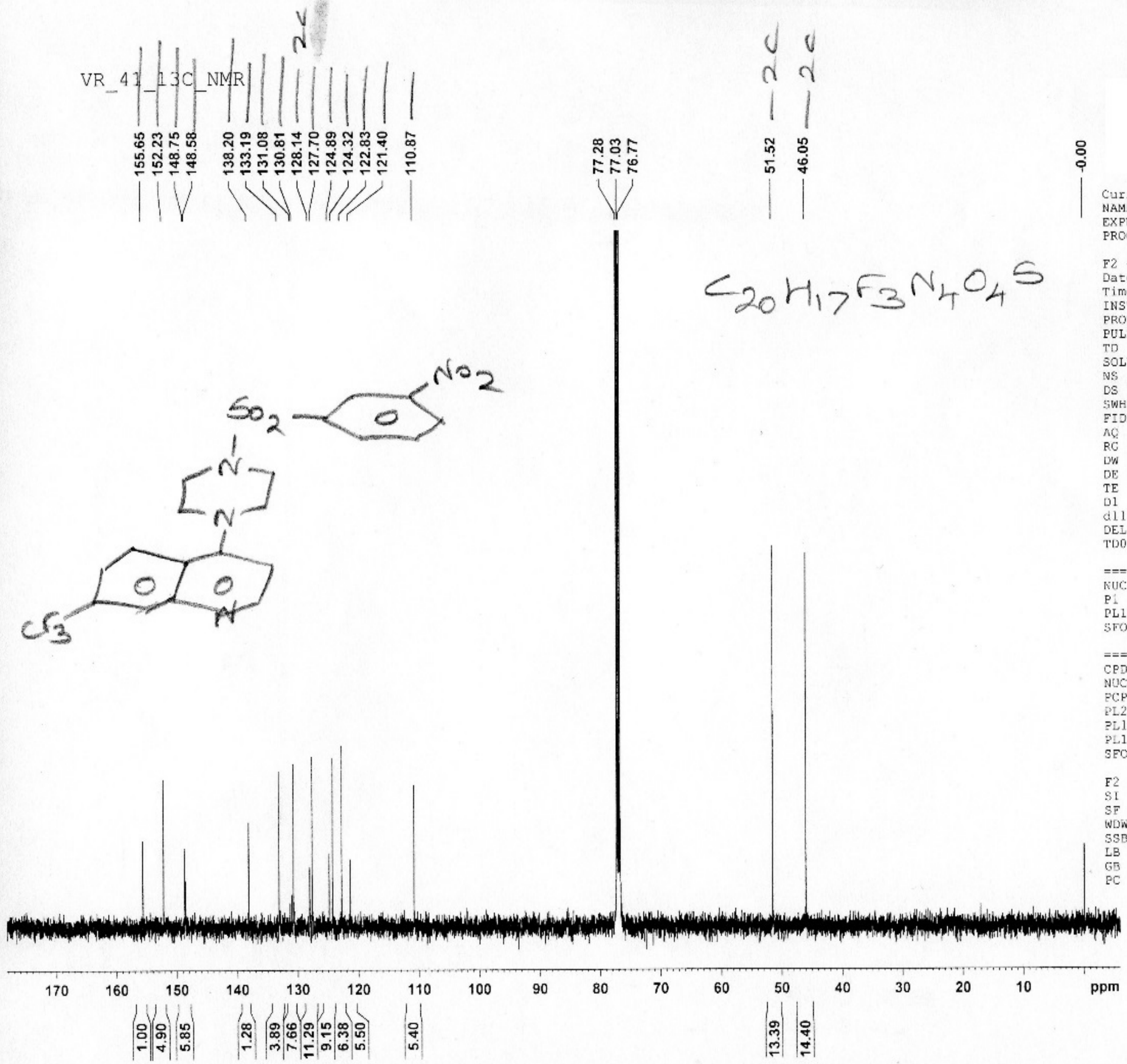

Current Data Parameters
NAME VR_41_13C_NMR
EXPNO 1
PROCNO 1

F2 - Acquisition Parameters
Date_ 20081008
Time 15.36
INSTRUM spect
PROBHD 5 mm Multinucl
PULPROG zgpg30
TD 65536
SOLVENT CDCl3
NS 2256
DS 4
SWH 30030.029 Hz
FIDRES 0.458222 Hz
AQ 1.0912244 sec
RG 8192
DW 16.650 usec
DE 6.00 usec
TE 298.0 K
D1 2.00000000 sec
d11 0.03000000 sec
DELTA 1.89999998 sec
TD0 1

======== CHANNEL f1 ========
NUC1 13C
P1 13.70 usec
PL1 -2.00 dB
SFO1 125.7753940 MHz

======== CHANNEL f2 ========
CPDPRG2 waltz16
NUC2 1H
PCPD2 80.00 usec
PL2 -6.00 dB
PL12 14.74 dB
PL13 17.66 dB
SFO2 500.1520010 MHz

F2 - Processing parameters
SI 32768
SF 125.7628180 MHz
WDW EM
SSB 0
LB 1.00 Hz
GB 0
PC 1.40

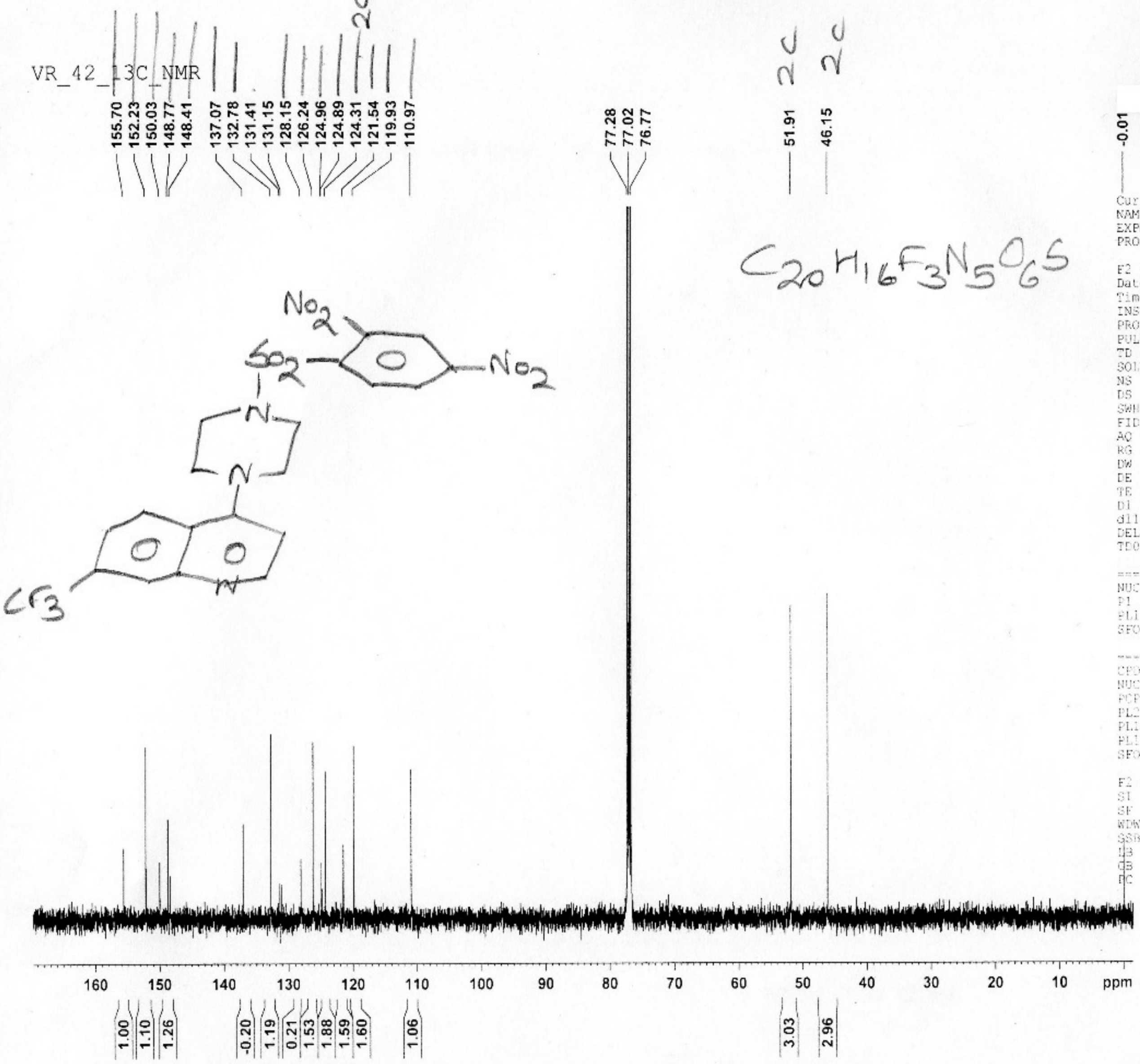


```
Current Data Parameters
NAME          VR_42_13C_NMR
EXPNO                     1
PROCNO                    1

F2 - Acquisition Parameters
Date_              20081010
Time                  15.56
INSTRUM               spect
PROBHD   5 mm Multinucl
PULPROG             zgpg30
TD                    65536
SOLVENT               CDCl3
NS                     2296
DS                        4
SWH               30030.029 Hz
FIDRES             0.458222 Hz
AQ                1.0912244 sec
RG                   4597.6
DW                   16.650 usec
DE                     6.00 usec
TE                    298.0 K
D1               2.00000000 sec
d11              0.03000000 sec
DELTA            1.89999998 sec
TD0                       1

======== CHANNEL f1 ========
NUC1                    13C
P1                    13.70 usec
PL1                   -2.00 dB
SFO1         125.7753940 MHz

======== CHANNEL f2 ========
CPDPRG2             waltz16
NUC2                     1H
PCPD2                 80.00 usec
PL2                   -6.00 dB
PL12                  14.74 dB
PL13                  17.66 dB
SFO2         500.1520010 MHz

F2 - Processing parameters
SI                    32768
SF           125.7628190 MHz
WDW                      EM
SSB                       0
LB                     1.00 Hz
GB                        0
PC                     1.40
```

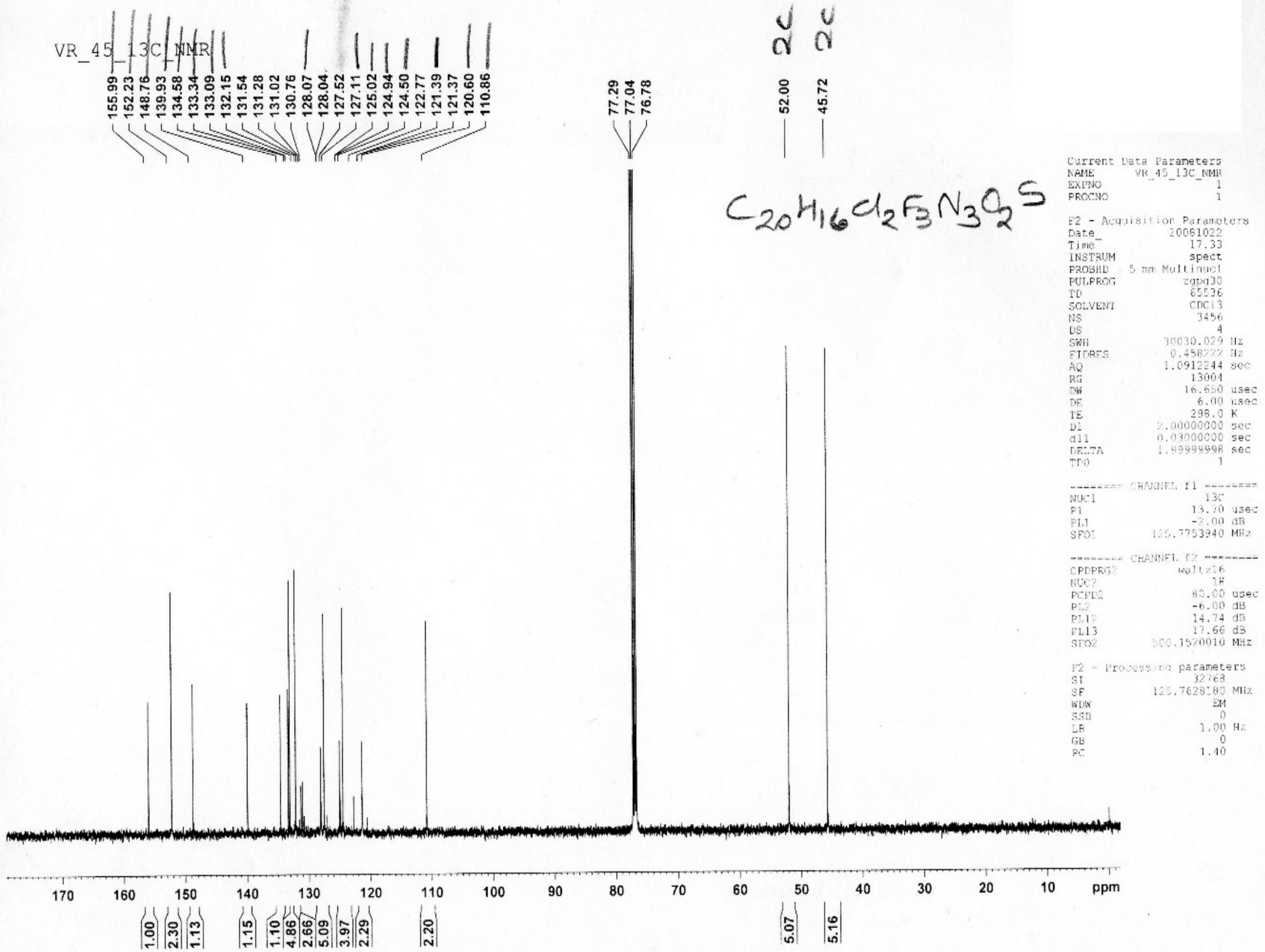
VR_45_13C_NMR
155.99
152.23
148.76
139.93
134.58
133.34
133.09
132.15
131.54
131.28
131.02
130.76
128.07
128.04
127.52
127.11
125.02
124.94
124.50
122.77
121.39
121.37
120.60
110.86
77.29
77.04
76.78
52.00
45.72
2C
2C
C20H16Cl2F3N3O2S
Current Data Parameters
NAME VR_45_13C_NMR
EXPNO 1
PROCNO 1
F2 - Acquisition Parameters
Date_ 20061022
Time 17.33
INSTRUM spect
PROBHD 5 mm Multinucl
PULPROG zgpg30
TD 65536
SOLVENT CDCl3
NS 3456
DS 4
SWH 30030.029 Hz
FIDRES 0.458222 Hz
AQ 1.0912244 sec
RG 13004
DW 16.650 usec
DE 6.00 usec
TE 298.0 K
D1 2.00000000 sec
d11 0.03000000 sec
DELTA 1.89999998 sec
TD0 1
======== CHANNEL f1 ========
NUC1 13C
P1 13.70 usec
PL1 -2.00 dB
SFO1 125.7753940 MHz
======== CHANNEL f2 ========
CPDPRG2 waltz16
NUC2 1H
PCPD2 80.00 usec
PL2 -6.00 dB
PL12 14.74 dB
PL13 17.66 dB
SFO2 500.1520010 MHz
F2 - Processing parameters
SI 32768
SF 125.7628180 MHz
WDW EM
SSB 0
LB 1.00 Hz
GB 0
PC 1.40
170 160 150 140 130 120 110 100 90 80 70 60 50 40 30 20 10 ppm
1.00
2.30
1.13
1.15
1.10
4.86
2.66
5.09
3.97
2.29
2.20
5.07
5.16

VR_45_1H_NMR

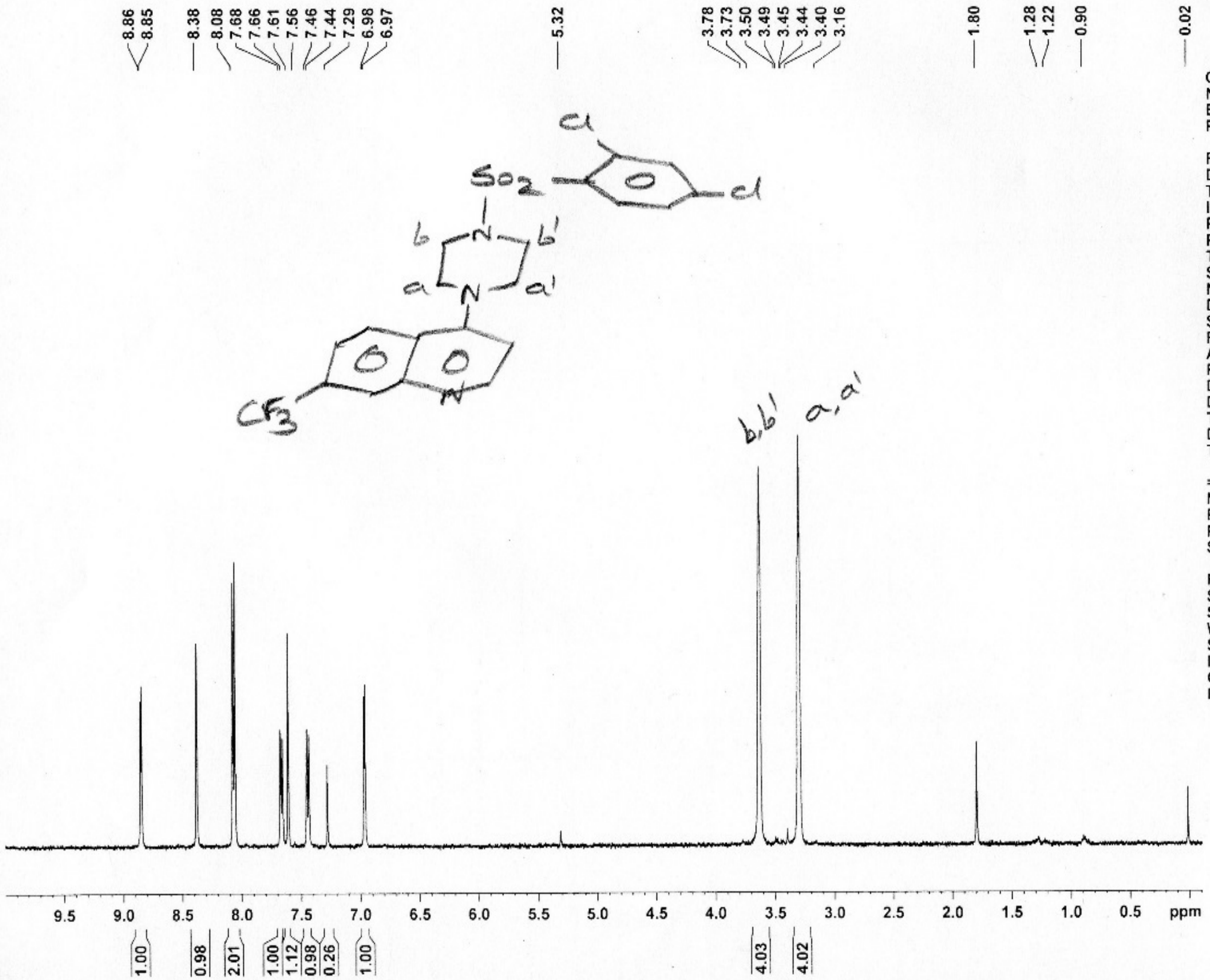

Current Data Parameters
NAME VR_45_1H_NMR
EXPNO 2
PROCNO 1

F2 - Acquisition Parameters
Date_ 20081022
Time 10.31
INSTRUM spect
PROBHD 5 mm Multinucl
PULPROG zg30
TD 65536
SOLVENT CDCl3
NS 64
DS 2
SWH 10330.578 Hz
FIDRES 0.157632 Hz
AQ 3.1719923 sec
RG 46341
DW 48.400 usec
DE 6.00 usec
TE 298.0 K
D1 1.00000000 sec
TD0 1

======== CHANNEL f1 ========
NUC1 1H
P1 7.35 usec
PL1 -6.00 dB
SFO1 500.1530890 MHz

F2 - Processing parameters
SI 32768
SF 500.1500000 MHz
WDW EM
SSB 0
LB 0.30 Hz
GB 0
PC 1.00

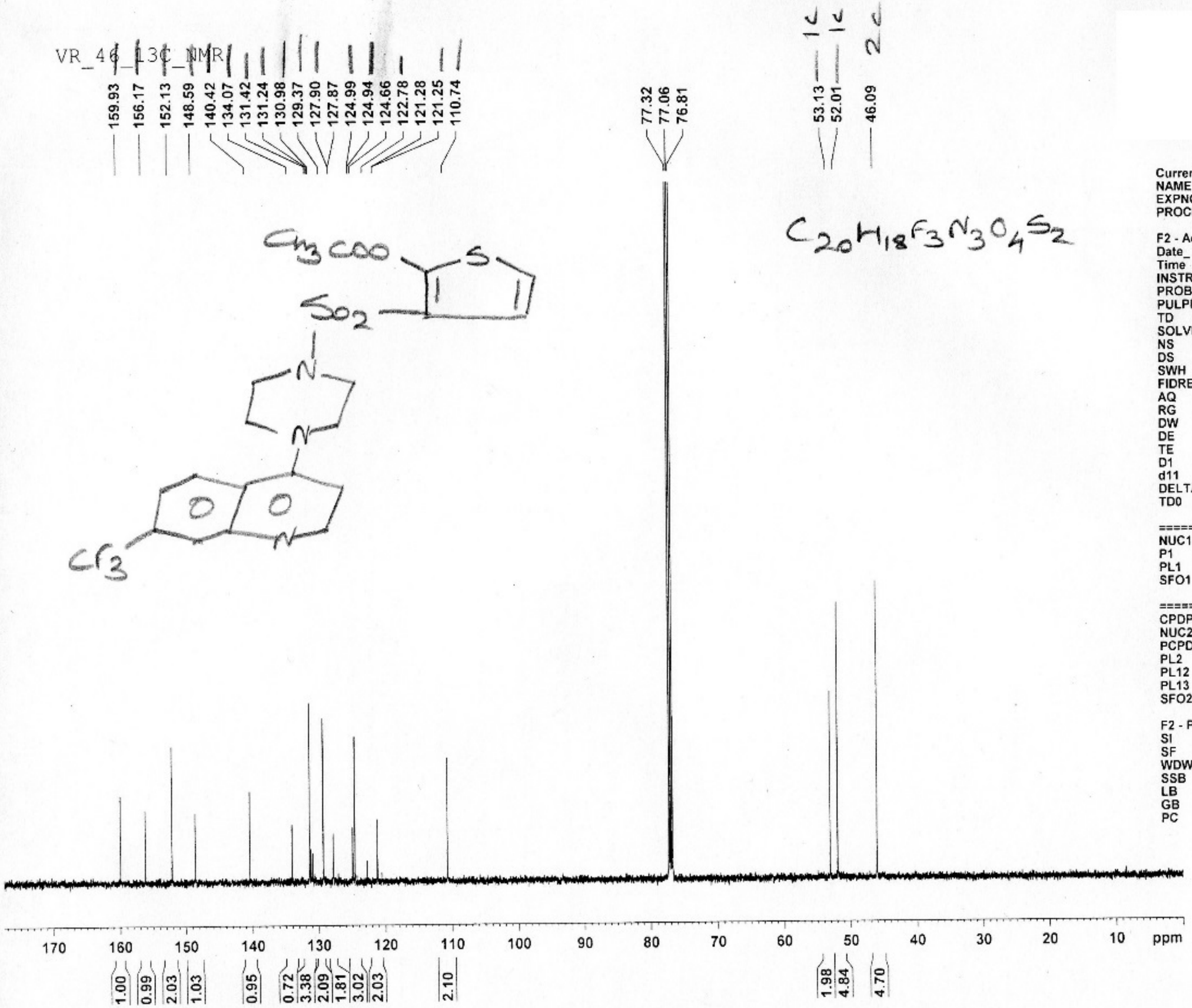

Current Data Parameters
NAME VR_46_13C_NMR
EXPNO 1
PROCNO 1

F2 - Acquisition Parameters
Date_ 20081021
Time 13.06
INSTRUM spect
PROBHD 5 mm Multinucl
PULPROG zgpg30
TD 65536
SOLVENT CDCl3
NS 1234
DS 4
SWH 30030.029 Hz
FIDRES 0.458222 Hz
AQ 1.0912244 sec
RG 4096
DW 16.650 usec
DE 6.00 usec
TE 298.0 K
D1 2.00000000 sec
d11 0.03000000 sec
DELTA 1.89999998 sec
TD0 1

======== CHANNEL f1 ========
NUC1 13C
P1 13.70 usec
PL1 -2.00 dB
SFO1 125.7753940 MHz

======== CHANNEL f2 ========
CPDPRG2 waltz16
NUC2 1H
PCPD2 80.00 usec
PL2 -6.00 dB
PL12 14.74 dB
PL13 17.66 dB
SFO2 500.1520010 MHz

F2 - Processing parameters
SI 32768
SF 125.7628180 MHz
WDW EM
SSB 0
LB 1.00 Hz
GB 0
PC 1.40

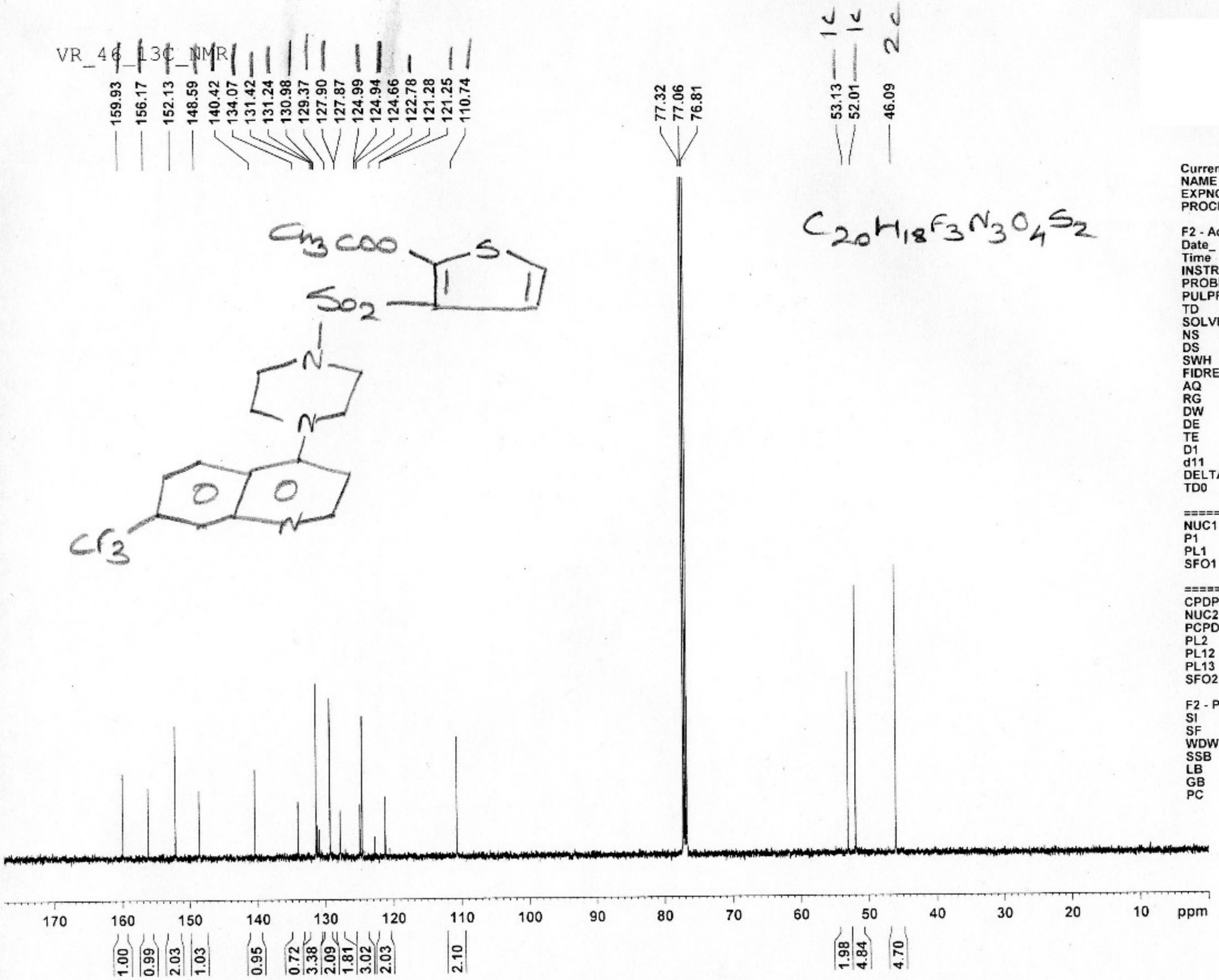

Current Data Parameters
NAME VR_46_13C_NMR
EXPNO 1
PROCNO 1

F2 - Acquisition Parameters
Date_ 20081021
Time 13.06
INSTRUM spect
PROBHD 5 mm Multinucl
PULPROG zgpg30
TD 65536
SOLVENT CDCl3
NS 1234
DS 4
SWH 30030.029 Hz
FIDRES 0.458222 Hz
AQ 1.0912244 sec
RG 4096
DW 16.650 usec
DE 6.00 usec
TE 298.0 K
D1 2.00000000 sec
d11 0.03000000 sec
DELTA 1.89999998 sec
TD0 1

======== CHANNEL f1 ========
NUC1 13C
P1 13.70 usec
PL1 -2.00 dB
SFO1 125.7753940 MHz

======== CHANNEL f2 ========
CPDPRG2 waltz16
NUC2 1H
PCPD2 80.00 usec
PL2 -6.00 dB
PL12 14.74 dB
PL13 17.66 dB
SFO2 500.1520010 MHz

F2 - Processing parameters
SI 32768
SF 125.7628180 MHz
WDW EM
SSB 0
LB 1.00 Hz
GB 0
PC 1.40

VR_46_1H_NMR

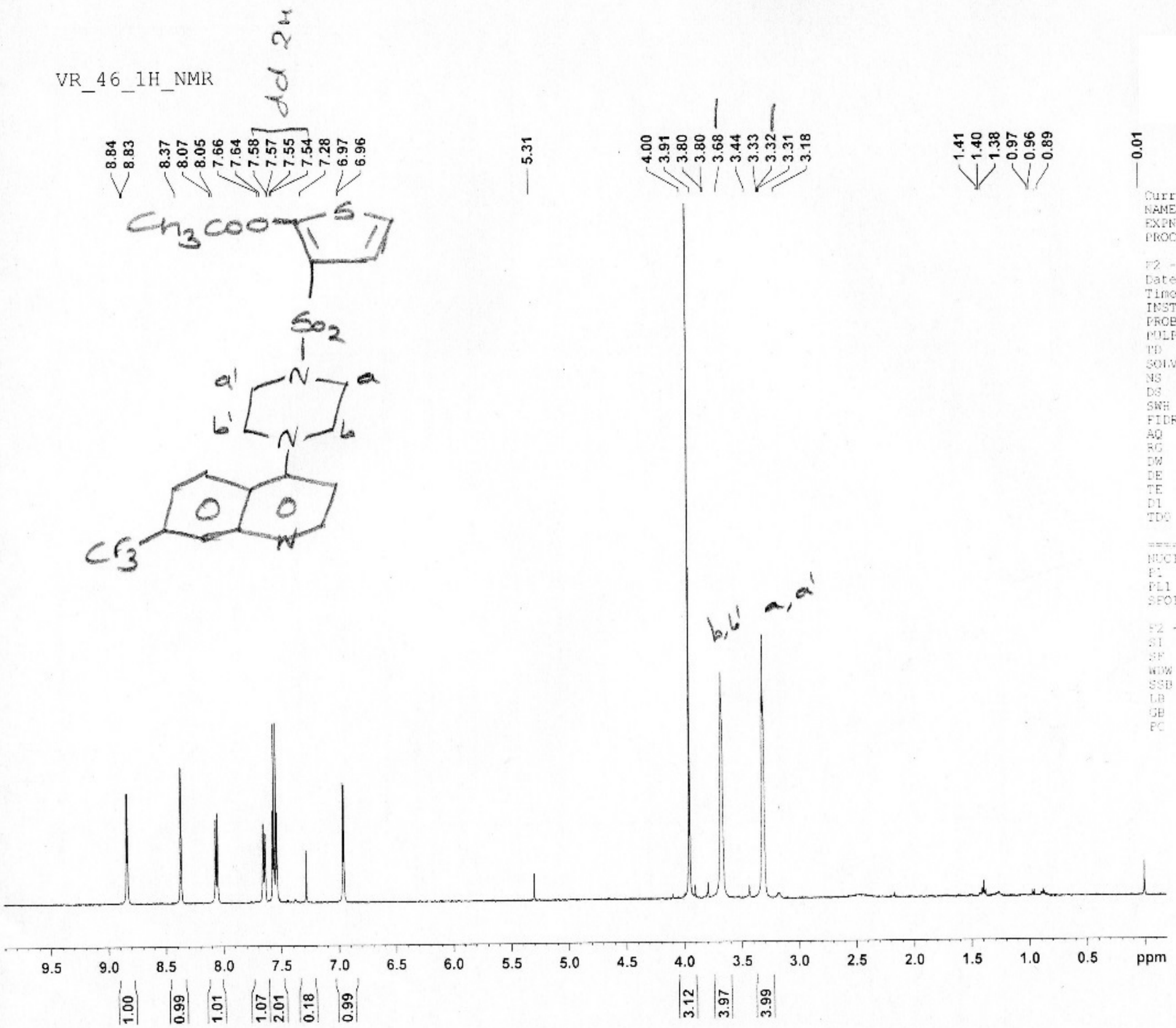


```
Current Data Parameters
NAME         VR_46_1H_NMR
EXPNO                   1
PROCNO                  1

F2 - Acquisition Parameters
Date_            20081021
Time                11.46
INSTRUM             spect
PROBHD   5 mm Multinucl
PULPROG              zg30
TD                  65536
SOLVENT             CDCl3
NS                     64
DS                      2
SWH             10330.578 Hz
FIDRES           0.157632 Hz
AQ              3.1719923 sec
RG                36780.8
DW                 48.400 usec
DE                   6.00 usec
TE                  298.0 K
D1             1.00000000 sec
TD0                     1

======== CHANNEL f1 ========
NUC1                   1H
P1                   7.35 usec
PL1                 -6.00 dB
SFO1          500.1530890 MHz

F2 - Processing parameters
SI                  32768
SF            500.1500000 MHz
WDW                    EM
SSB                     0
LB                   0.30 Hz
GB                      0
PC                   1.00
```

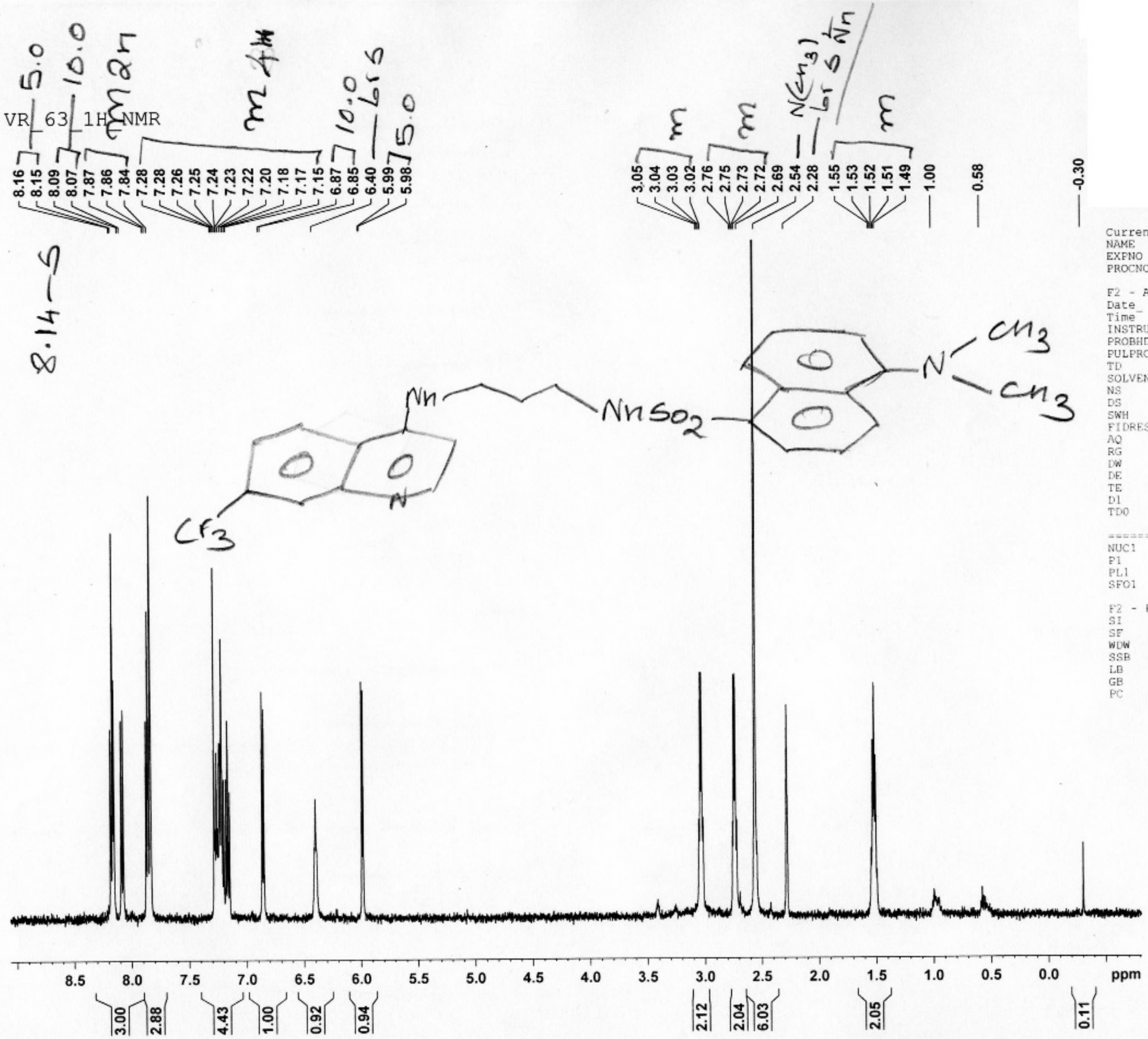

Current Data Parameters
NAME VR_63_1H_NMR
EXPNO 1
PROCNO 1

F2 - Acquisition Parameters
Date_ 20081209
Time 14.40
INSTRUM spect
PROBHD 5 mm Multinucl
PULPROG zg30
TD 65536
SOLVENT CDCl3
NS 64
DS 2
SWH 10330.578 Hz
FIDRES 0.157632 Hz
AQ 3.1719923 sec
RG 46341
DW 48.400 usec
DE 6.00 usec
TE 298.0 K
D1 1.00000000 sec
TD0 1

======== CHANNEL f1 ========
NUC1 1H
P1 7.35 usec
PL1 -6.00 dB
SFO1 500.1530890 MHz

F2 - Processing parameters
SI 32768
SF 500.1500000 MHz
WDW EM
SSB 0
LB 0.30 Hz
GB 0
PC 1.00
